# Investigating a Library of Flavonoids as Potential Inhibitors of a Cancer Therapeutic Target MEK2 Using in Silico Methods

**DOI:** 10.3390/ijms24054446

**Published:** 2023-02-23

**Authors:** Wejdan M. AlZahrani, Shareefa A. AlGhamdi, Sayed S. Sohrab, Mohd Rehan

**Affiliations:** 1 Department of Biochemistry, Faculty of Sciences, King Abdulaziz University, Jeddah 21589, Saudi Arabia; 2 Special Infectious Agents Unit-BSL3, King Fahd Medical Research Center, King Abdulaziz University, Jeddah 21589, Saudi Arabia; 3 Department of Medical Laboratory Sciences, Faculty of Applied Medical Sciences, King Abdulaziz University, Jeddah 21589, Saudi Arabia; 4 King Fahd Medical Research Center, King Abdulaziz University, Jeddah 21589, Saudi Arabia

**Keywords:** MAPK, MEK2, flavonoids, molecular docking, ADMET, molecular dynamics simulation

## Abstract

The second leading cause of death in the world is cancer. Mitogen-activated protein kinase (MAPK) and extracellular signal-regulated protein kinase (ERK) 1 and 2 (MEK1/2) stand out among the different anticancer therapeutic targets. Many MEK1/2 inhibitors are approved and widely used as anticancer drugs. The class of natural compounds known as flavonoids is well-known for their therapeutic potential. In this study, we focus on discovering novel inhibitors of MEK2 from flavonoids using virtual screening, molecular docking analyses, pharmacokinetic prediction, and molecular dynamics (MD) simulations. A library of drug-like flavonoids containing 1289 chemical compounds prepared in-house was screened against the MEK2 allosteric site using molecular docking. The ten highest-scoring compounds based on docking binding affinity (highest score: −11.3 kcal/mol) were selected for further analysis. Lipinski’s rule of five was used to test their drug-likeness, followed by ADMET predictions to study their pharmacokinetic properties. The stability of the best-docked flavonoid complex with MEK2 was examined for a 150 ns MD simulation. The proposed flavonoids are suggested as potential inhibitors of MEK2 and drug candidates for cancer therapy.

## 1. Introduction

The second greatest cause of mortality in the United States and a major global public health concern is cancer [1]. Cancer cells are recognized by eight hallmarks: evading growth suppressors, sustaining proliferative signaling, resisting cell death, inducing/accessing vasculature, enabling replicative immortality, activating invasion and metastasis, avoiding immune destruction, and reprogramming cellular metabolism [2]. The majority of oncogenes that have been discovered so far produce protein kinases, and the growth and survival of cancer depend on the deregulation of these kinases’ activity [3]. Mitogen-activated protein kinase (MAPK) cascade is an important pathway in the growth and development of cells. It is comprised of three kinases: Raf, MEK and ERK [4]. The deregulation of the MAPK cascade leads to uncontrolled cancer cell proliferation and survival; therefore, it is exploited as a rational therapeutic target for various cancer conditions [5]. After the receptor activation, RAS binds GTP to form heterodimers of RAF protein kinases. MEK1 and MEK2, two dual-specificity kinases, are then directly phosphorylated and activated to activate ERK1 and ERK2 via phosphorylation [6]. MEK1 and MEK2 (also known as MAP2K1 and MAP2K2, respectively) are dual specificity protein kinases, having both Ser/Thr and Tyr kinase activities. They are the only activator of ERK1/2, which in turn have many targets controlling cancer cell survival, differentiation and progression [5,7]. MEK1/2 provide valuable targets as they hold a unique inhibition binding pocket, separate from the ATP-binding pocket represented through a conserved region called DFG-out (Asp-Phe-Gly) [6,8], that inhibitors can bind to in an ATP-noncompetitive way and can lock the protein in an inactive state, which gives higher selectivity than any other kinase targets [9].

MEK1 and MEK2 form heterodimers with high homology amino acids and are considered to be redundant enzymes [10]. However, MEK1 can be activated through different scaffold proteins and regulated through ERK-mediated feedback phosphorylation on Thr292, a residue that MEK2 lacks [6]. Negative feedback is disabled if MEK1 is missing or unable to bind to MEK2; thus, ERK activation and phosphorylation through MEK2 are prolonged [7]. Mice with *Mek1* knockout die during embryogenesis, while levels of Mek2 and Erk activation were normal [11]. In *Mek2* knockout mice, they had normal development of the embryo, with Mek2 activity being compensated with Mek1 [12]. In pancreatic cancer, MEK2 was reported to be related to the first step of the cascade in invasion-metastasis [13]. MEK2, and not MEK1, is sufficient for cell proliferation and anchorage-independent growth of SK-MEL-28 melanoma cells [14]. Moreover, MEK2 is sufficient to induce epidermal papilloma formation in zebrafish [15]. These reports suggest that these two enzymes may have different functions and different roles in tumorigenesis and are, therefore, excellent therapeutic targets.

Flavonoids are secondary plant metabolites that are polyphenolic and found in a wide range of foods, such as fruits and vegetables [16]. Flavonoids have a broad range of health benefits, including antioxidative, anti-mutagenic, anti-inflammatory, anti-carcinogenic, [17] anti-microbial, anti-bacterial, and anti-fungal activities [18]. Flavonoids inhibit proliferation, angiogenesis, and metastasis or induce apoptosis, autophagy, and cell cycle arrest by interfering with a number of signal transduction pathways during the development of cancer [19,20,21]. In cell signaling pathways, some flavonoids can bind to protein kinases and alter their phosphorylation, such as MAP kinases. Myricetin, quercetin, (RSVL, a phytoalexin polyphenol), daidzein (equol), and delphinidin all can bind to MEK1 and inhibit the kinase activity without competing with ATP [22,23,24,25,26]. The first specific MEK1/2 inhibitor to be discovered was PD-098059 [2-(2-amino-3-methoxyphenyl)-4H-1-benzopyran-4-one], which is a flavone [27]. PD98059 inhibits MEK1 more selectively than MEK2 [28].

With the advancement of theoretical and computational methods, computer-aided drug design (CADD) has been extensively exploited to find new hits or leads against specific biologically active targets. CADD methods such as virtual screening, molecular docking, pharmacophore modeling, and molecular dynamics simulation approaches are widely used to discover, develop, and analyze drugs [29,30,31,32,33,34,35,36]. Virtual screening through molecular docking is used to shortlist compounds from a huge compound library against a target easily within a short time [37]. By using CADD, it is also possible to predict pharmacokinetics and pharmacology properties such as absorption, distribution, metabolism, and excretion (ADME), as well as the toxicity of a potential drug candidate [38].

In order to identify new potential inhibitors from the pool of the natural compound known as flavonoids against the therapeutic cancer target MEK2, this study is primarily focused on computer-aided drug design approaches, such as virtual screening, ADMET prediction, molecular docking, and molecular dynamics simulations.

## 2. Results and Discussion

### 2.1. Virtual Screening of Natural Compound Class Flavonoids for MEK2 Inhibition

**Figure 1:**
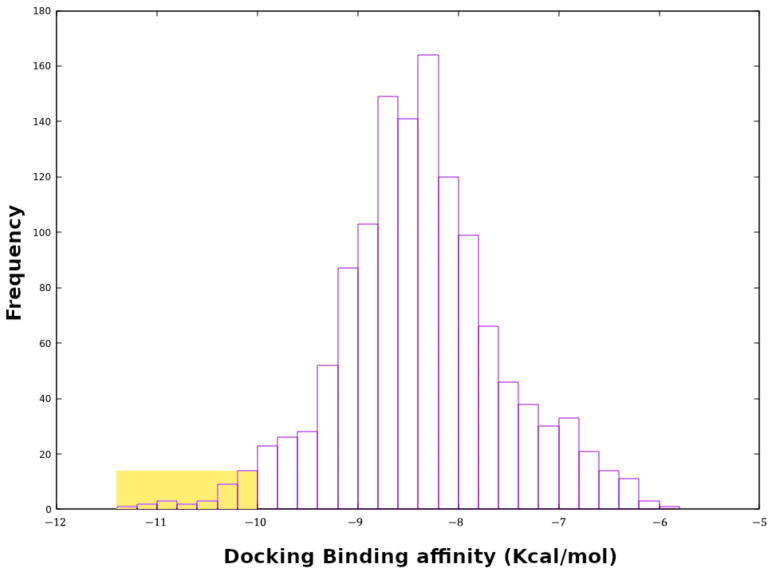
Histogram plot of dock-binding affinity of all flavonoids. The docking scores of the selected flavonoids in the left part are highlighted in yellow.

**Figure 2:**
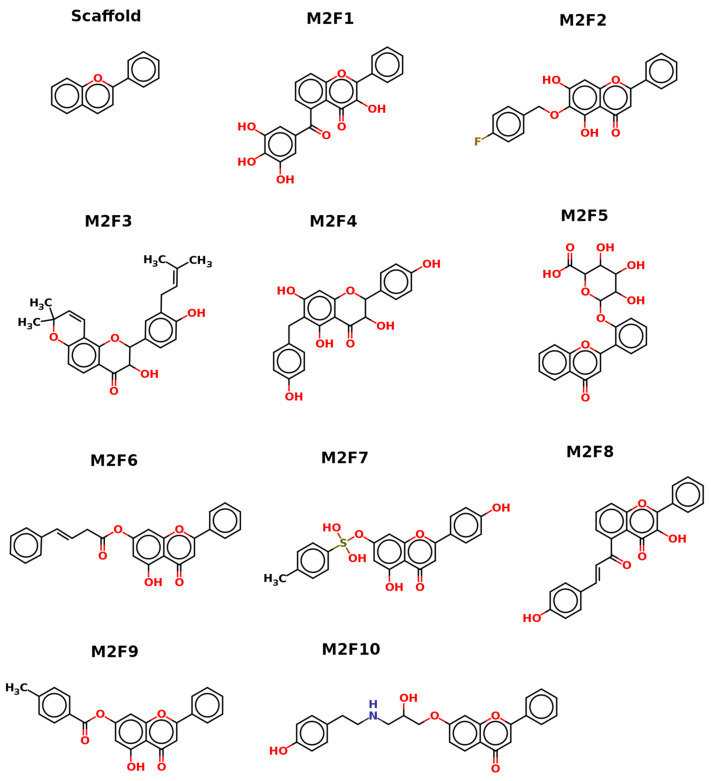
Two-dimensional sketch of the selected flavonoids for MEK2 inhibition. Oxygen (O), nitrogen (N), and sulfur (S) with their balancing hydrogens are shown as red, blue, and green, respectively.

**Figure 3:**
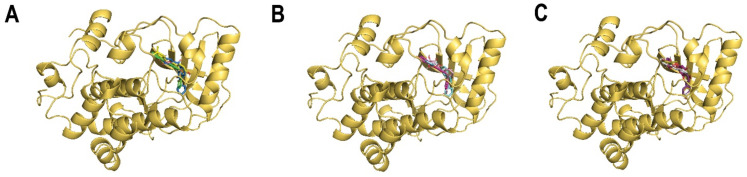
Molecular docking of top 10 selected flavonoids to MEK2. The proteins shown in cartoon representation are colored yellow-orange, while compounds are shown in stick representations. (**A**) Binding of flavonoids ‘M2F1’ (blue), ‘M2F2’ (green), and ‘M2F3’ (yellow). (**B**) Binding of flavonoids ‘M2F4’ (pink), ‘M2F5’ (light-pink) and ‘M2F6’ (cyan). (**C**) Binding of flavonoids ‘M2F7’ (violet), ‘M2F8’ (white), ‘M2F9’ (magenta) and ‘M2F10’ (gold).

**Figure 4:**
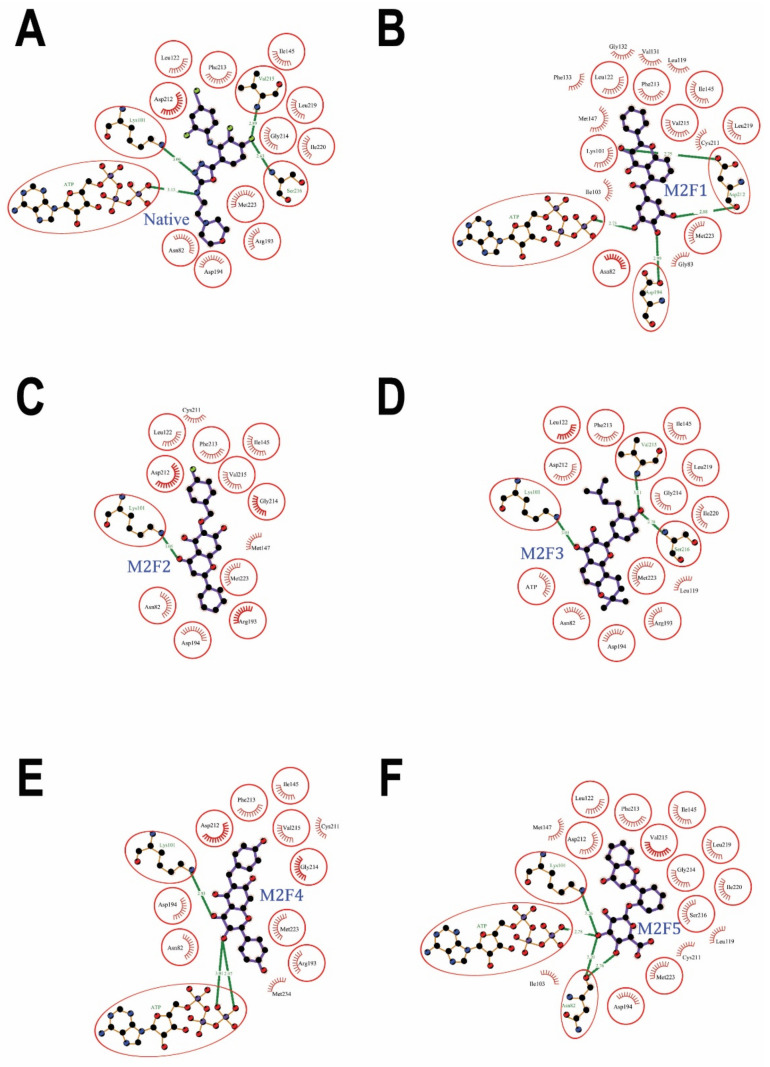
MEK2 protein–ligand interaction plots 1. Native inhibitor (**A**) and selected flavonoids (**B**–**F**). The residues forming non-bonding interactions are shown as red bristles, while residues forming hydrogen bonds and the bound ligand are shown as ball-and-stick representations. The carbon atoms are shown as black balls, nitrogen atoms as blue balls, oxygen atoms as red balls, and sulfur atoms as yellow balls. The interacting residues in common with the native inhibitor are shown in circles. The hydrogen bonds are shown as green dashed lines labeled with bond length (in Å).

- In silico drug discovery using molecular docking has an important role in evaluating the binding ability of ligands to the target protein. In order to find potential inhibitors of MEK2, which are critical target proteins in the MAPK pathway in cancer cells, we screened a flavonoid library using molecular docking. The histogram in Figure 1 shows the results of the virtual screening (Supplementary File Table S1), where the best and 10 highest-ranked compounds are highlighted on the left. The majority of the data show good docking affinity (<−7.0 kcal/mol), but the redundant compounds were removed, and the best-scoring fitting compounds were selected. The top 10 selected flavonoids against MEK2 in (Figure 2) show the best docking score of −11.3 kcal/mol, with the 10th ranking compound demonstrating a score of −10.4 kcal/mol, as compared to the docking score of the native inhibitor of −11.0 kcal/mol. The native inhibitor of the protein-crystallized structure is PD334581 (5-{3,4-difluoro-2-[(2-fluoro-4-iodophenyl)amino]phenyl}-n-(2-morpholin-4-ylethyl)-1,3,4-oxadiazol-2-amine), an analogue of PD184352, which is a selective, ATP-noncompetitive, and potent inhibitor of MEK1 and MEK2 [8]. Some compounds showed higher docking scores than the native inhibitor, suggesting the selected proposed flavonoids are probably better inhibitors of MEK2. This is also in agreement with one of the molecular docking studies of trametinib, a highly specific and selective inhibitor of MEK2, which reported a docking score of −11.26 kcal/mol for MEK2 [39].
- The 10 highest-ranking compounds have been chosen as potential MEK2 inhibitors, and the descriptions of their docking results are as follows:

The first-ranked flavonoid **M2F1** (CID: 129703940) fit well in the MEK2 allosteric site (Figure 3A). The ligand interacted with ATP and 18 amino acids: Asn-82, Gly-83, Lys-101, Ile-103, Leu-119, Leu-122, Val-131, Gly-132, Phe-133, Ile-145, Met-147, Asp-194, Cys-211, Asp-212, Phe-213, Val-215, Leu-219, and Met-223 (Figure 4B) (Table 1). The molecular docking binding affinity was −11.3 Kcal/mol, the binding energy was −10.09 Kcal/mol, and the dissociation constant $pK_{d}$, with a value of 7.40, was reasonably high, as needed for the effective inhibition of MEK2 (Table 2). The interaction (Appendix A, Table A1) shows 4 hydrogen bonds and 65 non-bonded contacts (mainly hydrophobic interactions). The hydrogen bond with Asp-194 measures 2.99 Å; two hydrogen bonds with Asp-212 measure 2.75 Å and 2.88 Å, and one hydrogen bond with ATP measures 2.75 Å. The key interacting residues are Ile-145, Asp-212, Phe-213, Leu-219, and Met-223, forming 6, 6, 7, 6, and 6 non-bonding interactions, respectively. The Asp-212 showed the maximum ΔASA (48.59 Å^2^) upon binding. Comparing to the native inhibitor, of 18 residues, there were ten common residues besides ATP: Asn-82, Lys-101, Leu-122, Ile-145, Asp-194, Asp-212, Phe-213, Val-215, Leu-219, and Met-223 (Figure 4A) (Table 1), and they share the same key residues, Asp-212 and Phe-213. The fact that the selected compound shares the same amino acids as the native inhibitor suggests that they may inhibit the protein in the same way.

The second-ranked flavonoid **M2F2** (CID: 56649181) against the MEK2 binding pocket docked well in the allosteric site (Figure 3A). The ligand interaction with the protein showed 1 hydrogen bond and 56 non-bonded contacts (hydrophobic interactions) through 13 amino acids: Asn-82, Lys-101, Leu-122, Ile-145, Met-147, Arg-193, Asp-194, Cys-211, Asp-212, Phe-213, Gly-214, Val-215, and Met-223 (Figure 4C) (Table 1). The docking affinity was −11.1 Kcal/mol, the binding energy was −9.41 Kcal/mol, and the dissociation constant, $pK_{d}$, had a value of 6.90, showing as good quality binding as required for adequate inhibition (Table 2). The hydrogen bond with Lys-101 measured 3.05 Å. The key interacting residues are Asp-194, Asp-212 and Phe-213, forming 11, 17, and 6 non-bonding interactions of each, respectively. The Asp-212 turned out to have the maximum ΔASA (48.52 Å^2^) upon binding, followed by Asn-82 (38.46 Å^2^) and Met-223 with 36.8 Å^2^ (Appendix A, Table A2). Compared to the native inhibitor, of 13 interacting residues, there were 11 common residues: Asn-82, Lys-101, Leu-122, Ile-145, Arg-193, Asp-194, Asp-212, Phe-213, Val-215, Leu-219, and Met-223 (Figure 4A) (Table 1), and they share the same key residues, Asp-212 and Phe-213. The suggested compound holds similar potential as the native inhibitor because it binds to the same group of residues in the allosteric site.

The docking results of the third-ranked flavonoid **M2F3** (CID: 131751372) showed good fitting in the allosteric site of MEK2 (Figure 3A). The ligand interacted with 15 amino acids: Asn-82, Lys-101, Leu-122, Ile-145, Arg-193, Asp-194, Asp-212, Phe-213, Gly-214, Val-215, Ser-216, Leu-219, Ile-220, and Met-223 (Figure 4D). The molecular docking affinity was −11.1 Kcal/mol, the binding energy was −10.38 Kcal/mol, and the dissociation constant$,pK_{d}$, with a value of 7.61, indicates high quality binding for the effective inhibition of MEK2 (Table 2). The interaction shows 3 hydrogen bonds and 51 non-bonded contacts (mainly hydrophobic interactions). The hydrogen bond with Lys-101 measures 3.01 Å, Val-215 measures 3.11 Å, and Ser-216 measures 2.78 Å. The key interacting residues are Asp-212 and Val-215, forming 8 and 6 non-bonding interactions of each, respectively. Met-223 turned out to show the maximum ΔASA (51.44 Å^2^) upon binding, followed by Asp-212 (48.86 Å^2^) and Asn-82 (38.86 Å^2^) (Appendix A, Table A3). Compared to the native inhibitor of 15 residues, there are 14 common residues besides ATP: Asn-82, Lys-101, Leu-122, Ile-145, Arg-193, Asp-194, Asp-212, Phe-213, Gly-214, Val-215, Ser-216, Leu-219, Ile-220, and Met-223 (Figure 4A) (Table 1), and they share the same key residues, Asp-212 and Phe-213. This compound is a chiral compound and has the stereoisomeric configurations R-R, R-S, S-R, and S-S. The compound’s docking data are shown above in the S-R stereoisomer configuration. Molecular docking was used to examine other stereoisomers’ ability to bind to MEK2. The molecular docking of the R-R configuration of the chiral compound also showed good affinity, with −10.4 Kcal/mol, while the R-S configuration displayed an affinity of −10.7 Kcal/mol, and the S-S configuration had an affinity of −8.1 Kcal/mol. Nevertheless, there is not any literature on this compound’s stereoisomerism. This presents a good opportunity to investigate how the enantiomers of this compound affect its ability to bind to the MEK2 kinase and produce a racemic drug.

The dock results of the fourth-ranked flavonoid **M2F4** (CID: 44559902) against the MEK2 binding pocket bound to the allosteric site well (Figure 3B). The ligand interacted with ATP and 12 amino acid residues: Asn-82, Lys-101, Ile-145, Arg-193, Asp-194, Cys-211, Asp-212, Phe-213, Gly-214, Val-215, Met-223, and Met-234 (Figure 4E) (Table 1). Molecular docking data show a docking affinity of −10.9 Kcal/mol, a binding energy of −9.26 Kcal/mol, and a dissociation constant$,pK_{d}$, with a value of 6.79, which was also quite high and was essential for effective MEK2 kinase inhibition (Table 2). The interaction shows three hydrogen bonds and 57 non-bonded contacts (mainly hydrophobic interactions). The hydrogen bond with Lys-101 measures 2.93 Å, and the two hydrogen bonds with ATP measure: 2.97 Å and 3.01 Å. The key interacting residues are Asp-194, Asp-212, and Phe-213, forming 9, 18, and 9 non-bonding interactions of each, respectively. The Asp-212 turned out to have the maximum ΔASA (48.86 Å^2^) upon binding, followed by Asn-82 (40.58 Å^2^), Asp-194 (38.11 Å^2^), and Met-223 (37.38 Å^2^) (Appendix A, Table A4). Of the 12 interacting residues, 12 residues besides ATP were common among the interacting residues of the native inhibitor: Asn-82, Lys-101, Ile-145, Arg-193, Asp-194, Asp-212, Phe-213, Gly-214, Val-215, and Met-223 (Figure 4A) (Table 1), and they share the same key residues, Asp-212 and Phe-213. They may therefore inhibit MEK2 kinase activity in a manner similar to the native inhibitor. This compound is a chiral compound and possesses R-R, R-S, S-R and S-S stereoisomeric configurations. The above docking results of this compound are in the S-S stereoisomer configuration. In order to check the effect of other stereoisomers of this compound on the binding to MEK2, molecular docking was performed. The molecular docking of the R-R configuration of the chiral compound also showed high affinity at −9.1 Kcal/mol, while the R-S configuration displayed an affinity of −9.1 Kcal/mol, and the S-R configuration displayed an affinity of −10.3 Kcal/mol. This compound introduces a good chance to study the effect of enantiomers on the binding to the MEK2 kinase and produce a racemic compound.

The fifth-ranked flavonoid **M2F5** (CID: 154699598) bound well in the MEK2 allosteric site (Figure 3B). The ligand interacted with ATP and 17 amino acids: Asn-82, Lys-101, Ile-103, Leu-119, Leu-122, Ile-145, Met-147, Asp-194, Cys-211, Asp-212, Phe-213, Gly-214, Val-215, Ser-216, Leu-219, Ile-220, and Met-223 (Figure 4F) (Table 1). The molecular docking results show a docking affinity of −10.9 Kcal/mol, a binding energy of −10.02 Kcal/mol, and a dissociation constant$,pK_{d}$, of 7.34, showing good quality binding as required for the adequate inhibition of MEK2 (Table 2). The interaction shows 4 hydrogen bonds and 69 non-bonded contacts (mainly hydrophobic interactions). The hydrogen bonds with Asn-82 measure 2.76 Å and 3.20 Å, the hydrogen bond with Lys-101 measures 3.26 Å, and one hydrogen bond with ATP measures 2.78 Å. The key interacting residues are Ile-145, Asp-194, Asp-212 and Phe-213, forming 8, 7, 9 and 9 non-bonding interactions of each, respectively. The Asp-212 turned out to have the maximum ΔASA (48.05 Å^2^) upon binding, followed by Met-223 (36.98 Å^2^) (Appendix A, Table A5). Of the 17 interacting residues, 13 residues besides ATP were common among the interacting residues of the native inhibitor: Asn-82, Lys-101, Leu-122, Ile-145, Asp-194, Asp-212, Phe-213, Gly-214, Val-215, Ser-216, Leu-219, Ile-220, and Met-223 (Figure 4A) (Table 1), and they share the same key residues, Asp-212 and Phe-213.

**Figure 5:**
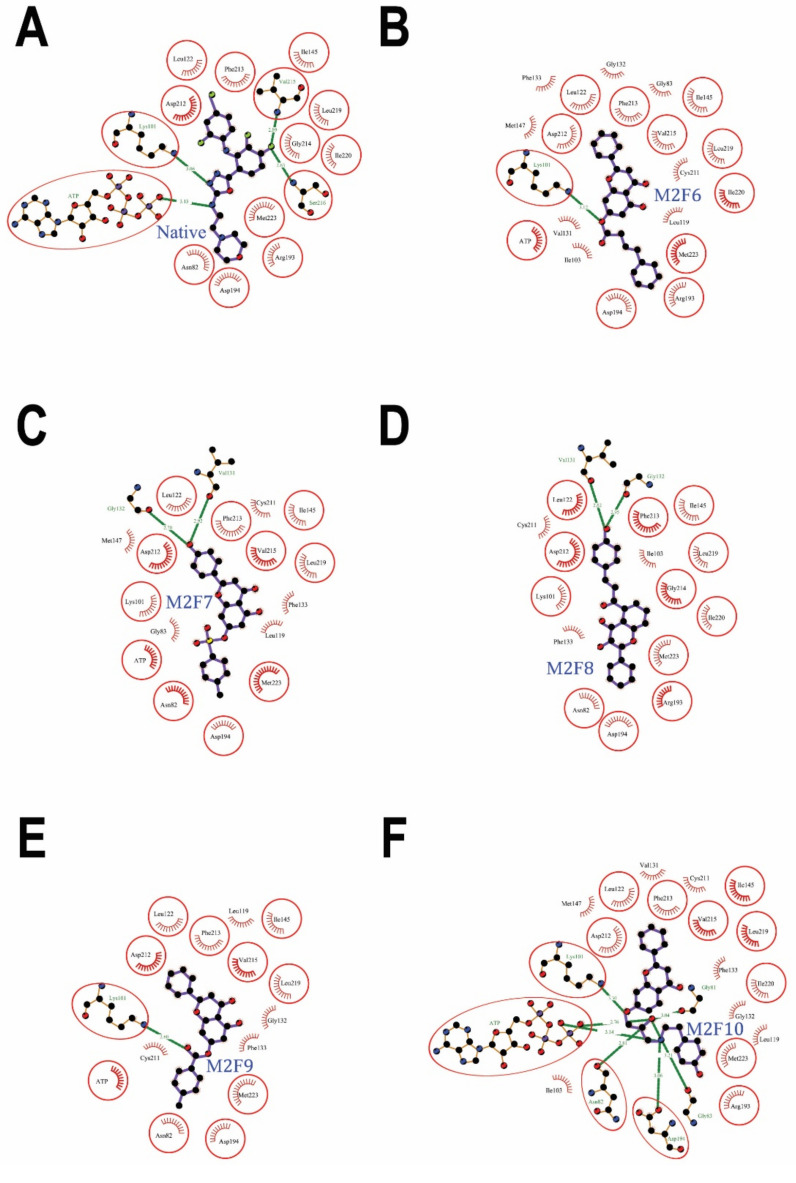
MEK2 protein-ligand interaction plots. 2. Native inhibitor (**A**) and selected flavonoids (**B**–**F**). The residues forming non-bonding interactions are shown as red bristles, while residues forming hydrogen bonds and the bound ligand are shown as ball-and-stick representations. The carbon atoms are shown as black balls, nitrogen atoms as blue balls, oxygen atoms as red balls, and sulfur atoms as yellow balls. The interacting residues common with those of the native inhibitor are shown in circles. The hydrogen bonds are shown as green dashed lines labeled with bond length (in Å).

The sixth-ranked flavonoid **M2F6** (CID: 100986463) docked well in the MEK2 allosteric site (Figure 3B). The protein-ligand interaction showed 1 hydrogen bond and 72 non-bonded contacts (mainly hydrophobic interactions) through 19 amino acids: Gly-83, Lys-101, Ile-103, Leu-119, Leu-122, Val-131, Gly-132, Phe-133, Ile-145, Met-147, Arg-193, Asp-194, Cys-211, Asp-212, Phe-213, Val-215, Leu-219, Ile-220, and Met-223 (Figure 5B) (Table 1). The docking affinity was −10.6 Kcal/mol, while the binding energy was −10.40 Kcal/mol, and the dissociation constant$,pK_{d}$, was 7.62, which demonstrated a good binding affinity, as needed for the effective inhibition of MEK2 (Table 2). The hydrogen bond with Lys-101 measures 3.12 Å. The key interacting residues are Lys-101, Ile-145, Asp-212, Phe-213 and Leu-219, forming 7, 6, 9, 6, and 6 non-bonding interactions of each, respectively. Asp-212 proved to have the maximum ΔASA with 49.47 Å^2^ upon binding, followed by Met-223 (36.55 Å^2^) (Appendix A, Table A6). Of the 19 interacting residues, 11 residues besides ATP were common among the interacting residues of the native inhibitor: Lys-101, Leu-122, Ile-145, Arg-193, Asp-194, Asp-212, Phe-213, Val-215, Leu-219, Ile-220, and Met-223 (Figure 5A) (Table 1), and they share the same key residues, Asp-212 and Phe-213. This suggests that the proposed compound was blocking the same group of residues as the native inhibitor, and hence, it may inhibit the activity of the protein in the same manner.

The dock results of the seventh-ranked flavonoid **M2F7** (CID: 102577930) against the MEK2 binding pocket fitted well in the allosteric site (Figure 3C). The ligand interacted with 17 amino acids: Asn-82, Gly-83, Lys-101, Leu-119, Leu-122, Val-131, Gly-132, Phe-133, Ile-145, Met-147, Asp-194, Cys-211, Asp-212, Phe-213, Val-215, Leu-219, and Met-223 (Figure 5C) (Table 1). The molecular docking results show a docking affinity of −10.5 Kcal/mol, a binding energy of −9.99 Kcal/mol, and a dissociation constant$,pK_{d}$, of 7.33, showing good quality binding, as required for the adequate inhibition of MEK2 (Table 2). The interaction shows 2 hydrogen bonds and 56 non-bonded contacts (mainly hydrophobic interactions). The hydrogen bonds with Val-131 measure 2.92 Å, and the hydrogen bonds with Gly-132 measure 2.70 Å. The key interacting residues are Asp-212, Phe-213, and Leu-219, forming 9, 6, and 6 non-bonding interactions of each, respectively. Asp-212 turned out to have the maximum ΔASA (49.6 Å^2^) upon binding, followed by Met-223 (41.51 Å^2^) (Appendix A, Table A7). Of the 17 interacting residues, 11 residues besides ATP were common among the interacting residues of the native inhibitor: Asn-82, Lys-101, Leu-122, Ile-145, Asp-194, Asp-212, Phe-213, Val-215, Leu-219, and Met-223 (Figure 5A) (Table 1), and they share the same key residues, Asp-212 and Phe-213.

The eighth-ranked flavonoid **M2F8** (CID: 129696793) docked to the MEK2 binding pocket and bound well to the allosteric site (Figure 3C). The ligand interacted with 17 residues: Asn-82, Lys-101, Ile-103, Leu-122, Val-131, Gly-132, Phe-133, Ile-145, Arg-193, Asp-194, Cys-211, Asp-212, Phe-213, Gly-214, Leu-219, Ile-220, and Met-223 (Figure 5D) (Table 1). The molecular docking data show a docking affinity of −10.4 Kcal/mol, a binding energy of −9.71 Kcal/mol, and a dissociation constant$,pK_{d}$, of 7.12, which was reasonably high, as required for the effective inhibition of MEK2 (Table 2). The interaction (Appendix A, Table A8) shows 2 hydrogen bonds and 57 non-bonded contacts (mainly hydrophobic interactions). The hydrogen bonds with Val-131 measure 2.62 Å, and the hydrogen bonds with Gly-132 measure 2.95 Å. The key interacting residues are Ile-145, Asp-194, and Asp-212, forming 7, 9, and 14 non-bonding interactions of each, respectively. Asp-212 turned out to have the maximum ΔASA (48.94 Å^2^) upon binding, followed by Met-223 (37.29 Å^2^), Asp-194 (34.13 Å^2^), and Asn-82 (33.47 Å^2^). Of the 17 interacting residues, 12 residues were common among the interacting residues of the native inhibitor: Asn-82, Lys-101, Leu-122, Val-131, Gly-132, Ile-145, Arg-193, Asp-194, Asp-212, Phe-213, Gly-214, Leu-219, Ile-220, and Met-223 (Figure 5A) (Table 1), and they share the same key residues, Asp-212 and Phe-213. They may therefore inhibit MEK2 kinase activity in a manner similar to the native inhibitor.

The ninth-ranked flavonoid **M2F9** (CID: 44382144) against the MEK2 binding pocket fitted well in the allosteric site (Figure 3C). The protein-ligand interaction showed 1 hydrogen bond and 52 non-bonded contacts (mainly hydrophobic interactions) through 14 amino acids: Asn-82, Lys-101, Leu-119, Leu-122, Gly-132, Phe-133, Ile-145, Asp-194, Cys-211, Asp-212, Phe-213, Val-215, Leu-219, and Met-223 (Figure 5E) (Table 1). Molecular docking results demonstrated an affinity of −10.4 Kcal/mol, a binding energy of −10.16 Kcal/mol, and a dissociation constant$,pK_{d}$, of 7.45, showing high-quality binding for the effective inhibition of MEK2 (Table 2). The hydrogen bond with Lys-101 measures 2.80 Å. The key interacting residues are Asp-212, Phe-213, and Leu-219, forming 12, 6, and 6 non-bonding interactions of each, respectively. The Asp-212 turned out to have the maximum ΔASA (50.02 Å^2^) upon binding, then Met-223 (40.9 Å^2^) (Appendix A, Table A9). Of the 14 interacting residues, 10 residues besides ATP were common among the interacting residues of the native inhibitor: Asn-82, Lys-101, Leu-122, Ile-145, Asp-194, Asp-212, Phe-213, Val-215, Leu-219, and Met-223 (Figure 5A) (Table 1), and they share the same key residues, Asp-212 and Phe-213.

The tenth-ranked flavonoid **M2F10** (CID: 14626316) bound in MEK2 binding pocket and fitted well in the allosteric site (Figure 3C). The protein-ligand complex shows 7 hydrogen bonds and 74 non-bonded contacts (mainly hydrophobic interactions) through ATP and 21 residues: Gly-81, Asn-82, Gly-83, Lys-101, Ile-103, Leu-119, Leu-122, Val-131, Gly-132, Phe-133, Ile-145, Met-147, Asp-194, Cys-211, Asp-212, Phe-213, Val-215, Leu-219, Ile-220, and Met-223 (Figure 5F) (Table 1). The interaction shows a docking affinity of −10.4 Kcal/mol, a binding energy of −10.35 Kcal/mol, and a dissociation constant$,pK_{d}$, of 7.59, which is considered good quality binding, as required for the adequate inhibition of MEK2 (Table 2). The hydrogen bonds with Gly-81 measure 3.04 Å, while the bonds with Asn-82 measure 2.81 Å, the bonds with Gly-83 measure 3.21 Å, the bonds with Lys-101 measure 3.30 Å, the bonds with Asp-194 measure 3.06 Å, and two hydrogen bonds with ATP measure 2.76 Å and 3.14 Å. The key interacting residues are Lys-101, Ile-145, Asp-212, and Phe-213, with 6 non-bonding interactions of each and with Asp-194 demonstrated 7 non-bonding interactions. Asp-212 turned out to have the maximum ΔASA (48.8 Å^2^) upon binding, followed by Met-223 (37.61 Å^2^) (Appendix A, Table A10). Of the 21 interacting residues, 12 residues besides ATP were common among the interacting residues of the native inhibitor: Asn-82, Lys-101, Leu-122, Ile-145, Arg-193, Asp-194, Asp-212, Phe-213, Val-215, Leu-219, Ile-220, and Met-223 (Figure 5A) (Table 1), and they share the same key residues, Asp-212 and Phe-213. They may therefore inhibit MEK2 kinase activity in a manner similar to the native inhibitor.

All the ten selected flavonoids showed a high docking affinity, binding energy, and dissociation constant score and are proposed as potential inhibitors of MEK2 kinase. Asp-212 was observed as a common key interacting residue, playing a role in the binding of the ten proposed flavonoids as it showed a maximum ΔASA due to binding (except for M2F3, where it was the second maximum). Additionally, another key residue, Lys-101, was found to form a hydrogen bond in seven cases out of ten. In the remaining three cases, where it did not form a hydrogen bond but appeared as an important interacting residue.

### 2.2. Common Patterns for Docking Affinity in the Flavonoids by Structure Activity Relationship (SAR)

**Figure 6:**
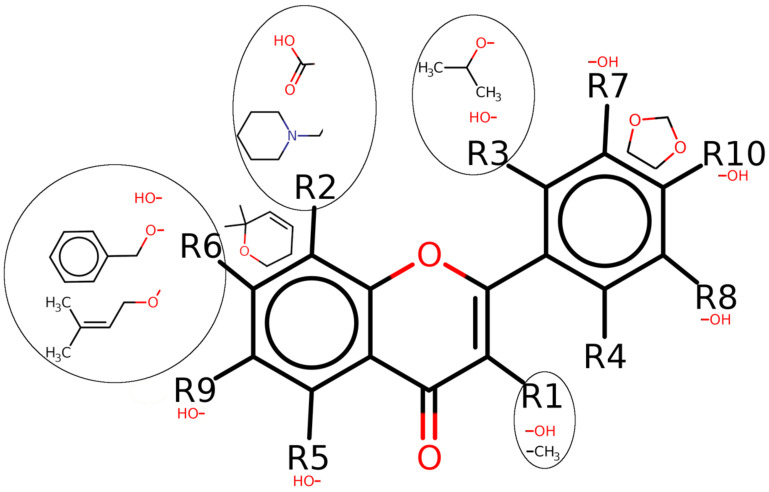
Common patterns for high docking affinity in the flavonoids by structure–activity relationship (SAR). Substitutions of the chemical groups shown at different R sites were observed with high docking affinity in a number of flavonoids. Two groups for ring fusions at the R2-R6 bond and R7-R10 bond were also found to be with high docking affinity in multiple flavonoids.

This analysis excluded 665 compounds (of a total of 1289) as the symmetric scaffolds matched multiple times and the software could not assign R sites in a unique way. In the analyzed flavonoids, the carbonyl group (C=O) in the two fused rings of the core was found to be common throughout (Figure 6). The ten R sites (R1–R10) were identified on the common core, where different chemical groups were found in the analyzed set of flavonoids. The effect of these chemical groups on different R sites was analyzed. With hydroxyl group (-OH) substitution on all R sites except for R2 and R4, the docking affinity was found to be commonly high in a number of flavonoids. No common pattern was observed for any substitution at the R4 site. At the R1 site, methyl group (-CH3) substitution (in addition to -OH substitution) was also found to have a high docking affinity in multiple flavonoids. The substitution of various other chemical groups shown as encircled with their respective R sites (R2, R3 and R6) were found to demonstrate high docking affinities in multiple flavonoids. In addition, the ring fusions of the two respective groups at the R2-R6 bond and the R7-R10 bond were found to have a high docking affinity in multiple flavonoids. However, such patterns were not observed for low docking affinity in multiple flavonoids.

### 2.3. Drug-Likeness and Pharmacokinetics Prediction

Drug design is a sequential evaluation process, and a lack of evaluation can lead to a drug being rejected, which is expensive and time-consuming for any company. Here, we studied the pharmacokinetics of the selected compounds to predict their properties in absorption, distribution, metabolism, excretion, toxicity, and drug-likeness based on the Lipinski rule [40]. The prediction of drug-likeness for the selected inhibitor for MEK2 is presented in Table 3. Considering the desired values of molecular weight <500, H-bond donors <5, H-bond acceptors <10, rotatable bonds <10, and lipophilicity (logP) <5, all the selected compounds follow the desired values, except for **M2F6,** where the lipophilicity is slightly higher than 5. All the ADMET-predicted properties of the top selected inhibitors for MEK1 are represented in Table 4. The efficacy of the selected compounds as oral medicine was determined using two models for measuring absorption properties, including CaCO2 permeability and intestinal absorption, where the desired CaCO2 permeability is >0.90 and intestinal absorption >30%. The prediction of the screened compounds shows the CaCO2 permeability values, and the following five compounds follow the desired value: **M2F2, M2F3, M2F6, M2F8** and **M2F9**. At the same time, intestinal absorption shows percentages that are all higher than 60%, which is considered good absorption, except for **M2F5,** with 26.637%. Skin permeability is the next important factor in absorption. The ideal skin permeability is >−2.5 log Kp, and all of the compounds under study have permeability values of less than −2.5 log Kp, indicating poor skin permeability. The ATP-binding cassette (ABC) transporter, which is necessary for efficient molecular transport across cell membranes, contains P-glycoprotein. P-glycoprotein substrates and inhibitors of P-glycoprotein I and II were examined in all of the substances that were screened. All of the compounds were discovered to be substrates, indicating that they can transport through the cell membrane through the ABC transporter. Additionally, **M2F5** was found to be ineffective as an inhibitor of the P-glycoprotein I and II transporter, and **M2F1** was found to be ineffective as an inhibitor of the P-glycoprotein I transporter, suggesting that they could be incapable of inhibiting these two drug efflux pumps. The distribution of the substances in the body was determined using four distinct assays: volume of distribution (VDss), fraction unbound, BBB permeability, and central nervous system (CNS) permeability.We began with the VDss assay, which is used to evaluate the total amount of a drug needed for uniform drug distribution in the bloodstream; readings less than −0.15 log are considered negative, while values greater than 0.45 log are considered good diffusion. Thus, **M2F3** shows a good distribution; **M2F10** shows average VDss values, while other compounds have low distribution volumes. The potential of a drug to reach the brain is determined by the permeability of the blood–brain barrier (BBB). If the logBB values are more than 0.3, they will cross the BBB. The logBB value of the screened compounds is less than 0.3, meaning that none of them will be able to cross the BBB. The desired value of the CNS permeability is >−2, and the screen shows good CNS permeability results for **M2F3, M2F6, M2F8,** and **M2F9**. Seven distinct cytochrome models were used to examine the test drugs’ metabolism in the body. All of the compounds were tested for their capacity to inhibit CYP1A2, CYP2C19, CYP2D6, CYP2C9, and CYP3A4, as well as their ability to function as a substrate for CYP2D6 and CYP3A4. The total clearance rates of all of the examined compounds were varied, and none of them appeared to be a substrate for organic cation transporter 2 (OCT2). They also failed to anticipate AMES toxicity, showing that these chemicals are neither carcinogenic nor mutagenic, except for **M2F1, M2F8** and **M2F10**. Only three out of the selected flavonoids were predicted to be negative for hepatotoxicity, whereas none of the substances tested positive for skin sensitization.

### 2.4. MD Simulation

**Figure 7:**
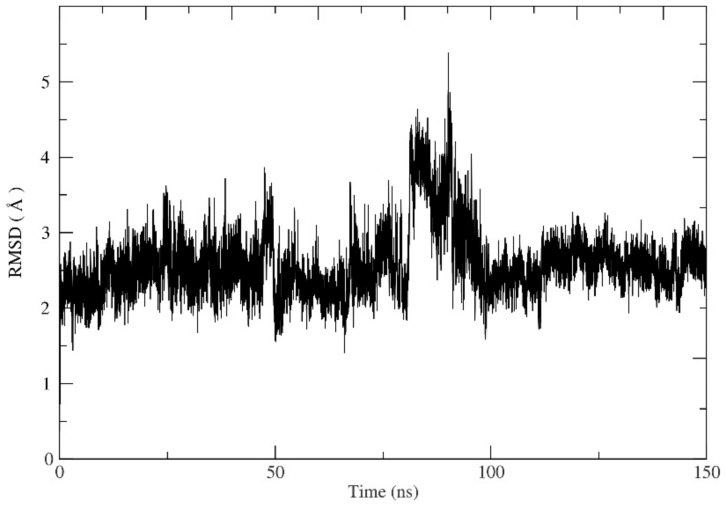
Root-mean-square deviation (RMSD) of ligand heavy atoms after least-square fitting to backbone confirmations of protein-ligand complexes during 150 ns.

**Figure 8:**
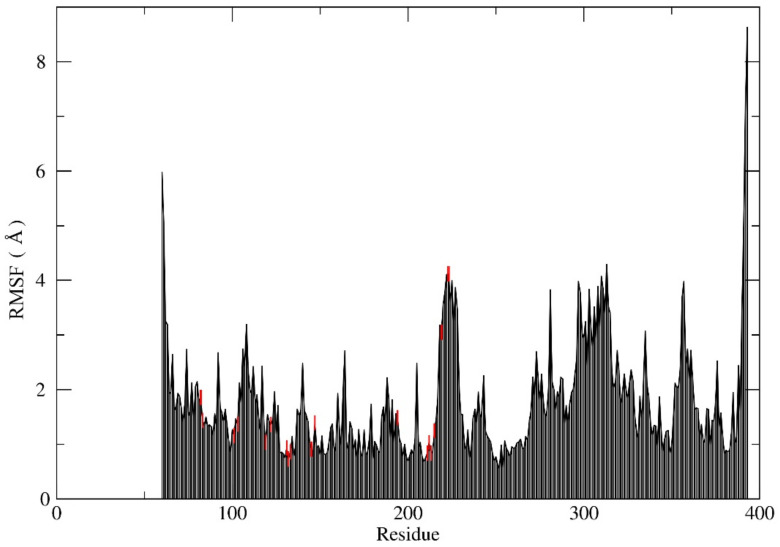
Root-mean-square fluctuations (RMSF) confirmations of protein-ligand complexes, with interacting residues in red.

**Figure 9:**
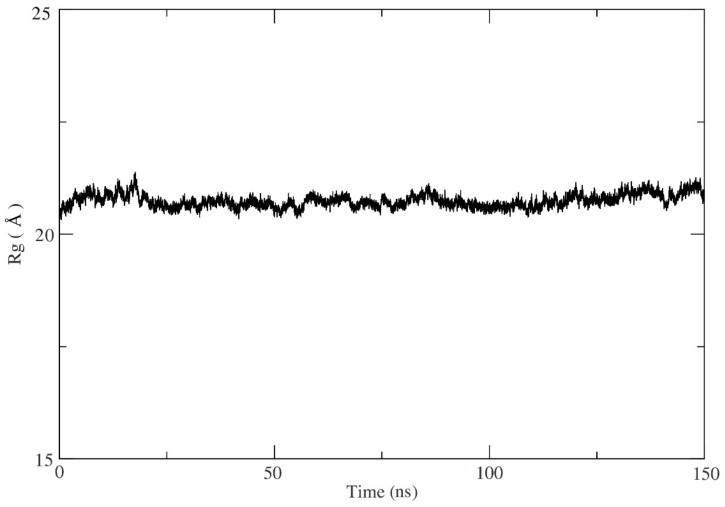
Radius of gyration of protein-ligand complexes.

**Figure 10:**
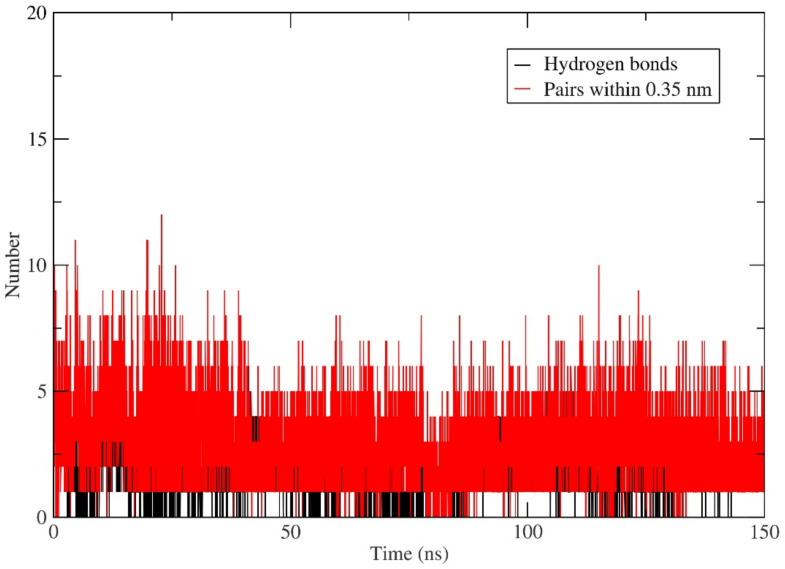
Estimation of the hydrogen bond interactions during the whole simulation.

**Figure A1:**
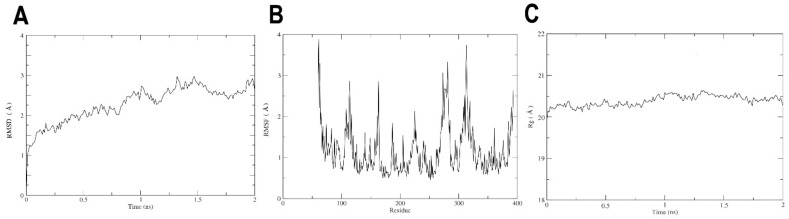
MEK2 modified protein molecular dynamic simulation for 2 (ns). (**A**): Root-mean-square deviation (RMSD) curve for the protein backbone. RMSD plot provides quantification of the overall stability of the protein backbone during 2 ns simulation. From the graph we can see the values range between 1–2.5 Å for MEK2. (**B**): Root-mean-square fluctuations (RMSF) curve of the MEK2 residues. The highest fluctuation was in the missing loop area up to 4 Å for MEK2 which is originally high flexible region and the others resides seem to be fluctuate in only around 2 Å. (**C**): Radius of gyration (total) of the protein. The graph shows the protein gyrate only around 20 Å for MEK2, moreover no big changes through the 2 ns simulation which indicate the stability of the shape of protein.

A molecular dynamics (MD) simulation was used to refine and assess the MEK2 after adding the missing loop (Appendix A, Figure A1) and explore the binding stability of the docked protein-ligand final system. The best conformations of the first-ranked flavonoid obtained from the docking proceeded to 150 (ns) MD simulations. Furthermore, the MD results were examined for root-mean-square deviation (RMSD), root-mean-square fluctuation (RMSF), the radius of gyration, and the number of hydrogen bonds to evaluate the stability of the protein-ligand complex. The RMSD is a measure of the distance the ligand deviated from its starting conformation over the course of the simulation. The RMSD values for MEK2 were found to be 2–5 Å, which is considered stable (Figure 7). The RMSD value started with 2 Å and kept a stable value between 2–3 ns, then raised to 4–5 Å after 80 ns to restabilize at 2 Å after at the end of the simulation. The RMSD deviation indicates that the equilibration of a system is achieved through the simulation. The RMSF is important for the determination of the conformation and to define the fixable area in the protein as a function of residue number. The RMSF is a metric that measures how much ligand binding affects the flexibility of MEK2 residues. The RMSF values show that the maximum atom fluctuation is up to 4 Å. From this perspective, the protein seems stable (Figure 8). The radius of gyration indicates the compactness of the protein. The lower degree of fluctuation suggests a higher degree of compactness and stability of the system. Additionally, from the radius of gyration, the protein seems notably stable. The radius merely fluctuates in a range of approximately 20 Å during the entire simulation (Figure 9). The stability of the protein-ligand complex molecule largely depends on hydrogen bonds. The analysis of hydrogen bonds is presented in Figure 10, which shows the number of hydrogen bonds during the interaction of MEK2 with the flavonoid. Looking more closely at this figure, it can be seen that the number of hydrogen bonds reaches 15 in MEK2. Because of the isolated hydrogen bonds and high average hydrogen-bond number per time frame, the hydrogen-bond network in the complexes appeared to be strong. Other interactions were thought to hold hydrogen bonds in places where they had disappeared. As a result, no notable conformational changes in the complexes were observed across the simulated time period. These findings suggested that the MD simulation trajectories for all of the complexes after equilibrium were reliable enough for future investigation.

## 3. Materials and Methods

### 3.1. Data Retrieval and Preparation

The desired 3D structure of MEK2 complexed with the native inhibitor was retrieved from the Protein Data Bank (PDB, https://www.rcsb.org/, accessed on 21 June 2022), (PDB code: 1S9I) with a resolution of 3.20 Å [8]. A proline-rich loop, which is a highly flexible region located after the catalytic site (residues from 286 to 312), was missing from the crystalized structure; thus, Modeller v10.4 [41] was used to model the proline-rich loop. The unique allosteric site was verified [8,42], and the coordinates for the grid box covering the allosteric site were prepared using AutoDockTools-1.5.6 [43]. Other preparations were made, including removing heteroatoms, deleting water, adding polar hydrogens, computing and adding charges, and finally converting the protein into a (pdbqt) file for the molecular docking, which was also done using AutoDockTools-1.5.6 software package. A drug-like library of 1289 flavonoids was prepared in our previous research [44] from PubChem (www.ncbi.nlm.nih.gov, accessed on 24 June 2022). Ligands were prepared for virtual screening using Open Babel [45] and converted to (pdbqt) after adding hydrogens and charges. MarvinSketch v.18.4 ChemAxon (http://www.chemaxon.com/products/marvin, accessed on 1 February 2023) was used to illustrate the 2-D structure of the chemical compounds.

### 3.2. Molecular Docking

Virtual screening using molecular docking was carried out by AutoDock Vina [46]. The best mode, least RMSD, and best docking affinity results were taken and ranked. The top 10 ranked compounds were named M2F1 (1st rank), M2F2 (2nd rank), ....., M2F10 (10th rank) according to their rank. For additional analysis of the docking, illustrations of the docking results were made using PyMOL v2.4.0 [47], and protein-ligand interaction illustrations of selected flavonoids with MEK2 were performed using Ligplot+ v1.4.5 [48]. Binding energy and dissociation constant calculations were performed by XScore v1.2.11 [49]. The degree of ligand filling the binding site was studied by calculating the loss in accessible surface area (ΔASA). If a residue loses more than 10 Å^2^ as a result of the binding, it is considered to have a part in the binding [50].

$$\Delta ASA=ASA^{protein}-ASA^{protein-ligand}$$

All the ASA calculations of the unbound proteins and protein-ligand complexes were performed by Naccess v.2.1.1 [51].

### 3.3. Drug-Likeness and Prediction of Pharmacokinetics 

Evaluations of the drug-likeness and pharmacokinetic properties, including the absorption, distribution, metabolism, excretion, and toxicity (ADMET), were carried out using the “pkCSM-pharmacokinetics” online freely accessible web server (http://biosig.unimelb.edu.au/pkcsm/, accessed on 12 July 2022).

### 3.4. Structure–Activity Relationship (SAR) Analysis

The structure–activity relationship (SAR) was assessed by DataWarrior v.550 [52]. For the SAR analysis, the docking affinity score was considered the activity. The option ‘core-based SAR analysis’ was selected, considering the flavone scaffold as the core. The effect of the substitution of different chemical groups at each R site on the core was analyzed independently. The common patterns observed for a number of compounds are reported.

### 3.5. Molecular Dynamics Simulation

For further evaluation of the binding stability of the best-docked ligand, a molecular dynamics (MD) simulation was carried out for 150 ns using Gromacs v.2020.5 [53]. The MD protocol used here is the same as described in our previous article [44].

## 4. Conclusions

In this in silico study, we proposed 10 flavonoids as potential inhibitors of the anticancer therapeutic target MEK2. The selected compounds have good binding affinity and ligand interactions, suggesting that they may be better inhibitors of MEK2 compared to the native one. The analyses of the flavonoids’ pharmacokinetic properties showed that the selected flavonoids might be good drug candidates. The stability of the highest-ranking flavonoid and MEK2 complex was further verified by MD simulation studies with a 150 ns time scale that measured the root-mean-square deviation, root-mean-square fluctuation, the radius of gyration, and the number of hydrogen bonds. Our results suggest that the selected flavonoids may inhibit MEK2 and present a promising targeted therapy for the treatment of cancer in the future. Future studies are required to ascertain the efficacy of the screened flavonoids in cancer therapy.

## Figures and Tables

**Table 1:** Interacting residues of MEK2 for the ten proposed compounds. Each column contains interacting residues for the compound mentioned at the top of the column. The common interacting residues for different compounds are placed in the same row, and the interacting residues in common with the native ligand are in bold font.

M2F1	M2F2	M2F3	M2F4	M2F5	M2F6	M2F7	M2F8	M2F9	M2F10
-	-	-	-	-	-	-	-	-	Gly-81
**Asn-82**	**Asn-82**	**Asn-82**	**Asn-82**	**Asn-82**	**-**	**Asn-82**	**Asn-82**	**Asn-82**	**Asn-82**
Gly-83	-	-	-	-	Gly-83	Gly-83	-	-	Gly-83
**Lys-101**	**Lys-101**	**Lys-101**	**Lys-101**	**Lys-101**	**Lys-101**	**Lys-101**	**Lys-101**	**Lys-101**	**Lys-101**
Ile-103	-	-	-	Ile-103	Ile-103	-	Ile-103	-	Ile-103
Leu-119	-	Leu-119	-	Leu-119	Leu-119	Leu-119	-	Leu-119	Leu-119
**Leu-122**	**Leu-122**	**Leu-122**	**-**	**Leu-122**	**Leu-122**	**Leu-122**	**Leu-122**	**Leu-122**	**Leu-122**
Val-131	-	-	-	-	Val-131	Val-131	Val-131	-	Val-131
Gly-132	-	-	-	-	Gly-132	Gly-132	Gly-132	Gly-132	Gly-132
Phe-133	-	-	-	-	Phe-133	Phe-133	Phe-133	Phe-133	Phe-133
**Ile-145**	**Ile-145**	**Ile-145**	**Ile-145**	**Ile-145**	**Ile-145**	**Ile-145**	**Ile-145**	**Ile-145**	**Ile-145**
Met-147	Met-147	-	-	Met-147	Met-147	Met-147	-	-	Met-147
-	**Arg-193**	**Arg-193**	**Arg-193**	**-**	**Arg-193**	**-**	**Arg-193**	**-**	**Arg-193**
**Asp-194**	**Asp-194**	**Asp-194**	**Asp-194**	**Asp-194**	**Asp-194**	**Asp-194**	**Asp-194**	**Asp-194**	**Asp-194**
Cys-211	Cys-211	-	Cys-211	Cys-211	Cys-211	Cys-211	Cys-211	Cys-211	Cys-211
**Asp-212**	**Asp-212**	**Asp-212**	**Asp-212**	**Asp-212**	**Asp-212**	**Asp-212**	**Asp-212**	**Asp-212**	**Asp-212**
**Phe-213**	**Phe-213**	**Phe-213**	**Phe-213**	**Phe-213**	**Phe-213**	**Phe-213**	**Phe-213**	**Phe-213**	**Phe-213**
**-**	**Gly-214**	**Gly-214**	**Gly-214**	**Gly-214**	**-**	**-**	**Gly-214**	**-**	**-**
**Val-215**	**Val-215**	**Val-215**	**Val-215**	**Val-215**	**Val-215**	**Val-215**	**-**	**Val-215**	**Val-215**
-	-	**Ser-216**	**-**	**Ser-216**	**-**	**-**	**-**	**-**	**-**
**Leu-219**	-	**Leu-219**	**-**	**Leu-219**	**Leu-219**	**Leu-219**	**Leu-219**	**Leu-219**	**Leu-219**
-	-	**Ile-220**	**-**	**Ile-220**	**Ile-220**	**-**	**Ile-220**	**-**	**Ile-220**
**Met-223**	**Met-223**	**Met-223**	**Met-223**	**Met-223**	**Met-223**	**Met-223**	**Met-223**	**Met-223**	**Met-223**
-	-	-	Met-234	-	-	-	-	-	-

**Table 2:** The selected flavonoids and the binding strength score against MEK2 (Autodock Vina docking energy, X-score binding energy, and $pK_{d}$). The higher the absolute values of the scores, the better the binding.

Rank	Flavonoids	Docking Affinity (Kcal/mol)	Binding Energy (Kcal/mol)	$pK_{d}$ $\;\mathrm{or}\;-\log(K_{d})$
1	M2F1	−11.3	−10.09	7.40
2	M2F2	−11.1	−9.41	6.90
3	M2F3	−11.1	−10.38	7.61
4	M2F4	−10.9	−9.26	6.79
5	M2F5	−10.9	−10.02	7.34
6	M2F6	−10.6	−10.40	7.62
7	M2F7	−10.5	−9.99	7.33
8	M2F8	−10.4	−9.71	7.12
9	M2F9	−10.4	−10.16	7.45
10	M2F10	−10.4	−10.35	7.59
Native		−11.0	−9.49	6.96

**Table 3:** Structures and chemical properties of the selected flavonoids against MEK2.

	Compound	Molecular Weight	LogP	#Rotatable Bonds	#Acceptors	#Donors	Surface Area
**1**	**M2F1**	390.347	3.5134	3	7	4	162.897
**2**	**M2F2**	378.355	4.5893	4	5	2	158.426
**3**	**M2F3**	406.478	4.7624	3	5	2	175.819
**4**	**M2F4**	394.379	2.7771	3	7	5	165.246
**5**	**M2F5**	414.366	0.731	4	8	4	168.886
**6**	**M2F6**	398.414	5.1745	5	5	1	172.033
**7**	**M2F7**	424.43	3.94732	4	7	2	171.095
**8**	**M2F8**	384.387	4.7673	4	5	2	165.349
**9**	**M2F9**	372.376	4.69322	3	5	1	159.993
**10**	**M2F10**	431.488	3.7376	9	6	3	185.27

**Table 4:** ADMET properties of the selected flavonoids against MEK2.

Property	Model Name	Predicted Value										Unit
		M2F1	M2F2	M2F3	M2F4	M2F5	M2F6	M2F7	M2F8	M2F9	M2F10	
**Absorption**	Water solubility	−3.096	−4.254	−4.26	−3.603	−3.243	−5.17	−4.523	−4.456	−4.439	−4.611	Numeric (log mol/L)
	CaCO2 permeability	−0.504	1.075	1.125	0.233	0.502	0.916	0.455	1.089	0.954	0.15	Numeric (log Papp in 10^−6^ cm/s)
	Intestinal absorption (human)	84.712	93.56	95.486	68.737	26.637	94.261	95.63	90.869	96.44	90.988	Numeric (% Absorbed)
	Skin permeability	−2.735	−2.735	−2.86	−2.735	−2.735	−2.736	−2.735	−2.735	−2.737	−2.729	Numeric (log Kp)
	P-glycoprotein substrate	Yes	Yes	Yes	Yes	Yes	Yes	Yes	Yes	Yes	Yes	Categorical (Yes/No)
	P-glycoprotein I inhibitor	No	Yes	Yes	Yes	No	Yes	Yes	Yes	Yes	Yes	Categorical (Yes/No)
	P-glycoprotein II inhibitor	Yes	Yes	Yes	Yes	No	Yes	Yes	Yes	Yes	Yes	Categorical (Yes/No)
**Distribution**	VDss (human)	−1.217	−0.908	0.553	−0.876	−1.01	−0.683	−0.958	−0.696	−0.408	0.312	Numeric (log L/kg)
	Fraction unbound (human)	0.02	0.045	0	0	0.143	0.184	0.043	0.039	0.171	0.04	Numeric (Fu)
	BBB permeability	−1.566	−0.034	0.181	−1.2	−1.202	−0.044	−0.613	−0.371	−0.174	−0.81	Numeric (log BB)
	CNS permeability	−3.067	−2.136	−1.86	−3.252	−4.053	−1.768	−3.352	−1.883	−1.844	−2.563	Numeric (log PS)
**Metabolism**	CYP2D6 substrate	No	No	No	No	No	No	No	No	No	No	Categorical (Yes/No)
	CYP3A4 substrate	No	Yes	Yes	Yes	No	Yes	Yes	Yes	Yes	Yes	Categorical (Yes/No)
	CYP1A2 inhibitior	Yes	Yes	No	No	No	Yes	Yes	Yes	Yes	Yes	Categorical (Yes/No)
	CYP2C19 inhibitior	Yes	Yes	Yes	No	No	Yes	Yes	Yes	Yes	Yes	Categorical (Yes/No)
	CYP2C9 inhibitior	Yes	Yes	Yes	Yes	No	Yes	Yes	Yes	Yes	Yes	Categorical (Yes/No)
	CYP2D6 inhibitior	No	No	No	No	No	No	No	No	No	Yes	Categorical (Yes/No)
	CYP3A4 inhibitor	No	Yes	Yes	Yes	No	Yes	No	Yes	Yes	Yes	Categorical (Yes/No)
**Excretion**	Total clearance	0.006	0.268	0.523	0.057	0.321	0.595	0.548	0.184	0.808	0.87	Numeric (log ml/min/kg)
	Renal OCT2 substrate	No	No	No	No	No	No	No	No	No	Yes	Categorical (Yes/No)
**Toxicity**	AMES toxicity	Yes	No	No	No	No	No	No	Yes	No	Yes	Categorical (Yes/No)
	Max. tolerated dose (human)	0.491	0.362	−0.296	0.394	0.556	0.55	0.26	0.204	0.592	0.088	Numeric (log mg/kg/day)
	hERG I inhibitor	No	No	No	No	No	No	No	No	No	No	Categorical (Yes/No)
	hERG II inhibitor	Yes	Yes	Yes	Yes	No	Yes	Yes	Yes	Yes	Yes	Categorical (Yes/No)
	Oral rat acute toxicity (LD50)	2.821	2.849	1.874	2.395	2.668	2.947	2.512	2.767	3.218	2.679	Numeric (mol/kg)
	Oral rat chronic toxicity (LOAEL)	2.722	1.566	2.031	1.992	3.522	1.014	2.14	0.914	1.232	3.299	Numeric (log mg/kg_bw/day)
	Hepatotoxicity	No	Yes	No	No	Yes	Yes	Yes	Yes	Yes	Yes	Categorical (Yes/No)
	Skin sensitisation	No	No	No	No	No	No	No	No	No	No	Categorical (Yes/No)
	* T.Pyriformis * toxicity	0.285	0.287	0.433	0.287	0.285	0.289	0.285	0.29	0.29	0.287	Numeric (log ug/L)
	Minnow toxicity	1.329	−0.496	0.312	2.214	−0.801	−3.608	−2.455	0.09	−2.328	−2.547	Numeric (log mM)

## Data Availability

PDB, https://www.rcsb.org/, accessed on 21 June 2022; www.ncbi.nlm.nih.gov, accessed on 24 June 2022.

## Supplementary Materials

The supporting information can be downloaded at: https://www.mdpi.com/article/10.3390/ijms24054446/s1.

## Appendix A

**Table A1:** The MEK2 residues interacting with selected flavonoid M2F1 are listed with the number of non-bonding interactions and ΔASA.

Interacting Residues	Hydrogen Bonds	Non-Bonding Interactions	$\Delta ASA\;(\mathrm{in}\;{Å}^{2})$
Asn-82	0	2	19.66
Gly-83	0	1	3.33
Lys-101	0	3	17.74
Ile-103	0	2	6.64
Leu-119	0	1	1.85
Leu-122	0	5	12.81
Val-131	0	2	4.12
Gly-132	0	2	1.47
Phe-133	0	2	1.5
Ile-145	0	6	23.51
Met-147	0	2	10
Asp-194	1	2	22.63
Cys-211	0	3	4.96
Asp-212	2	6	48.59
Phe-213	0	7	13.86
Val-215	0	5	7.53
Leu-219	0	6	14.31
Met-223	0	6	29.83
ATP	1	0	

**Table A2:** The MEK2 residues interacting with selected flavonoid M2F2 are listed with the number of non-bonding interactions and ΔASA.

Interacting Residues	Hydrogen Bonds	Non-Bonding Interactions	$\Delta ASA\;(\mathrm{in}\;{Å}^{2})$
Asn-82	0	4	38.46
Lys-101	1	2	16.97
Leu-122	0	2	12.81
Ile-145	0	4	23.51
Met-147	0	2	10
Arg-193	0	2	20.38
Asp-194	0	11	35.75
Cys-211	0	1	4.96
Asp-212	0	17	48.52
Phe-213	0	6	13.86
Gly-214	0	1	7.53
Val-215	0	2	7.53
Met-223	0	2	36.8

**Table A3:** The MEK2 residues interacting with selected flavonoid M2F3 are listed with the number of non-bonding interactions and ΔASA.

Interacting Residues	Hydrogen Bonds	Non-Bonding Interactions	$\Delta ASA\;(\mathrm{in}\;{Å}^{2})$
Asn-82	0	3	38.86
Lys-101	1	0	17.92
Leu-119	0	2	1.85
Leu-122	0	2	11.31
Ile-145	0	2	23.28
Arg-193	0	1	26.74
Asp-194	0	3	34.51
Asp-212	0	8	48.86
Phe-213	0	4	13.1
Gly-214	0	1	6.74
Val-215	1	6	7.53
Ser-216	1	4	1.36
Leu-219	0	5	14.31
Ile-220	0	2	22.67
Met-223	0	5	51.44

**Table A4:** The MEK2 residues interacting with selected flavonoid M2F4 are listed with the number of non-bonding interactions and ΔASA.

Interacting Residues	Hydrogen Bonds	Non-Bonding Interactions	$\Delta ASA\;(\mathrm{in}\;{Å}^{2})$
Asn-82	0	4	40.58
Lys-101	1	3	17.36
Ile-145	0	3	23.48
Arg-193	0	2	22.48
Asp-194	0	9	38.11
Cys-211	0	2	4.96
Asp-212	0	18	48.86
Phe-213	0	9	13.86
Gly-214	0	1	7.4
Val-215	0	1	7.53
Met-223	0	3	37.38
Met-234	0	1	23.45
ATP	2	0	

**Table A5:** The MEK2 residues interacting with selected flavonoid M2F5 are listed with the number of non-bonding interactions and ΔASA.

Interacting Residues	Hydrogen Bonds	Non-Bonding Interactions	$\Delta ASA\;(\mathrm{in}\;{Å}^{2})$
Asn-82	2	5	28.18
Lys-101	1	3	17.86
Ile-103	0	1	6.64
Leu-119	0	1	1.85
Leu-122	0	2	12.81
Ile-145	0	8	23.51
Met-147	0	4	10
Asp-194	0	7	27.97
Cys-211	0	1	4.96
Asp-212	0	9	48.05
Phe-213	0	9	13.86
Gly-214	0	2	7.84
Val-215	0	4	7.53
Ser-216	0	3	1.36
Leu-219	0	4	14.31
Ile-220	0	2	11.16
Met-223	0	4	36.98
ATP	1	0	

**Table A6:** The MEK2 residues interacting with selected flavonoid M2F6 are listed with the number of non-bonding interactions and ΔASA.

Interacting Residues	Hydrogen Bonds	Non-Bonding Interactions	$\Delta ASA\;(\mathrm{in}\;{Å}^{2})$
Gly-83	0	1	3.33
Lys-101	1	7	17.45
Ile-103	0	1	6.64
Leu-119	0	1	1.85
Leu-122	0	5	12.81
Val-131	0	2	4.12
Gly-132	0	2	1.47
Phe-133	0	2	1.5
Ile-145	0	6	23.51
Met-147	0	1	10
Arg-193	0	4	22.37
Asp-194	0	5	30.41
Cys-211	0	4	4.96
Asp-212	0	9	49.47
Phe-213	0	6	13.82
Val-215	0	3	7.53
Leu-219	0	6	14.31
Ile-220	0	2	18.83
Met-223	0	4	36.55

**Table A7:** The MEK2 residues interacting with selected flavonoid M2F7 are listed with the number of non-bonding interactions and ΔASA.

Interacting Residues	Hydrogen Bonds	Non-Bonding Interactions	$\Delta ASA\;(\mathrm{in}\;{Å}^{2})$
Asn-82	0	4	29.73
Gly-83	0	1	3.33
Lys-101	0	2	18.41
Leu-119	0	1	1.85
Leu-122	0	3	12.81
Val-131	1	1	4.12
Gly-132	1	1	1.47
Phe-133	0	1	1.5
Ile-145	0	4	23.47
Met-147	0	1	10
Asp-194	0	4	26.74
Cys-211	0	3	4.96
Asp-212	0	9	49.6
Phe-213	0	6	13.86
Val-215	0	3	7.53
Leu-219	0	6	14.31
Met-223	0	5	41.51

**Table A8:** The MEK2 residues interacting with selected flavonoid M2F8 are listed with the number of non-bonding interactions and ΔASA.

Interacting Residues	Hydrogen Bonds	Non-Bonding Interactions	$\Delta ASA\;(\mathrm{in}\;{Å}^{2})$
Asn-82	0	1	33.47
Lys-101	0	1	14.5
Ile-103	0	1	6.64
Leu-122	0	4	12.81
Val-131	1	2	4.12
Gly-132	1	1	1.47
Phe-133	0	1	1.5
Ile-145	0	7	23.51
Arg-193	0	1	20.19
Asp-194	0	9	34.13
Cys-211	0	2	4.96
Asp-212	0	14	48.94
Phe-213	0	5	13.86
Gly-214	0	2	7.93
Leu-219	0	1	14.31
Ile-220	0	2	13.89
Met-223	0	3	37.29

**Table A9:** The MEK2 residues interacting with selected flavonoid M2F9 are listed with the number of non-bonding interactions and ΔASA.

Interacting Residues	Hydrogen Bonds	Non-Bonding Interactions	$\Delta ASA\;(\mathrm{in}\;{Å}^{2})$
Asn-82	0	2	29.88
Lys-101	1	2	18.75
Leu-119	0	1	1.85
Leu-122	0	3	12.81
Gly-132	0	1	1.47
Phe-133	0	1	1.5
Ile-145	0	3	23.44
Asp-194	0	4	28.39
Cys-211	0	3	4.96
Asp-212	0	12	50.02
Phe-213	0	6	13.86
Val-215	0	3	7.53
Leu-219	0	6	14.31
Met-223	0	4	40.9

**Table A10:** The MEK2 residues interacting with selected flavonoid M2F10 are listed with the number of non-bonding interactions and ΔASA.

Interacting Residues	Hydrogen Bonds	Non-Bonding Interactions	$\Delta ASA\;(\mathrm{in}\;{Å}^{2})$
Gly-81	1	0	2.56
Asn-82	1	3	27.93
Gly-83	1	3	3.33
Lys-101	1	6	17.97
Ile-103	0	3	6.64
Leu-119	0	1	1.85
Leu-122	0	5	12.81
Val-131	0	2	4.12
Gly-132	0	2	1.47
Phe-133	0	2	1.5
Ile-145	0	6	23.51
Met-147	0	1	10
Arg-193	0	5	28.3
Asp-194	1	7	33.3
Cys-211	0	4	4.96
Asp-212	0	6	48.8
Phe-213	0	6	13.68
Val-215	0	3	7.53
Leu-219	0	2	13.88
Ile-220	0	2	19.9
Met-223	0	3	37.61
ATP	2	0	

.
