# Supplementary material for: Investigating a Library of Flavonoids as Potential Inhibitors of a Cancer Therapeutic Target MEK2 Using in Silico Methods
Source: Int J Mol Sci. 2023 Feb 23;24(5):4446. doi: 10.3390/ijms24054446 (PMC10002492; doi:10.3390/ijms24054446)
Supplement: Supplementary file 1 [file ijms-24-04446-s001.zip › ijms-2201308-supplementary.pdf]

Table S1. Flavonoids docking affinity scores for MEK2 binding pocket.

**Compound CID**	**SMILES**	**Docking affinity (kcal/mol)**
129703940	o1c(c(c(=O)c2c(cccc12)C(=O)c1cc(c(c(c1)O)O)O)O)c1ccccc1	-11.3
131751372	O1c2c3C=CC(C)(C)Oc3ccc2C(=O)[C@H]([C@@H]1c1ccc(c(c1)CC=C(C)C)O)O	-11.1
56649181	c1(c(cc2c(c(=O)cc(o2)c2ccccc2)c1O)O)OCc1ccc(F)cc1	-11.1
154699552	O([C@@H]1O[C@@H]([C@H]([C@@H]([C@H]1O)O)O)C(=O)O)c1cccc(c1)c1cc(=O)c2ccccc2o1	-10.9
154699598	O([C@@H]1O[C@@H]([C@H]([C@@H]([C@H]1O)O)O)C(=O)O)c1ccccc1c1cc(=O)c2ccccc2o1	-10.9
44559902	O1c2cc(c(c(c2C(=O)[C@@H]([C@H]1c1ccc(cc1)O)O)O)O)Cc1ccc(cc1)O)O	-10.9
101685222	O1c2cc(c(c(c2C(=O)[C@H]([C@@H]1c1ccc(cc1)O)O)O)O)Cc1ccc(cc1)O)O	-10.8
11314387	o1c(cc(=O)c2c(c(c(cc12)O)OCc1ccccc1)O)c1ccccc1	-10.8
100986463	o1c(cc(=O)c2c(cc(cc12)OC(=O)C/C=C/c1ccccc1)O)c1ccccc1	-10.6
100986464	o1c(cc(=O)c2c(cc(cc12)OC(=O)C/C=C/c1ccc(cc1)OC)O)c1ccccc1	-10.5
102577930	c1(cc(c2c(=O)cc(oc2c1)c1ccc(cc1)O)O)OS(O)(O)c1ccc(C)cc1	-10.5
12019529	o1c(cc(=O)c2c(cc(cc12)OC(=O)Cc1ccccc1)O)c1ccccc1	-10.4
129696793	o1c(c(c(=O)c2c(cccc12)C(=O)/C=C/c1ccc(cc1)O)O)c1ccccc1	-10.4
14304997	C(=O)(NCCCCN1CCN(CC1)c1ccccn1)c1c(=O)c2c(oc1c1ccccc1)cccc2	-10.4

14626316	O(c1ccc2c(=O)cc(oc2c1)c1ccccc1)C[C@H](O)CNCCc1ccc(cc1)O	-10.4
44382144	o1c(cc(=O)c2c(cc(cc12)OC(=O)c1ccc(C)cc1)O)c1ccccc1	-10.4
10575055	c1(ccc(cc1)OCc1ccc2ccccc2n1)c1oc2c(c(=O)c1O)cccc2C(=O)O	-10.3
14016776	c1(cc(c2c(=O)cc(oc2c1)c1ccc(cc1)O)O)OS(=O)(=O)O	-10.3
44382145	c1(cc(c2c(=O)cc(oc2c1)c1ccccc1)O)OC(=O)c1ccc(F)cc1	-10.3
73265448	o1cc(c(=O)c2c(cc(cc12)OCc1ccccc1)O)c1ccc(cc1)N(=O)=O	-10.3
100941751	O1c2cc(c(c(c2C(=O)C[C@H]1c1ccc(c(c1)O)OC)O)c1ccc(cc1)OC)O	-10.2
11395168	o1c(cc(=O)c2c(cc(cc12)OCc1ccccc1)O)c1ccc(c(c1)O)OC	-10.2
14408549	C(Oc1ccc2c(=O)cc(oc2c1)c1ccccc1)[C@H](O)CNCCc1ccc(c(c1)OC)OC	-10.2
25070110	O1c2cc(cc(c2C(=O)C[C@H]1c1ccc(cc1)O)O)OCc1ccccc1	-10.2
5471244	c1(cc(c2c(=O)cc(oc2c1)c1ccccc1)O)OS(=O)(=O)O	-10.2
69567244	o1c(cc(=O)c2c(cc(cc12)OCc1ccccc1)O)c1ccc(cc1)O	-10.2
85823044	o1c(cc(=O)c2c(cccc12)OCc1ccccc1)c1ccc(cc1)C=O	-10.2
10813589	O(c1ccc2c(=O)cc(oc2c1)c1ccccc1)C[C@@]1(OC(=O)C(=C)C1)c1ccc(cc1)OC	-10.1
12820520	O1c2c(cc3c(=O)c(c(oc3c2C=CC1(C)C)c1ccc2c(OCO2)c1)OC)OC	-10.1
129853633	o1c(c(c(=O)c2c(cccc12)C(=O)/C=C/c1ccc(c(c1)O)O)O)c1ccccc1	-10.1
14735885	c1(cc(=O)c2ccccc2o1)c1ccc(cc1)C(F)(F)F	-10.1
180439	o1c(cc(=O)c2cc(c(cc12)O)CC=C(C)C)c1ccc2c(OCO2)c1	-10.1
19835337	O(c1ccc2c(=O)cc(oc2c1)c1ccccc1)C[C@H](O)CNCCCCc1cnccc1	-10.1
42608050	O1C(C)(C)C=Cc2c3c(C=CC(C)(C)O3)cc(c12)[C@@H]1CC(=O)c2c(cc(cc2O1)O)O	-10.1
10991656	O1c2ccc3c(=O)cc(oc3c2C=CC1(C)C)c1ccc2c(OCO2)c1	-10.0
11582389	c1(ccc2c(=O)cc(oc2c1)c1ccccc1)O[C@@H]1O[C@@H]([C@H]([C@@H]([C@H]1O)O)OC)CO	-10.0
11790	o1c(cc(=O)c2ccc3ccccc3c12)c1ccccc1	-10.0
14626328	c1(ccc2c(=O)cc(oc2c1)c1ccccc1)OC[C@H](O)CCCO[C@H]1OCCCC1	-10.0

1522807	o1cc(c(=O)c2ccc(cc12)OCc1ccc(cc1)N(=O)=O)c1ccccc1	-10.0
563997	o1cc(c(=O)c2ccc(cc12)OCc1ccccc1)c1ccccc1	-10.0
56589538	c1(c(c(c2c(=O)cc(oc2c1)c1ccccc1)O)O)OCc1ccc(F)cc1	-10.0
67984060	o1c(c(c(=O)c2c(cc(cc12)OCc1ccccc1)O)O)c1ccc(c(c1)O)O	-10.0
100941748	O1c2cc(c(c(c2C(=O)C[C@H]1c1ccc(cc1)O)O)c1ccc(cc1)OC)O	-9.9
100941749	O1c2cc(c(c(c2C(=O)C[C@H]1c1ccc(c(c1)O)OC)O)c1ccccc1)O	-9.9
10338671	c1(ccc2c(=O)cc(oc2c1)c1ccccc1)OC[C@]1(OC(=O)C(=C)C1)C	-9.9
10981007	O1c2c(ccc3OC(C)(C)C=Cc23)C(=O)C[C@H]1c1ccc2c(OCO2)c1	-9.9
11247668	O1c2cc(c(c(c2C(=O)C[C@H]1c1ccc(c(c1)O)O)O)C/C=C(\C)/CC[C@@H](C(=C)C)O)O	-9.9
11358607	O1c2ccc3c(=O)cc(oc3c2C=CC1(C)C)c1ccccc1	-9.9
11796528	c1(ccc(cc1)OCc1ccc2ccccc2n1)c1oc2c(c(=O)c1)cccc2C(=O)O	-9.9
129834514	O1c2cc(cc(c2C(=O)C[C@H]1c1ccc(cc1)OC(=O)C)O)OCc1ccccc1	-9.9
154699985	O(c1ccc2C(=O)C[C@H](Oc2c1)c1ccccc1)[C@@H]1O[C@@H]([C@H]([C@@H]([C@H]1O)O)O)C(=O)O	-9.9
1760386	o1cc(c(=O)c2ccc(cc12)OCc1ccccc1)c1ccc(cc1)OC	-9.9
3070243	c1(c(C)c(=O)c2ccc(c(c2o1)CN1CCCCC1)OC)c1ccc(F)cc1	-9.9
3085261	c1(ccc2c(=O)c(coc2c1)c1ccc2c(OCO2)c1)O[C@@H]1O[C@@H]([C@H]([C@@H]([C@H]1O)O)O)CO	-9.9
52951514	c1(ccc(c(c1)OC)OC[C@H](C(C)(C)O)O)c1c(=O)c2c(cc3OC(C=Cc3c2)(C)C)oc1	-9.9
5469928	o1c(cc(=O)c2c(cc(c(c12)[C@H]1CCCCN1)O)O)c1ccccc1	-9.9
563887	c1(ccc(c(c1)OC)OCc1ccccc1)c1oc2ccccc2c(=O)c1OC	-9.9
101440521	o1c2cc(cc(c2c(=O)c(c1c1ccc(c(c1)C[C@@H](C(=C)C)O)O)O)O)O	-9.8
101999902	c1(coc2cc(c(c(c2c1=O)O)CCC(C)(C)O)O)c1ccc2OC(C=Cc2c1O)(C)C	-9.8

15108704	o1c(cc(=O)c2c(c(c(cc12)OCc1ccccc1)O)O)c1ccc(c(c1)O)O	-9.8
15342843	o1c(c(c(=O)c2c(cc(cc12)OC(=O)c1ccccc1)O)OC)c1ccccc1	-9.8
15383190	O1c2cc(c3c(=O)cc(oc3c2C=CC1(C)C)c1ccccc1)O	-9.8
44257869	O1c2cc3c(c(=O)cc(o3)c3ccc(cc3)O)c(c2C[C@H](C1(C)C)O)O	-9.8
49736	o1c(c(C)c(=O)c2ccc(c(c12)CN1CCCCC1)OC)c1ccccc1	-9.8
5315125	o1c2cc(cc(c2c(=O)cc1c1cc(c(c(c1)CC=C(C)C)O)O)O)O	-9.8
100943380	c1(cccc(c1)c1cc(=O)c2ccccc2o1)S(=O)(=O)Nc1ccc(C)cc1	-9.7
102321611	o1cc(c(=O)c2c(cc(cc12)[C@@H]1C=C([C@H]2C=CCOC2=C1)O)O)c1ccc(c(c1OC)OC)O	-9.7
10291777	O1c2ccc3C(=O)C[C@H](Oc3c2C[C@H]1C(C)(C)O)c1ccc(cc1O)O	-9.7
11597157	c1(ccc2c(=O)cc(oc2c1)c1ccc(cc1)O)O[C@@H]1O[C@@H]([C@H]([C@@H]([C@H]1O)O)OC)CO	-9.7
12019528	o1c(cc(=O)c2c(cc(cc12)OC(=O)c1ccccc1OC(=O)C)O)c1ccccc1	-9.7
129791328	c1(ccc2c(=O)c(c(oc2c1)C(=O)O)c1ccccc1)OCc1sc2ccccc2n1	-9.7
129825556	o1c2cc(c(c(c2c(=O)c(c1c1ccc(c(c1)/C=C/C(=C)C)O)OC)O)OC)O	-9.7
15108702	o1c(cc(=O)c2c(c(c(cc12)OCc1ccccc1)O)O)c1ccccc1	-9.7
24861960	O1c2cc(c(c(c2C(=O)C[C@H]1c1ccc(c(c1)OC)O)O)C/C=C(\C)/C/C=C/C(C)(C)O)O	-9.7
24866261	c1(cc(c2c(=O)cc(oc2c1)c1ccccc1)O)O[C@@H]1O[C@@H]([C@H]([C@@H]([C@H]1O)O)OC)CO	-9.7
3070242	c1(c(C)c(=O)c2ccc(c(c2o1)CN1CCCC1)OC)c1ccc(F)cc1	-9.7
440195	c1(ccc2C(=O)C[C@H](Oc2c1)c1ccccc1)O[C@@H]1O[C@@H]([C@H]([C@@H]([C@H]1O)O)O)CO	-9.7
44259047	c1(c(cc2c(c(=O)c(c(o2)c2ccc(cc2)O)O)c1O)O)CCC(C)(C)O	-9.7
480763	O1c2cc(c(c(c2C(=O)C[C@@H]1c1ccccc1)O)CC=C(C)C)O	-9.7
49700	c1(c(ccc2c(=O)c(C)c(oc12)c1ccccc1)OC)CN1CCOCC1	-9.7
49788613	o1c(c(c(=O)c2c(cc(c(c12)OC)OCC=C(C)C)O)O)c1ccc2c(OCO2)c1	-9.7

629905	c1(ccc(c(c1)OCc1ccccc1)OC)c1oc2cc(ccc2c(=O)c1OC)OC	-9.7
74333987	O1c2cc(c(c(c2C(=O)[C@H]([C@H]1c1cc(c(c(c1)OC)O)O)O)O)CC=C(C)C)O	-9.7
100941747	O1c2cc(c(c(c2C(=O)C[C@H]1c1ccc(cc1)O)O)c1ccccc1)O	-9.6
11222065	o1c2ccccc2c(=O)c(c1c1ccccc1)[C@@H](c1ccc(cc1)OC)O	-9.6
129834539	O1c2cc(cc(c2C(=O)C[C@H]1c1ccc(cc1)O)OC)OCc1ccccc1	-9.6
14502737	O1c2cc(c3c(=O)c(coc3c2C=CC1(C)C)c1ccc2c(OCO2)c1)O	-9.6
15491286	o1c(c(c(=O)c2c(c(c(c12)O)OC)CC=C(C)C)O)O)c1ccc(c(c1)OC)O	-9.6
399491	o1c(cc(=O)c2c(c(c(cc12)O)CC=C(C)C)O)c1ccc(cc1O)O	-9.6
44342639	c1(c(cc(c2c(=O)cc(oc12)c1ccccc1)O)OC)C(F)(F)F	-9.6
69941512	c1(ccc2c(c(=O)cc(o2)c2ccccc2)c1)OCC[C@@H]1OC(=O)C(=C1O)O	-9.6
90300755	o1c(c(c(=O)c2cccc(c12)CC=C(C)C)O)c1ccccc1	-9.6
907980	o1c(C)c(c(=O)c2ccc(cc12)OCc1ccccc1)c1ccccc1	-9.6
9948081	c1(ccc2c(=O)cc(oc2c1)c1ccccc1)OC[C@H]1OC1	-9.6
100914052	c1(ccc(cc1)c1c(c(=O)c2ccccc2o1)O)N1CCOCCOCCOCCOCC1	-9.5
101499235	c1(cc(c(c(c1)OC)O[C@@H](CO)[C@@H](c1ccc(cc1)O)O)OC)[C@H]1Oc2cc(cc(c2C(=O)C1)O)O	-9.5
102066918	o1c(c(c(=O)c2c(c(c(cc12)OCc1ccccc1)OC)O)O)c1ccccc1	-9.5
102203496	o1c(cc(=O)c2c(cc(cc12)OCOC)OCOC)c1ccc(cc1)OCc1ccccc1	-9.5
10524567	O1c2cc3c(C(=O)C[C@@H](O3)c3ccc4c(CCC(C)(C)O4)c3)cc2CCC1(C)C	-9.5
11639597	c1(ccc2c(=O)cc(oc2c1)c1ccc(c(c1)O)O)OC[C@H](O)CNC(C)C	-9.5
11725803	O1c2cc(cc(c2C(=O)C[C@H]1c1cc(c(c(c1)CC=C(C)C)O)O)O)O	-9.5
12982116	c1(c(cc2c(C(=O)C[C@@H](O2)c2ccccc2)c1O)OC)C[C@H]1OC1(C)C	-9.5

5		
129834517	O1c2cc(cc(c2C(=O)C[C@H]1c1ccc(cc1)OC(=O)C)OC)OCc1ccccc1	-9.5
15485967	O1c2cc(c(c(c2C(=O)C[C@H]1c1ccc(cc1)O)O)CC=C(C)C)OC	-9.5
29920547	o1c(cc(=O)c2c(C)cc(cc12)OC(=O)OCC)c1ccccc1	-9.5
42608102	O1c2c3ccoc3c(c(c2C(=O)C[C@H]1c1ccc2c(OCO2)c1)O)OC	-9.5
467492	o1c(cc(=O)c2c(cc(cc12)OC(=O)C)OC(=O)C)c1ccccc1	-9.5
46850208	c1(c(cc2c(c(=O)cc(o2)c2ccc(c(c2)OC)O)c1O)O)C/C=C(\C)/CC[C@@H](C(C)(C)O)O	-9.5
5316782	O1c2cc(c3C(=O)C[C@H](Oc3c2C[C@H]1C(C)(C)O)c1ccc(cc1)O)O	-9.5
56649091	c1(c(c(c2c(=O)cc(oc2c1)c1ccccc1)O)O)OCCN1CCCCC1	-9.5
8395	o1c(cc(=O)c2ccc(cc12)OCC(=O)OCC)c1ccccc1	-9.5
101938669	c1(ccc(cc1)c1c(c(=O)c2ccccc2o1)O)N1OCCOCCOCCOCCOC1	-9.4
10449654	O1c2cc3c(c(=O)cc(o3)c3ccc(cc3)O)c(c2C=CC1(C)C)O	-9.4
10472357	o1c(cc(=O)c2c(c(c(cc12)O)NCCC(C)C)O)c1ccccc1	-9.4
10814209	c1(c(cc2c(c(=O)c(c(o2)c2ccc(cc2)OC)O)c1O)O)OS(O)(O)c1ccc(C)cc1	-9.4
11552747	c1(ccc2c(=O)cc(oc2c1)c1cc(cc(c1)O)O)OC[C@H](O)CNC(C)C	-9.4
11661570	c1(ccc2c(=O)cc(oc2c1)c1cc(cc(c1)OC)OC)OC[C@H](O)CNC(C)C	-9.4
11954210	c1(ccc2c(=O)c(c(oc2c1)c1ccccc1)O)O[C@@H]1O[C@@H]([C@H]([C@@H]([C@H]1O)O)O)CO	-9.4
12918915	c1(ccc2c(=O)c(coc2c1)c1ccccc1)O[C@@H](c1ccccc1)CCNC	-9.4
129224	c1(ccc2c(=O)cc(oc2c1)c1ccc(C)cc1)OC[C@@H](O)CNC(C)C	-9.4
129826769	S=C1c2ccccc2O[C@@H](C1)c1ccc(C)cc1	-9.4
14445197	c1(c(cc2c(c(=O)cc(o2)c2ccc(c(c2)O)OC)c1O)O)[C@@H]1O[C@H](C)[C@@H]([C@@H](C1)O)O	-9.4
146681574	o1c(c(c(=O)c2c(c3c(cc12)OCO3)O)OC(O)O)c1ccccc1	-9.4

15118758	c1(ccc2c(=O)cc(oc2c1)c1ccccc1O)O[C@@H]1O[C@@H]([C@H]([C@@H]([C@H]1O)O)O)CO	-9.4
182342	O1c2ccc3c(=O)c(coc3c2C=CC1(C)C)c1ccc2c(OCO2)c1	-9.4
19358579	o1c(cc(=O)c2c(cc(cc12)OCc1ccccc1)OC)c1ccc(c(c1)OC)OC	-9.4
20301090	O1c2ccccc2C(=O)C(=C)[C@H]1c1ccc(cc1)N(=O)=O	-9.4
42607867	c1(cc(c2C(=O)C[C@H](Oc2c1)c1ccccc1)O)OC[C@H]1OC1(C)C	-9.4
42608112	O1c2c(c(c(c(c2C(=O)C[C@H]1c1ccccc1)O)Cc1cc(ccc1O)OC)OC)OC	-9.4
44258305	c1(cc(c2c(=O)cc(oc2c1)c1ccc(cc1)O)OC)OC[C@H]1OC1(C)C	-9.4
44258732	o1c(c(c(=O)c2c(c(C)c(c(C)c12)OC)O)O)c1ccccc1	-9.4
44259050	O1c2cc3c(c(=O)c(c(o3)c3ccc(cc3)O)O)c(c2C=CC1(C)C)O	-9.4
491718	O1c2c(C)c(c(c(c2C(=O)C[C@H]1c1ccc(cc1)O)O)CC=C(C)C)O	-9.4
5317480	o1cc(c(=O)c2c(cc(c(c12)CC=C(C)C)O)O)c1ccc(cc1)O	-9.4
5318267	c1(ccc2c(=O)c(coc2c1)c1ccc(c(c1)O)OC)O[C@@H]1O[C@@H]([C@H]([C@@H]([C@H]1O)O)O)CO	-9.4
53982668	O1c2ccccc2C(=O)/C(=C\NCc2ccc(cc2)C(=O)O)/[C@H]1c1ccccc1	-9.4
54410	c1(ccc2c(=O)cc(oc2c1)c1ccccc1)OC[C@H](O)CNCCC	-9.4
56649090	c1(c(c(c2c(=O)cc(oc2c1)c1ccccc1)O)O)OCCN1CCOCC1	-9.4
101499234	c1(cc(c(c(c1)OC)O[C@H](CO)[C@H](c1ccc(cc1)O)O)OC)[C@H]1Oc2cc(cc(c2C(=O)C1)O)O	-9.3
101680705	c1(ccc2c(=O)cc(oc2c1)c1ccccc1)C(=O)CBr	-9.3
102577932	c1(cc(c2c(=O)cc(oc2c1)c1ccc(cc1)O)O)OS(C)(O)O	-9.3
11077963	o1c2ccccc2c(=O)c(c1c1ccccc1)[C@@H](c1ccccc1)O	-9.3
11132477	o1c2ccccc2c(=O)c(c1c1ccccc1)[C@@H](c1ccc(cc1)N(=O)=O)O	-9.3
11611332	c1(ccc2c(=O)cc(oc2c1)c1ccc(c(c1)OC)OC)OC[C@H](O)CNC(C)C	-9.3
11618226	c1(ccc2c(=O)cc(oc2c1)c1ccc(c(c1)O)O)OC[C@H](O)CNC(C)(C)C	-9.3
11718263	c1(ccc2c(c(=O)cc(o2)c2ccc(c(c2)O)O)c1)OC[C@H](O)CNC(C)(C)C	-9.3

11912	c1(ccc2c(=O)cc(oc2c1)c1ccccc1)OCCCN(C)C	-9.3
129730222	o1c(cc(=O)c2cc(C)cc(c12)C(=O)CC)c1ccccc1	-9.3
129821705	o1c(c(C)c(=O)c2cc(c(c(c12)N(C)C)O)O)c1ccc(cc1)O	-9.3
129824479	O1C2=CC=CCC2=C(C=C1C1=CCC(=CC1)C=O)NO	-9.3
129825943	o1c2cc(cc(c2c(=O)cc1c1ccccc1C(=O)CCCCC)O)OC	-9.3
13432335	o1c(cc(=O)c2c(C)cc(C)cc12)c1ccccc1	-9.3
14033984	O1c2c(cc3c(=O)c(c(oc3c2C=CC1(C)C)c1ccccc1)OC)OC	-9.3
154699919	O([C@@H]1O[C@@H]([C@H]([C@@H]([C@H]1O)O)O)C(=O)O)c1ccc(cc1)c1cc(=O)c2ccccc2o1	-9.3
16737249	O1c2cc(cc(c2C(=O)C[C@H]1c1ccc2c(C=CC(C)(C)O2)c1)O)O	-9.3
21325016	c1(cc(=O)c2cc(ccc2o1)C(=O)O)c1c(cccc1)OC[C@@H](CC)O	-9.3
2361	o1c(cc(=O)c2c3ccccc3ccc12)c1ccccc1	-9.3
23991884	o1c(C)c(c(=O)c2c(cc(cc12)OCc1ccc(o1)C(=O)O)O)c1ccccc1	-9.3
44258526	o1c(cc(=O)c2c3c(c(cc12)OC)OCO3)c1ccc2c(OCO2)c1	-9.3
44578052	o1c(cc(=O)c2c(c(c(c(c12)CN1CCCCC1)O)O)O)c1ccccc1	-9.3
53904139	c1(cccc(c1)c1cc(=O)c2ccccc2o1)S(=O)(=O)N	-9.3
5393156	o1c(cc(=O)c2c(C)cc(cc12)O)c1ccccc1	-9.3
66728267	c1(c(ccc(c1)c1coc2cc(cc(c2c1=O)O)O)O)CCC(C)(C)O	-9.3
101499236	c1(c(cc(cc1OC)c1cc(=O)c2c(cc(cc2o1)O)O)OC)O[C@H](CO)[C@@H](c1ccc(cc1)O)O	-9.2
101516665	O1c2cc(cc(c2C(=O)C[C@H]1c1ccc(cc1)OC(=O)C)O)OCC=C(C)C	-9.2
101843486	c1(ccc2c(C(=O)C[C@H](O2)c2ccccc2)c1)O[C@@H]1O[C@@H]([C@H]([C@@H]([C@H]1O)O)OC)CO	-9.2

10543665	c1(cc(=O)c2c(cccc2o1)OC)c1cccc(c1)C(F)(F)F	-9.2
10970624	O1c2c(c3c(C=CC(C)(C)O3)c(c2C(=O)C[C@H]1c1ccc(cc1)O)O)CO	-9.2
11149874	O1c2ccccc2C(=O)C[C@H]1c1ccc(cc1)N(=O)=O	-9.2
11545770	c1(ccc2c(=O)cc(oc2c1)c1cc(cc(c1)O)O)OC[C@H](O)CNC(C)(C)C	-9.2
11683340	c1(ccc2c(=O)cc(oc2c1)c1cc(cc(c1)OC)OC)OC[C@H](O)CNC(C)(C)C	-9.2
11847316	o1c2ccccc2c(=O)c(c1c1ccccc1)Cn1cncc1	-9.2
12984781<br>7	o1c(cc(=O)c2c(c(c(cc12)O)CCC(=C)C)O)c1ccc(cc1O)O	-9.2
14033976	O1c2cc(c3c(=O)cc(oc3c2C=CC1(C)C)c1ccc2c(OCO2)c1)OC	-9.2
15308126	c1(c(c(cc2oc(c(c(=O)c12)O)c1ccc(cc1)OC)OC)O)OS(O)(O)c1ccc(C)cc1	-9.2
15389725	c1(cc(=O)c2ccccc2o1)c1cccc(c1)S(=O)(=O)Cl	-9.2
15731439	o1c(cc(=O)c2ccc(c(c12)OC(=O)C)OC(=O)C)c1ccccc1	-9.2
24133364	o1c(C)c(c(=O)c2ccc(cc12)OCc1ccc(o1)C(=O)O)c1ccccc1	-9.2
3033107	c1(ccc(cc1)c1c(C)c(=O)c2ccccc2o1)OC[C@@H](O)CNC(C)(C)C	-9.2
41451	c1(ccc(cc1)c1c(C)c(=O)c2ccccc2o1)OC[C@@H](O)CNC(C)C	-9.2
42608049	O1c2cc(c3C(=O)C[C@H](Oc3c2CCC1(C)C)c1ccc(cc1O)O)O	-9.2
440215	c1(ccc2c(=O)c(coc2c1)c1ccccc1)O[C@@H]1O[C@@H]([C@H]([C@@H]([C@H]1O)O)O)CO	-9.2
44258693	O1c2c(cc3c(=O)c(c(oc3c2C=CC1(C)C)c1ccc(cc1)O)OC)OC	-9.2
44259326	O1c2cc(c3c(=O)c(c(oc3c2[C@@H](C)CC1(C)C)c1ccc(c(c1)O)O)O)O	-9.2
466268	o1c(cc(=O)c2ccc(cc12)OC)c1ccccc1	-9.2
46881227	c1(cc(c2C(=O)C[C@H](Oc2c1)c1ccccc1)O)O[C@@H]1O[C@@H]([C@H]([C@@H]([C@H]1O)O)O)CO	-9.2
49788612	o1c2cc(cc(c2c(=O)c(c1c1ccc2c(OCO2)c1)O)O)OC	-9.2
5272796	o1c2ccccc2c(=O)cc1c1ccc(cc1)C(=O)O	-9.2
5317478	o1cc(c(=O)c2c(c(c(cc12)O)CC=C(C)C)O)c1ccc(cc1)OC	-9.2
5317765	c1(c(c(=O)c2c(cc(cc2o1)O)O)O)c1ccc2OC([C@@H](Cc2c1)O)(C)C	-9.2
5378307	o1c(cc(=O)c2c3cc(ccc3ccc12)O)c1ccccc1	-9.2

564150	o1c(c(c(=O)c2ccc(cc12)OCc1ccccc1)OC)c1ccc(c(c1)OC)OC	-9.2
625630	o1c(cc(=O)c2c3ccccc3cc(c12)O)c1ccccc1	-9.2
625631	o1c(cc(=O)c2c3ccc(cc3ccc12)O)c1ccccc1	-9.2
66561376	o1c(c(c(=O)c2c(c3c(c(c12)OC)OCO3)O)OC)c1ccc2c(OCO2)c1	-9.2
676295	o1c2ccccc2c(=O)c(c1c1cccc(c1)O)O	-9.2
74819425	O1c2cc3c(c(c2C(=O)C[C@H]1c1ccccc1O)O)OCO3	-9.2
76333661	O(c1ccc2c(=O)cc(oc2c1)c1ccccc1)[C@@H]1O[C@@H]([C@H]([C@@H]([C@H]1O)O)O)CC(=O)C	-9.2
88881	o1c(cc(=O)c2c(cc(cc12)OC)OC)c1ccccc1	-9.2
9883305	o1c(c(c(=O)c2c(c3c(cc12)OCO3)O)O)c1ccc(cc1)O	-9.2
100633	o1c(c(c(=O)c2ccc3c(cco3)c12)OC)c1ccccc1	-9.1
101028402	o1c(cc(=O)c2c(c(c(cc12)O)NCC(C)C)O)c1ccccc1	-9.1
101238241	c1(cc2c(c(=O)c(co2)c2ccc(cc2)O)cc1C(=O)C)O[C@@H]1O[C@@H]([C@H]([C@@H]([C@H]1O)O)O)CO	-9.1
101843484	c1(cccc2oc(cc(=O)c12)c1ccc(cc1)O)O[C@@H]1O[C@@H]([C@H]([C@@H]([C@H]1O)O)OC)CO	-9.1
102017165	o1c(cc(=O)c2c(cc(c(c12)CCC(=C)C)O)O)c1ccccc1	-9.1
102064288	o1c(cc(=O)c2cccc(c12)/C=C/C)c1ccccc1	-9.1
102091267	O1c2ccccc2[C@H](C[C@H]1c1ccccc1)NOC(=O)C	-9.1
102369763	O1c2ccc3CC[C@H](Oc3c2C[C@@H]1C(C)(C)O)c1ccc(cc1)O	-9.1
10337211	O1c2cc(c(cc2C(=O)C[C@H]1c1ccc(cc1)O)CC=C(C)C)OC	-9.1
10546844	c1(coc2cc(cc(c2c1=O)OC)O)c1cc(c2OC(C=Cc2c1)(C)C)O	-9.1
10708855	c1(cc(=O)c2ccccc2o1)c1cccc(c1)CN=C=S	-9.1

11583077	c1(ccc2c(=O)cc(oc2c1)c1cc(c(c(c1)OC)OC)OC)OC[C@@H](O)CNC(C)C	-9.1
11612258	c1(ccc2c(=O)cc(oc2c1)c1cc(c(c(c1)OC)OC)OC)OC[C@@H](O)CNC(C)(C)C	-9.1
11834	c1(ccc2c(=O)cc(oc2c1)c1ccccc1)OC[C@@H](C)CN(C)C	-9.1
12043065	c1(cc(=O)c2c(cc(c(c2o1)[C@@H]1CN(C)CC[C@@H]1O)O)O)c1c(Cl)cccc1	-9.1
12043069	c1(cc(=O)c2c(cc(c(c2o1)c1cncnc1)O)O)c1c(Cl)cccc1	-9.1
12146121	o1c(cc(=O)c2c(cc(cc12)OCOC)OCC=C(C)C)c1ccccc1	-9.1
122835	O1c2cc(c(cc2C(=O)C[C@H]1c1ccc(cc1)O)CC=C(C)C)OC	-9.1
129684668	o1c(c(c(=O)c2c(c(c(c(c12)O)O)/C=C/C(=C)C)O)O)c1ccccc1	-9.1
129704118	o1c(cc(=O)c2c(cc(cc12)OCO)OCO)c1ccccc1	-9.1
129716149	c1(c(cc2c(c(=O)c(co2)c2ccc(cc2)O)c1O)O)[C@H]([C@@H]1OC1)CC=C(C)C	-9.1
129820755	c1(ccc2c(c(=O)c(c(o2)c2ccccc2)O)c1)[C@@H](O)CCCC	-9.1
129881487	o1c2ccc(cc2c(=O)cc1c1ccccc1C(=O)O)C(=O)O	-9.1
132533490	o1cc(c(=O)c2c(c(c(cc12)O)C[C@@H](C(=C)C)O)O)c1ccc2c(OCO2)c1	-9.1
135121498	c1(c(c(=O)c2c(cccc2o1)O)O)c1cc(c2c(c1)C2)OC	-9.1
136023343	c1(cc(=O)c2c(cc(c(c2o1)c1ccncc1)O)O)c1c(Cl)cccc1	-9.1
14187087	O1c2cc3c(C=CC(C)(C)O3)c(c2C(=O)C[C@H]1c1ccc(cc1)O)O	-9.1
14237660	c1(coc2cc(cc(c2c1=O)O)O)c1ccc2OC(C=Cc2c1)(C)C	-9.1
145706427	O1c2ccccc2C(=O)C[C@H]1c1cc(cc(c1)OC)O	-9.1
15160702	O1c2c(ccc3OC(C)(C)C=Cc23)C(=O)C[C@H]1c1ccc(c(c1)OC)OC	-9.1

1889	O1c2cc(ccc2C(=O)C[C@H]1c1ccc(cc1)O)O	-9.1
1890	O1c2cc(ccc2C(=O)C[C@H]1c1ccccc1)O	-9.1
21673114	o1c(cc(=O)c2c(cc(cc12)OCOC)O)c1ccccc1	-9.1
23286063	o1c(c(c(=O)c2cc(ccc12)C(=O)O)O)c1ccccc1	-9.1
238782	O1c2cc(cc(c2C(=O)C[C@H]1c1ccccc1)O)O	-9.1
25030431	o1c(c(c(=O)c2cc(ccc12)NC(=O)CCC(=O)O)O)c1ccc(c(c1)O)O	-9.1
25128870	o1c(cc(=O)c2c(cc(cc12)OCC1=NNNN1)OC)c1ccc(c(c1)OC)OC	-9.1
42607869	O1c2c(c(cc(c2C(=O)C[C@H]1c1ccccc1)O)O)C/C=C(\C)/CO	-9.1
42607870	O1c2c(c(cc(c2C(=O)C[C@H]1c1ccccc1)O)O)C/C=C(\C)/C=O	-9.1
42607962	O1c2c(c(cc3OC(C)(C)C=Cc23)O)C(=O)C[C@H]1c1ccc(cc1)OC	-9.1
44481784	o1cc(c(=O)c2c(cc(cc12)OC)O)c1ccc(c(c1)C[C@@H](C(=C)C)O)O	-9.1
5317300	c1(ccc(c(c1O)CC=C(C)C)O)c1coc2c(c1=O)ccc(c2)O	-9.1
5494866	o1cc(c(=O)c2c(cc(cc12)O)O)c1ccc(c(c1)CC=C(C)C)O	-9.1
58089059	o1c(c(c(=O)c2cc(ccc12)NC(=O)CCC(=O)O)O)c1ccc(cc1)O	-9.1
636525	O1c2c(c(cc3OC(C)(C)C=Cc23)O)C(=O)C[C@H]1c1ccc(c(c1)O)OC	-9.1
637111	O1c2c(c(c3C=CC(C)(C)Oc3c2CC=C(C)C)O)C(=O)[C@@H]([C@H]1c1ccccc1O)O	-9.1
6450959	o1cc(c(=O)c2c(cc(c(c12)C/C=C(\C)/CO)O)O)c1ccc(cc1)O	-9.1
676290	o1c2ccccc2c(=O)cc1c1ccc(cc1O)O	-9.1
688857	O1c2cc(ccc2C(=O)C[C@H]1c1ccccc1)O	-9.1
86083806	O1C2=C(CC=CC2)C=C/C/1=C\1/C=CC(=CC1)NC(=O)C	-9.1
100943382	Clc1cc(cc(c1OC)S(=O)(=O)NC(C)C)c1cc(=O)c2ccccc2o1	-9.0
101028404	o1c(cc(=O)c2c(cc(c(c12)NC(C)C)O)O)c1ccccc1	-9.0
101214939	c1(ccc(cc1)c1c(c(=O)c2ccccc2o1)O)N1CCN(CC1)[C@@H]1C(=CNC=C1)S(O)(O)O	-9.0

10335613	o1c(cc(=O)c2c(c(c(cc12)O)NC(C)C)O)c1ccccc1	-9.0
10357090	c1(cccc2c(=O)c(coc12)c1ccccc1)OC[C@H]1OC1	-9.0
10450773	o1c(cc(=O)c2c(c(c(cc12)O)/C=C/C(C)C)O)c1ccc(cc1O)O	-9.0
10659060	o1c2ccccc2c(=O)cc1c1cccc(c1)CC#N	-9.0
10736576	O1c2cc3c(c(=O)c(co3)c3ccc(cc3)O)c(c2C=CC1(C)C)OC	-9.0
10761522	[C@H]1(CC(=O)c2c(cc(cc2O1)O)O)c1cc(c2OC(C=Cc2c1)(C)C)OC	-9.0
11142360	o1cc(c(=O)c2CC3=C(Cc12)OCO3)c1cc(c2c(OCO2)c1)OC	-9.0
11177340	o1c(cc(=O)c2c(cc(cc12)OCOCCOC)OCC=C(C)C)c1ccccc1	-9.0
11186717	O1c2c(cc(cc2C=CC1(C)C)c1coc2cc(ccc2c1=O)O)O	-9.0
11742974	c1(c2c(C=CC(C)(C)O2)c(c2C(=O)C[C@H](Oc12)c1ccc(cc1)O)O)C[C@H]1OC1(C)C	-9.0
11810763	o1cc(c(=O)c2c(c3c(cc12)OCO3)OC)c1cc(c2c(OCO2)c1)OC	-9.0
118907	o1c(cc(=O)c2c(cc(cc12)OCC(=O)OCC)OCC(=O)OCC)c1ccccc1	-9.0
12988126<br>9	o1c2ccc(cc2c(=O)c(c1c1ccc2c(OCO2)c1)O)C(=O)O	-9.0
13393782	[C@H]1(CC(=O)c2ccccc2O1)c1cccc(F)c1	-9.0
13908069<br>7	o1c(c(c(=O)c2c(cc(cc12)OCC=C(C)C)O)O)c1ccc(c(c1)O)OC	-9.0
15160703	O1c2c(cc(c3OC(C)(C)C=Cc23)OC)C(=O)C[C@H]1c1ccc(c(c1)OC)OC	-9.0
242065	[C@H]1(CC(=O)c2ccccc2O1)c1ccc(Cl)cc1	-9.0
24960482	c1(ccc2c(=O)cc(oc2c1)c1cc(cc(c1)O)O)OC[C@@H](O)CNC(C)C	-9.0
3070240	c1(c(C)c(=O)c2ccc(c(c2o1)CN(C)C)OC)c1ccc(F)cc1	-9.0
44380969	o1c(cc(=O)c2c(cc(c(c12)NCCC(C)C)O)O)c1ccccc1	-9.0
44584124	O1c2cc3c(C=CC(C)(C)O3)c(c2C(=O)C[C@H]1c1ccccc1O)O	-9.0
462691	O1c2c(cccc2C(=O)C[C@H]1c1ccccc1)CC(=O)O	-9.0
466277	Brc1ccc2c(=O)cc(oc2c1)c1ccccc1	-9.0
5270553	O1c2ccc(cc2C(=O)C[C@H]1c1ccccc1)C(=O)O	-9.0

5271991	o1c(cc(=O)c2c(cc(c(c12)OC)O)O)c1c(ccc(c1OC)O)O	-9.0
5281611	o1c2cc(ccc2c(=O)c(c1c1ccc(cc1)O)O)O	-9.0
5281617	o1c(cc(=O)c2c(cc(cc12)OC)O)c1ccc(cc1)O	-9.0
5281894	o1c(cc(=O)c2ccc(cc12)O)c1ccccc1	-9.0
5282073	o1c(cc(=O)c2ccc(cc12)O)c1ccc(cc1)O	-9.0
5320053	o1cc(c(=O)c2ccc(cc12)O)c1ccc(c(c1)CC=C(C)C)O	-9.0
53747690	c1(cc(c2c(=O)cc(oc2c1)c1ccccc1)O)C(=O)CBr	-9.0
57340186	o1c2cc(ccc2c(=O)cc1c1ccc(cc1)/C=C/C(=O)OCC)O	-9.0
624794	o1c2ccccc2c(=O)c(c1c1ccc2c(OCO2)c1)O	-9.0
637879	o1cc(c(=O)c2c(c3c(cc12)OCO3)OC)c1ccc2c(OCO2)c1	-9.0
85751070	N(C1=C[C@H](Oc2ccccc12)c1ccccc1)NC(=S)N	-9.0
90753092	o1c(c(c(=O)c2c(C)c(C)c(C)cc12)O)c1ccccc1	-9.0
91557562	O1c2cc(ccc2C(=O)C[C@H]1c1ccc(cc1O)O)O	-9.0
95400994	O1c2ccc3ccccc3c2C(=O)C[C@H]1c1ccccc1	-9.0
9973510	o1c2ccc(cc2c(=O)cc1c1ccccc1OC(C)C)C(=O)O	-9.0
101843485	c1(ccc2c(C(=O)C[C@@H](O2)c2ccccc2)c1)O[C@@H]1O[C@@H]([C@H]([C@@H]([C@H]1O)O)OC)CO	-8.9
101974135	O1c2c(c(cc(c2Cc2c(=O)oc(c(C)c2O)CC)O)O)C(=O)C[C@H]1c1ccccc1	-8.9
102307255	c1(c(=O)c2ccccc2oc1c1ccccc1)C(F)(F)F	-8.9
10244935	o1c(cc(=O)c2c(c(c(cc12)O)NCCC)O)c1ccccc1	-8.9
102674	O1c2ccccc2[C@H](C[C@H]1c1ccccc1)NN	-8.9
10543327	c1(cc(=O)c2ccccc2o1)c1cccc(c1)CBr	-8.9
10680	o1c2ccccc2c(=O)cc1c1ccccc1	-8.9
10889522	O=C1c2ccccc2N[C@@H](C1)c1ccccc1	-8.9

11247278	O1c2c(c(cc(c2C(=O)C[C@H]1c1ccc(cc1)OC(=O)C)O)OC(=O)C)CC=C(C)C	-8.9
11289628	O1c2cc(cc(c2C(=O)C[C@H]1c1cc(cc(c1)O)O)O)OC	-8.9
11382659	c1(cc(c(c(c1)CC=C(C)C)OC)O)[C@H]1Oc2cc(ccc2C(=O)C1)O	-8.9
11772232	o1c(cc(=O)c2c(cc(cc12)OCOCCOC)O)c1ccccc1	-8.9
12136839	O1c2ccccc2C(=O)C[C@@H]1c1ccccc1	-8.9
121487929	O1c2ccccc2C(=O)C[C@@H]1c1ccccc1	-8.9
129858341	O1c2c(c(ccc2C(=O)C([C@@]1(c1ccccc1)O)(O)O)OC)OC	-8.9
132279663	o1cc(c(=O)c2c(c(c(cc12)O)OC)OC)c1cccc2OCOc12	-8.9
14791391	o1c2cc(ccc2c(=O)cc1c1ccc(cc1)N(=O)=O)O	-8.9
14791392	o1c2cc(ccc2c(=O)cc1c1ccc(cc1)N)O	-8.9
15342845	o1c(c(c(=O)c2c(cc(cc12)O)OC(=O)c1ccccc1)OC)c1ccccc1	-8.9
15389441	O1c2cc3c(c(=O)c(co3)c3cc(c(cc3OC)OC)OC)cc2C=CC1(C)C	-8.9
15817847	o1c2cc(c(cc2c(=O)c(c1c1ccc(c(c1)OC)O)O)O)O	-8.9
176925	O1c2ccccc2C(=O)C[C@H]1c1ccccc1O	-8.9
188308	O1c2cc(c(c(c2C(=O)C[C@H]1c1ccc(cc1)O)O)O)O	-8.9
188424	O1c2cc(cc(c2C(=O)C[C@@H]1c1ccc(cc1)O)OC)O	-8.9
19835350	c1(cccc2c(=O)cc(oc12)c1ccccc1)OC[C@@H]1OC1	-8.9
20399	O1c2cc(ccc2C(=O)[C@H]([C@@H]1c1cc(c(c(c1)O)O)O)O)O	-8.9
21315742	c1(cc(=O)c2cc(ccc2o1)C(=O)O)c1c(ccc(C)c1)OC(C)C	-8.9
23644933	o1c2cc(ccc2c(=O)c(c1n1cncc1)c1ccccc1)OCc1ccccc1	-8.9
23644934	o1c2cc(ccc2c(=O)c(c1n1cncc1)c1ccc(cc1)OC)OCc1ccccc1	-8.9
23644952	c1(c(c(=O)c2ccc(cc2o1)OCc1ccccn1)c1ccccc1)SCc1ccncc1	-8.9
250312	O1C(=CC(=C2CC=CC=C12)NO)c1ccccc1	-8.9

25073757	O1c2cc(cc(c2C(=O)C[C@H]1c1cc(ccc1O)O)O)O	-8.9
3078	o1c(c(C)c(=O)c2ccc(c(c12)CN(C)C)OC)c1ccccc1	-8.9
343080	o1cc(c(=O)c2ccc(cc12)OCC=C(C)C)c1cc2c(cc1OC)OCO2	-8.9
3534982	O1c2ccccc2C(=O)C[C@H]1c1cccc(c1)O	-8.9
369599	o1c(cc(=O)c2c(c(C)c(cc12)OC)O)c1ccccc1	-8.9
42607959	c1(cc(c(c(c1)CC=C(C)C)OC)/C=C/C(C)(C)O)[C@H]1Oc2cc(cc(c2C(=O)C1)O)O	-8.9
439246	O1c2cc(cc(c2C(=O)C[C@H]1c1ccc(cc1)O)O)O	-8.9
44380895	o1c(cc(=O)c2c(cc(c(c12)NCC(C)C)O)O)c1ccccc1	-8.9
45933941	o1c2ccccc2c(=O)cc1c1cc(cc(c1)O)O	-8.9
511789	O1c2c(c(ccc2C(=O)C[C@H]1c1ccc(cc1)O)OC)CC=C(C)C	-8.9
5270543	Fc1ccc2c(O[C@@H](CC2=O)c2ccccc2)c1	-8.9
5280863	o1c(c(c(=O)c2c(cc(cc12)O)O)O)c1ccc(cc1)O	-8.9
5281616	o1c(c(c(=O)c2c(cc(cc12)O)O)O)c1ccccc1	-8.9
5281954	o1c(cc(=O)c2c(cc(cc12)OC)O)c1ccccc1	-8.9
5317287	o1c(c(c(=O)c2c(c(c(cc12)OC)OC)O)O)c1ccc(c(c1)O)OC	-8.9
53247947	c1(ccc(cc1)[C@H]1CC(=O)c2ccccc2O1)O[C@@H]1O[C@@H]([C@H]([C@@H]([C@H]1O)O)O)CO	-8.9
5391140	o1c(cc(=O)c2ccc(cc12)O)c1cccc(c1)O	-8.9
5393152	o1c(c(c(=O)c2ccc(cc12)O)O)c1ccccc1	-8.9
5465885	O1c2ccccc2[C@H](C[C@H]1c1ccccc1)NN	-8.9
54721824	O1c2c(c(cc(c2Cc2c(=O)oc(c(C)c2O)CC)O)O)C(=O)C[C@H]1c1ccccc1	-8.9
5491798	o1c(c(c(=O)c2c(cc(c(c12)OC)OC)O)O)c1ccccc1	-8.9
6077518	O1c2ccccc2[C@H](C[C@H]1c1ccccc1)NOCCN(CC)CC	-8.9
676310	o1c(cc(=O)c2ccc(c(c12)O)O)c1cccc(c1)O	-8.9
688659	o1c(c(c(=O)c2cc(ccc12)O)O)c1ccccc1	-8.9
688861	O1c2ccccc2C(=O)C[C@H]1c1cccc(c1)O	-8.9

689010	O1c2ccccc2C(=O)C[C@@H]1c1ccccc1	-8.9
70696494	O1c2cc(cc(c2C(=O)C[C@H]1c1cc(cc(c1)O)O)O)OC	-8.9
71777365	O1c2ccccc2C(=O)C[C@@H]1c1ccccc1	-8.9
932	O1c2cc(cc(c2C(=O)C[C@H]1c1ccc(cc1)O)O)O	-8.9
101028403	o1c(cc(=O)c2c(c(c(cc12)O)NCC=C)O)c1ccccc1	-8.8
101624373	O1c2cc(ccc2[C@H](C[C@@H]1c1ccccc1)O)OC	-8.8
101643010	O1c2c(c(cc(c2C(=O)[C@@H]([C@H]1c1ccc(cc1)O)O)O)O)CC=C(C)C	-8.8
101932121	c1(cc2c(c(=O)cc(o2)c2ccc(cc2)O)c(c1OC)O)O[C@@H]1O[C@H]([C@@H]([C@H]([C@H]1O)O)O)C	-8.8
102017163	o1c(c(c(=O)c2c(cc(c(c12)CCC(=C)C)O)O)O)c1ccccc1	-8.8
102154220	O1c2cc3c(c(=O)cc(o3)c3ccc(cc3)O)c(c2[C@@H](CC1(C)C)O)O	-8.8
10491973	o1c(cc(=O)c2c(cccc12)OCC)c1ccccc1	-8.8
10612616	o1c(cc(=O)c2c(cccc12)OCC=C)c1ccccc1	-8.8
10848398	o1c2cc(ccc2c(=O)cc1c1ccc(C)cc1)O	-8.8
12071400	c1(cc(cc2oc(c(c(=O)c12)O)c1ccc(c(c1)OC)OC)OC)OS(O)(O)c1ccc(C)cc1	-8.8
12109666	O1c2c(ccc3OC(C)(C)C=Cc23)C(=O)C[C@H]1c1ccc(cc1)OC	-8.8
122366411	c1(ccc(cc1)c1cc(=O)c2ccccc2o1)OS(O)(O)C(F)(F)F	-8.8
122383344	O1c2cc(c(cc2C(=O)C[C@H]1c1ccc(cc1)O)C[C@@H](C(=C)C)O)O	-8.8
12261178	o1c2ccccc2c(=O)cc1c1ccc(cc1)C=O	-8.8
12813780	O1c2ccccc2C(=O)C[C@H]1c1cccc(C)c1	-8.8
12813783	o1c2ccccc2c(=O)cc1c1cccc(C)c1	-8.8

129633578	o1c(c(c(=O)c2ccc(c(c12)OC)OC)O)c1ccc(c(c1)OC)OC	-8.8
129813354	O1c2cc(ccc2C(=O)C[C@H]1c1cc(c(c(c1)O)O)O)OC	-8.8
129824490	O1C2=CC=CCC2=C(C=C1c1ccc(cc1)C=O)NOCC	-8.8
129834540	c1(c(cc2c(c(=O)cc(o2)c2ccc(C)cc2)c1O)O)S(O)(O)O	-8.8
132540297	o1c2cc(cc(c2c(=O)c(c1c1ccc(c(c1)OC(=O)C)OC(=O)C)O)O)O	-8.8
133611769	c1(ccc2c(=O)c(coc2c1)c1cc(cc(c1)OC)O)O[C@H]1O[C@H]([C@@H]([C@H]([C@H]1O)O)O)CO	-8.8
137327	o1c(cc(=O)c2cc(C)cc(c12)CC(=O)O)c1ccccc1	-8.8
13908971	O1c2cc(c3c(=O)c(coc3c2C[C@H]1C(=C)C)c1ccc(cc1O)O)O	-8.8
13942547	o1c(c(c(=O)c2c(cc(c(c12)OC)OCC=C(C)C)OC)OC)c1ccc2c(OCO2)c1	-8.8
15939984	o1c2ccc(C)cc2c(=O)c(c1c1ccc2c(OCO2)c1)O	-8.8
161860	o1c2ccccc2c(=O)cc1c1ccccc1O	-8.8
165506	O1c2ccccc2C(=O)C[C@H]1c1ccc(cc1)O	-8.8
19972541	o1c(c(C)c(=O)c2cccc(c12)C(=O)OCC)c1ccccc1	-8.8
20522556	c1(c(c(=O)c2c(cc(cc2o1)OC)O)OC)c1ccc(c(c1)OC)OC(=O)C(C)(C)C	-8.8
229016	o1c2ccccc2c(=O)cc1c1ccc(cc1)O	-8.8
3070241	c1(c(C)c(=O)c2ccc(c(c2o1)CN(CC)CC)OC)c1ccc(F)cc1	-8.8
343079	o1cc(c(=O)c2ccc3c(OCO3)c12)c1ccc2c(OCO2)c1	-8.8
41449	c1(ccc(cc1)c1cc(=O)c2ccccc2o1)OC[C@@H](O)CNC(C)C	-8.8
41450	c1(ccc(cc1)c1cc(=O)c2ccccc2o1)OC[C@@H](O)CNC(C)(C)C	-8.8
42607995	O1c2c(c(c(c(c2C[C@@H](C(=C)C)O)O)CC=C(C)C)O)C(=O)C[C@H]1c1ccc(c(c1)O)O	-8.8
44257318	o1cc(c(=O)c2c(cc(c(c12)C/C=C(\C)/CO)O)O)c1ccc(cc1O)O	-8.8

44380894	o1c(cc(=O)c2c(cc(c(c12)NCCC)O)O)c1ccccc1	-8.8
44380950	o1c(cc(=O)c2c(cc(c(c12)NCC=C)O)O)c1ccccc1	-8.8
462697	O1c2cc(cc(c2C(=O)[C@H]([C@H]1c1ccc(c(c1)OCc1ccccc1)OC)OC)O)O	-8.8
5280362	c1(c(=O)c2c(cc(cc2oc1c1ccc(c(c1)O)O)O)O)OS(=O)(=O)O	-8.8
5280443	o1c(cc(=O)c2c(cc(cc12)O)O)c1ccc(cc1)O	-8.8
5281607	o1c(cc(=O)c2c(cc(cc12)O)O)c1ccccc1	-8.8
5281691	o1c(c(c(=O)c2c(cc(cc12)OC)O)O)c1ccc(c(c1)O)O	-8.8
5281692	o1c2cc(ccc2c(=O)c(c1c1cc(c(c(c1)O)O)O)O)O	-8.8
5281814	o1cc(c(=O)c2c(c(c(cc12)O)CC=C(C)C)O)c1ccc(cc1)O	-8.8
5282154	o1c2cc(cc(c2c(=O)c(c1c1cc(c(c(c1)OC)O)O)O)O)O	-8.8
5315202	o1c(cc(=O)c2c(cc(cc12)O)OC)c1ccc(cc1)O	-8.8
5320287	o1c(c(c(=O)c2c(cc(cc12)OC)O)O)c1ccc(c(c1)O)OC	-8.8
5321435	o1c(c(c(=O)c2c(c(c(cc12)O)OC)O)O)c1ccc(c(c1)OC)O	-8.8
5326329	o1cc(c(=O)c2ccc(c(c12)CC=C(C)C)O)c1ccc(c(c1)O)OC	-8.8
5353357	o1c(cc(=O)c2cc(c(cc12)O)O)c1ccccc1	-8.8
5356690	O1c2ccccc2[C@H](C[C@H]1c1ccccc1)NO	-8.8
5362017	o1c(c(c(=O)c2c(cc(c(c12)O)O)O)O)c1ccccc1	-8.8
5378945	o1cc(c(=O)c2c(c(c(cc12)O)C/C=C(\C)/CO)O)c1ccc(cc1)O	-8.8
5490139	O1c2cc3c(c(=O)c(co3)c3ccc(cc3)O)c(c2C=CC1(C)C)O	-8.8
56649088	o1c(cc(=O)c2c(c(c(cc12)OCC=C)O)O)c1ccccc1	-8.8
57340184	c1(cc(=O)c2ccc(cc2o1)O)c1ccc(F)cc1	-8.8
57368437	o1c(c(c(=O)c2cc(cc(c12)O)O)O)c1ccc(cc1)O	-8.8
602425	O1c2c(c(ccc2C(=O)[C@H]([C@@H]1c1ccc(c(c1)OC)OC)O)OC)OC	-8.8
6453535	o1c(c(c(=O)c2c(c(c(cc12)OC)OC)O)O)c1ccc(c(c1)OC)O	-8.8
676309	o1c(cc(=O)c2ccc(c(c12)O)O)c1ccccc1O	-8.8

688859	O1c2ccccc2C(=O)C[C@H]1c1ccc(cc1)O	-8.8
71588424	O1C2=CC(=O)[C@H]3C(=O)C=C(O[C@H]3C2=C([C@H](C1(C)C)O)O)c1ccc(cc1)O	-8.8
71621984	c1(cc(c2c(=O)c(coc2c1)c1ccc(cc1)O)O)O[C@@H]1O[C@@H]([C@H]([C@@H]([C@H]1O)O)OC)CO	-8.8
71621987	c1(ccc2c(=O)c(coc2c1)c1ccc(cc1)O)O[C@@H]1O[C@@H]([C@H]([C@@H]([C@H]1O)O)OC)CO	-8.8
86236608	c1(ccc2c(=O)cc(oc2c1)c1cc(cc(c1)OC)OC)OC[C@@H]1OC1	-8.8
9950264	c1(c(cc2c(c(=O)cc(o2)c2ccccc2)c1O)O)OS(=O)(=O)O	-8.8
100943385	Clc1cc(cc(c1OC)S(=O)(=O)Cl)c1cc(=O)c2ccccc2o1	-8.7
101591280	c1(ccc2c(c(=O)cc(o2)c2ccc(c(c2)OC)O)c1OC)OCCCCC	-8.7
101680706	c1(c(ccc2c(=O)cc(oc12)c1ccccc1)C(=O)CBr)C(=O)CBr	-8.7
101890693	O1c2c(C)c(c(C)c(c2C(=O)C[C@H]1c1ccccc1)OC)O	-8.7
101930613	O1c2ccccc2C=C/C/1=C\1/CC=C(C=C1)NC(C)(C)C	-8.7
10251	O1c2ccccc2C(=O)C[C@H]1c1ccccc1	-8.7
10498462	O1c2cc(c(c(c2C(=O)C[C@H]1c1ccc(c(c1)O)O)O)C(C)(C)C=C)O	-8.7
10761665	O1c2cc(c(c(c2C(=O)C[C@H]1c1ccc(c(c1)OC)O)O)C(C)(C)C=C)O	-8.7
11032058	[C@@H]1(CC(=O)c2ccccc2O1)c1ccc(F)cc1	-8.7
11095	O1c2cc(cc(c2C(=O)C[C@H]1c1ccc(c(c1)O)O)O)O	-8.7
12049385	O1/C(=C\2/C=CC(=CC2)N(C)C)/C=Cc2ccc(cc12)N(CC)CC	-8.7
12114693	O1[C@@H](CC(=O)c2c(cc(c(c12)C(C)(C)C=C)O)O)c1ccc(cc1)O	-8.7
12132943	O1c2c(c(c(C)c(c2C(=O)C[C@H]1c1ccccc1)O)OC)C=O	-8.7
12310641	O1c2cc(ccc2C(=O)[C@H]([C@@H]1c1ccc(c(c1)O)O)O)O	-8.7
1232440	O1c2ccccc2CC[C@@H]1c1ccccc1	-8.7
12753173	[C@H]1(CC(=O)c2ccccc2O1)c1c(Cl)cc(cc1)Cl	-8.7

129689875	o1c(cc(=O)c2c(cccc12)OC(O)(O)O)c1ccccc1	-8.7
129762742	O1c2cc(cc(c2C(=O)C([C@@H]1c1ccc(cc1)OC)(O)O)O)OC	-8.7
129787765	O1C(=CC(=C2CC=CC=C12)NN)c1ccccc1	-8.7
129824456	O1[C@H](C=C(c2cc(ccc12)C=O)NO)c1ccccc1	-8.7
129830239	c1(cc(c(cc1c1c(c(=O)c2c(cc(cc2o1)O)O)O)O)O)S(=O)(=O)c1ccc(C)cc1	-8.7
129847910	O1c2cc(cc(c2[C@H](C(=O)[C@H]1c1cccc(c1)OC)O)O)O	-8.7
129864265	O1c2c(cccc2C(=O)C[C@@]1(c1ccccc1)O)OC	-8.7
13291608	o1c(c(c(=O)c2c(c(c(cc12)OC)OC)O)O)c1ccccc1	-8.7
133064953	o1c(c(c(=O)c2c(cccc12)CC#C)N)c1ccccc1	-8.7
137350369	c1(cccc2c(=O)c(coc12)c1ccccc1)[C@H]1O[C@@H]([C@H]([C@@H]([C@H]1O)O)O)CO	-8.7
139584124	o1cc(c(=O)c2c(cc(cc12)OC)O)c1ccc(cc1)OCC=C(C)C	-8.7
14034288	O1c2c(c(cc(c2C(=O)C[C@H]1c1ccccc1O)O)OC)O	-8.7
14057034	O1c2cc(c3c(=O)c(coc3c2C=CC1(C)C)c1cc(c(cc1OC)O)OC)O	-8.7
14704648	o1c(c(c(=O)c2c(c3c(cc12)OCO3)OC)OC)c1cc(c2c(OCO2)c1)OC	-8.7
14730798	O1c2cc(ccc2C(=O)[C@H]([C@@H]1c1ccccc1)O)O	-8.7
1686	c1(cc(=O)c2ccccc2o1)c1ccc(Br)cc1	-8.7
177731	o1cc(c(=O)c2ccc(cc12)OCC=C(C)C)c1ccc(cc1)OC	-8.7
21325023	o1c2cc(ccc2c(=O)cc1c1ccccc1OC(C)C)C(=O)O	-8.7

21576252	o1cc(c(=O)c2c(cc(cc12)OCC=C(C)C)O)c1ccc(cc1O)O	-8.7
21636237	O1c2c(c(cc(c2C(=O)C[C@H]1c1ccccc1OC)O)OC)OC	-8.7
2734580	O1c2ccc(cc2C(=O)C[C@H]1c1ccccc1)O	-8.7
3070247	c1(c(C)c(=O)c2ccc(c(c2o1)CN1CCCC1)OC)c1ccc(Cl)cc1	-8.7
378567	O1c2cc(cc(c2C(=O)C[C@H]1c1ccccc1)OC)OC	-8.7
3870517	O1c2cc(cc(c2C(=O)C[C@H]1c1ccc(cc1)OC(=O)C)O)OC(=O)C	-8.7
42607996	O1c2c(c(cc(c2C(=O)C[C@H]1c1ccc(c(c1)O)O)O)O)C/C=C(\C)/COC(=O)C	-8.7
439652	O1c2ccccc2C(=O)C[C@H]1c1ccccc1	-8.7
441251	o1cc(c(=O)c2c(c(c(cc12)OC)CC=C(C)C)O)c1ccc(cc1O)O	-8.7
44258533	o1c(cc(=O)c2c(c(c(cc12)OC)OC)OC)c1cc(c(c(c1)OC)OCC=C(C)C)OC	-8.7
44259869	o1c(c(c(=O)c2c(c(c(cc12)O)OC)O)O)c1ccc(c(c1)OC)OC	-8.7
50909808	o1cc(c(=O)c2c(cc(cc12)O)O)c1ccc2c(OCO2)c1	-8.7
5281606	c1(c(c(=O)c2c(cc(c(c2o1)OC)OC)O)OC)c1cccc(Cl)c1O	-8.7
5281654	o1c2cc(cc(c2c(=O)c(c1c1ccc(c(c1)OC)O)O)O)O	-8.7
5281697	o1c(cc(=O)c2c(c(c(cc12)O)O)O)c1ccc(cc1)O	-8.7
5281703	o1c(cc(=O)c2c(cc(c(c12)OC)O)O)c1ccccc1	-8.7
5281789	o1cc(c(=O)c2c(cc(cc12)O)O)c1ccc(c(c1O)CC=C(C)C)O	-8.7
5317481	o1cc(c(=O)c2c(cc(c(c12)C/C=C(\C)/CO)O)O)c1ccc(c(c1)O)OC	-8.7
5317756	o1c(=O)c(cc2c(c(c(cc12)O)CC=C(C)C)OC)c1ccc(cc1O)O	-8.7
5320945	o1c(c(c(=O)c2c(cc(cc12)OC)O)O)c1ccc(c(c1)OC)O	-8.7
53247945	c1(ccc(cc1)[C@H]1CC(=O)c2ccccc2O1)O[C@@H]1O[C@@H]([C@H]([C@@H]([C@H]1O)O)OC)CO	-8.7
53249095	O1c2cc3c(C=CC(C)(C)O3)c(c2C(=O)C[C@H]1c1ccc(cc1O)O)O	-8.7
5360930	o1c(c(c(=O)c2c(cc(c(c12)O)O)O)OC)c1ccccc1	-8.7
5377945	o1c(c(c(=O)c2c(c(c(cc12)O)OC)O)O)c1ccc(cc1)O	-8.7
54073202	c1(cc(c2c(O[C@@H](CC2=O)c2ccc(c(c2)O)OC)c1)O)OCCBr	-8.7

5466136	o1c2cc(c(c(c2c(=O)c(c1c1ccc2c(OCO2)c1)OC)O)OC)OC	-8.7
5466137	o1c2cc(cc(c2c(=O)c(c1c1ccc2c(OCO2)c1)OC)O)OC	-8.7
5468257	O1c2ccccc2[C@H](C[C@H]1c1ccccc1)NN	-8.7
5481228	o1cc(c(=O)c2c(cc(cc12)O)O)c1c(cc(c(c1)CC=C(C)C)O)O	-8.7
5487855	o1c2cc(cc(c2c(=O)c(c1c1ccc(c(c1)OC)OC)O)O)O	-8.7
555407	o1c(nc(=O)c2ccccc12)c1ccccc1	-8.7
56658060	O1c2cc(cc(c2C(=O)[C@H]([C@@H]1c1ccc(c(c1)OC)O)O)O)O	-8.7
57340187	o1c2cc(ccc2c(=O)cc1c1ccc(cc1)/C=C/C(=O)O)O	-8.7
636496	O1c2cc3c(c(=O)c(co3)c3ccc(cc3O)OC)c(c2C=CC1(C)C)OC	-8.7
66783912	c1(cc(=O)c2c(c3c(cc2o1)OCO3)O)c1ccc(cc1)OCCN(CC)CC	-8.7
66783913	o1c(cc(=O)c2c(c3c(cc12)OCO3)O)C1=CCC(=CC1)OC	-8.7
676034	o1c2ccc(cc2c(=O)cc1c1ccccc1OC)O	-8.7
676289	o1c2ccccc2c(=O)cc1c1cccc(c1O)O	-8.7
688835	o1c2ccc(cc2c(=O)c(c1c1cccc(c1)OC)O)OC	-8.7
688836	o1c(c(c(=O)c2ccc(cc12)OC)O)c1cccc(c1)OC	-8.7
69030367	o1c2cc(ccc2c(=O)cc1c1cc(cc(c1)OC)OC)O	-8.7
69234627	O([C@@]1(CCc2ccccc2O1)c1ccccc1)[C@@H]1O[C@@H]([C@H]([C@@H]([C@H]1O)O)O)CO	-8.7
72279	o1c(cc(=O)c2cc(ccc12)O)c1ccccc1	-8.7
73202	O1c2cc(cc(c2C(=O)[C@@H]([C@H]1c1ccccc1)O)O)O	-8.7
73829901	O1c2cc3c(c(c2C(=O)[C@@H]([C@H]1c1ccccc1)O)OC)OCO3	-8.7
76573	o1c(cc(=O)c2c(c(C)c(c(C)c12)OC)O)c1ccc(cc1)OC	-8.7
94156	O1c2ccccc2CC[C@@H]1c1ccccc1	-8.7
101101153	c1(cc2c(c(=O)cc(o2)c2ccccc2)c(c1OC)O)O[C@@H]1O[C@@H]([C@H]([C@@H]([C@H]1O)O)O)CO	-8.6
101214938	c1(ccc(cc1)N1CCN(CC1)c1ccncc1)c1oc2ccccc2c(=O)c1O	-8.6

101429132	c1(cc(c(c(c1)CC=C(C)C)OC)/C=C/C(C)(C)O)[C@H]1Oc2cc(ccc2C(=O)C1)O	-8.6
102341487	c1(cc(c2c(=O)cc(oc2c1)c1ccc(c(c1)OC)OC)O)O[C@@H]1O[C@@H]([C@H]([C@@H]([C@@H]1O)O)O)C	-8.6
10901472	o1c2ccccc2c(=O)c(c1c1ccc(cc1)C=C)O	-8.6
11995581	c1(cc(c(c(c1)CC=C(C)C)OC)/C=C/C(C)(C)O)[C@H]1Oc2cc(ccc2C(=O)C1)O	-8.6
1232441	O1c2ccccc2CC[C@H]1c1ccccc1	-8.6
12753176	[C@H]1(C(=C)C(=O)c2ccccc2O1)c1c(Cl)cc(cc1)Cl	-8.6
129711278	c1(cccc2oc(cc(=O)c12)c1ccccc1)C(O)(O)[C@@H](C(C)C)O	-8.6
132472006	o1c2cc(C)cc(c2c(=O)c(C)c1c1ccc(C)cc1)O	-8.6
139593877	o1c(cc(=O)c2cc(cc(c12)OC)CC(=O)C)c1ccc(cc1)OC	-8.6
14134114	o1c(c(c(=O)c2c(cc(c(c12)C(=O)C[C@@H](C)C(=O)O)O)O)O)c1ccc(cc1)O	-8.6
14254494	o1cc(c(=O)c2c(C)cc(cc12)O)c1ccccc1	-8.6
145726	o1c2ccccc2c(=O)cc1c1ccc(c(c1)O)O	-8.6
160237	o1c(cc(=O)c2c(c(c(cc12)OC)OC)O)c1ccc(c(c1)O)O	-8.6
20489	o1c(cc(=O)c2c(cc(c(c12)O)OC)O)c1ccccc1	-8.6
21325037	c1(ccc2c(c(=O)cc(o2)c2ccccc2OC(C)C)c1)C(=O)Cl	-8.6
21604819	O1c2c(c(ccc2CC[C@H]1c1ccc(cc1)O)O)CC=C(C)C	-8.6
246330	O1c2cc(ccc2C(=O)[C@@H]([C@@H]1c1ccc(c(c1)O)O)O)O	-8.6
25022738	O1c2cc(c(cc2C(=O)C[C@H]1c1ccccc1)OC)O	-8.6
25058575	c1(cc(c(cc1OC)OCC=C(C)C)OC)c1coc2c(c1=O)c(cc(c2)OC)OC	-8.6
25201487	O1[C@@H](C(=O)C(=O)c2ccccc12)c1ccccc1	-8.6
368925	O1c2c(c(cc(c2C(=O)[C@@H]([C@@H]1c1ccc(cc1)O)O)O)O)CC=C(C)C	-8.6
373261	O1c2cc(cc(c2C(=O)C[C@@H]1c1ccc(c(c1)O)O)O)O	-8.6

4169706	Fc1ccc2c(C(=O)C[C@@H](O2)c2ccccc2)c1	-8.6
42608069	O1c2c(c(cc3OC(C)(C)C=Cc23)OC)C(=O)C[C@H]1c1ccc(c(c1)OC)OC	-8.6
42608071	O1c2c(c(cc(c2C(=O)C[C@H]1c1ccc(cc1)O)O)OC)CC=C(C)C	-8.6
44257359	o1cc(c(=O)c2c(c3c(cc12)OCO3)OC)c1ccc(c(c1)O)OC	-8.6
44258651	o1c(cc(=O)c2c(c(c(c(c12)OC)O)OC)O)c1ccc2c(OCO2)c1	-8.6
44259965	o1c(c(c(=O)c2c(cc(c(c12)O)OC)O)O)c1ccc(cc1)O	-8.6
44260022	o1c(c(c(=O)c2c(cc(c(c12)OC)OCC=C(C)C)O)OC)c1ccc(c(c1)OC)O	-8.6
44260056	o1c(cc(=O)c2c(c(c(c(c12)OC)OC)OC(=O)C(C)C)O)c1ccc(cc1)O	-8.6
455313	o1c2ccccc2c(=O)c(c1c1ccccc1O)O	-8.6
462699	O1c2ccc(cc2C(=O)C[C@H]1c1ccc(cc1)O)O	-8.6
468911	O1c2c(c(cc(c2C(=O)[C@@H]([C@H]1c1ccc(cc1)O)O)O)O)O	-8.6
49676	o1c(c(C)c(=O)c2ccc(c(c12)CN(CC)CC)OC)c1ccccc1	-8.6
509245	O1c2c(c(cc(c2C(=O)C[C@H]1c1ccc(cc1)O)O)O)CC=C(C)C	-8.6
5272801	o1c2ccccc2c(=O)cc1c1ccc(cc1)C1=NCCO1	-8.6
5280681	o1c2cc(cc(c2c(=O)c(c1c1ccc(c(c1)O)O)OC)O)O	-8.6
5280699	o1c(c(c(=O)c2c(c(c(cc12)OC)OC)O)OC)c1ccc(c(c1)O)O	-8.6
5281603	o1c(c(c(=O)c2c(c(c(cc12)O)OC)O)OC)c1ccc(c(c1)O)O	-8.6
5281614	o1c2cc(ccc2c(=O)c(c1c1ccc(c(c1)O)O)O)O	-8.6
5281670	o1c2cc(cc(c2c(=O)c(c1c1ccc(cc1O)O)O)O)O	-8.6
5281674	o1c(cc(=O)c2c(cc(c(c12)O)O)O)c1ccccc1	-8.6
5316653	o1cc(c(=O)c2c(c3c(cc12)OCO3)O)c1cc(c(c(c1)OC)OC)O	-8.6
5316900	o1c2cc(cc(c2c(=O)c(c1c1ccc(c(c1)OC)O)OC)O)O	-8.6
5322065	o1c2cc(ccc2c(=O)cc1c1ccc(c(c1)O)O)O	-8.6
5378171	o1c(cc(=O)c2c(cc(C)cc12)O)c1ccc(cc1)OC	-8.6
5378210	o1c(cc(=O)c2c(cc(cc12)O)O)c1ccccc1OC	-8.6

54553686	O1c2ccccc2C(=O)C[C@H]1c1cccc(c1)C#N	-8.6
5487268	c1(cc(c(cc1C(C)(C)C=C)c1coc2cc(cc(c2c1=O)O)O)O)O	-8.6
55748	o1c(cc(=O)c2cccc(c12)CC(=O)O)c1ccccc1	-8.6
57340185	c1(cc(=O)c2ccc(cc2o1)O)c1ccc(I)cc1	-8.6
578729	o1c2ccccc2c(=O)c(c1c1ccccc1OC)O	-8.6
6028923	c1(cc(=O)c2ccc(cc2o1)O)c1ccc(Br)cc1	-8.6
66650373	c1(cccc2oc(cc(=O)c12)c1ccccc1)CBr	-8.6
676033	o1c2ccccc2c(=O)cc1c1cccc(c1OC)OC	-8.6
676299	o1c(cc(=O)c2ccc(c(c12)O)OC)c1ccccc1	-8.6
68112	o1c(cc(=O)c2c(cccc12)O)c1ccccc1	-8.6
688672	o1c(cc(=O)c2ccc(cc12)OC)c1cccc(c1)OC	-8.6
69029207	c1(ccc2c(=O)cc(oc2c1)c1ccc(c(c1)OC)OC)OCCCBr	-8.6
69279473	O1c2ccc(cc2C(=O)[C@H]([C@H]1c1ccccc1)O)CCCC	-8.6
70350763	O1c2ccccc2C(=O)/C(=C\O)/[C@H]1c1ccccc1	-8.6
71588336	O1c2cc(c3c(=O)cc(oc3c2[C@H]([C@@H](C1(C)C)O)O)c1ccc(cc1)O)O	-8.6
71621986	c1(cc2c(c(=O)c(co2)c2ccc(cc2)O)cc1OC)O[C@@H]1O[C@@H]([C@H]([C@@H]([C@H]1O)O)OC)CO	-8.6
73829902	O1c2c(c(ccc2C(=O)[C@H]([C@@H]1c1ccc(c(c1)O)O)O)O)O	-8.6
86236611	c1(ccc2c(=O)cc(oc2c1)c1ccc(c(c1)OC)OC)OC[C@@H]1OC1	-8.6
89858365	o1c(c(c(=O)c2c(cccc12)C(=O)C)O)c1ccccc1	-8.6
9972910	o1c(c(c(=O)c2c(cc(c(c12)OC)O)O)OC)c1ccccc1	-8.6
10022760	c1(cc(c2c(=O)c(c(oc2c1)c1ccc(c(c1)O)O)O)O)OS(=O)(=O)O	-8.5
101245387	c1(c(cc(c2c(=O)c(c(oc12)c1ccc(cc1)OC)O)O)O)OC[C@H]1O[C@@H]1c1ccc(c(c1)O)O	-8.5
101685251	c1(ccc(cc1)C(=O)NCCO)c1oc2ccccc2c(=O)c1	-8.5
10174053	c1(cc2c(c(=O)cc(o2)c2ccccc2)c(c1OC)O)OS(=O)(=O)O	-8.5

8		
102145698	O1c2cc(c(cc2C(=O)C[C@@H]1c1ccc(c(c1)O)O)O)OC	-8.5
102928	O1c2ccccc2C(=O)C[C@H]1c1ccc(cc1)OC	-8.5
10598514	O1[C@@H](CC(=O)c2c(cc(c(c12)C[C@H](C(=C)C)CCC(C)(C)O)O)O)c1ccccc1O	-8.5
10685123	c1(ccc2c(c(=O)cc(o2)c2ccccc2)c1)CN=C=S	-8.5
10902653	c1(cc(=O)c2ccccc2o1)c1ccc(cc1)S(=O)(=O)C	-8.5
11382711	c1(c(cc2c(c(=O)c(co2)c2ccc(cc2)O)c1O)O)CCC(C)(C)O	-8.5
12088189	o1c2ccccc2c(=O)c(c1c1ccc(cc1)N(CC)CC)O	-8.5
12098358	O1c2c(c(cc(c2C(=O)C[C@H]1c1ccccc1O)O)OC)OC	-8.5
126392	o1c(cc(=O)c2cccc(c12)CC(=O)OCCN(C)C)c1ccccc1	-8.5
12674946	o1c2ccccc2c(=O)c(c1c1ccccc1)C(=O)O	-8.5
12735893	o1c(c(c(=O)c2ccc3c(cco3)c12)OC)c1ccc(cc1)OC	-8.5
12753167	[C@H]1(C(=C)C(=O)c2ccccc2O1)c1ccc(Cl)cc1	-8.5
129394	O1c2c(c(c(C)c(c2[C@@H](C[C@H]1c1ccccc1)O)OC)O)C=O	-8.5
129670476	O1c2c(C)ccc(c2C(=O)C[C@H]1c1ccccc1)CO	-8.5
129822361	o1c(cc(=O)c2cc(c(c(c12)N)OC(=O)C)OC(=O)C)c1ccccc1	-8.5
129822413	o1c(cc(=O)c2cc(cc(c12)N)OC(=O)C)c1ccc(cc1)OC(=O)C	-8.5
129864635	o1c(cc(=O)c2c(cccc12)OCO)c1ccccc1	-8.5
12988090	c1(ccc2c(=O)cc(oc2c1)c1ccccc1)OP(=O)(OCC)OCC	-8.5
13889022	o1c(cc(=O)c2c(cc(c(c12)OC)OC)O)c1c(cccc1OC)O	-8.5
139593878	o1c(cc(=O)c2cc(cc(c12)OC)CCCO)c1ccc(cc1)OC	-8.5

14885875	O1c2cc(cc(c2CC[C@H]1c1ccccc1)OC)O	-8.5
16681753	o1c(cc(=O)c2c(c(c(cc12)OC)OC)O)c1cc(c(c(c1)OC)O)OC	-8.5
177032	O1c2cc(c(c(c2C(=O)C[C@H]1c1ccccc1)O)OC)O	-8.5
182026	O1c2cc(c(C)c(c2C(=O)[C@@H]([C@H]1c1ccc(c(c1)O)O)O)O)O	-8.5
195603	O1c2cc(cc(c2C(=O)C[C@H]1c1c(cccc1O)O)O)OC	-8.5
21325058	O1c2ccc(cc2C(=O)C[C@@H]1c1ccccc1N(=O)=O)C(=O)OC	-8.5
227445	o1c(c(c(=O)c2cc(C)ccc12)O)c1ccccc1	-8.5
265699	o1c(c(c(=O)c2ccc(cc12)OC)O)c1ccc(cc1)OC	-8.5
342294	O1c2cc(cc(c2CC[C@H]1c1ccccc1)OC)O	-8.5
344546	o1c(c(c(=O)c2ccc(cc12)OC)O)c1ccccc1	-8.5
348130	O1c2cc(cc(c2C(=O)C[C@H]1c1ccc(cc1)O)O)OC	-8.5
369608	O1c2c(cc(cc2[C@H](C[C@@H]1c1ccccc1)O)CC=C)OC	-8.5
4231835	o1c2ccccc2c(=O)cc1c1cc(ccc1OC)OC	-8.5
42608063	O1c2c(c(cc3OC(C)(C)C=Cc23)OC)C(=O)C[C@H]1c1ccc(cc1)OC	-8.5
4303567	o1c(cc(=O)c2c(cc(cc12)OC(=O)C)OC(=O)C)c1ccc(cc1)OC	-8.5
439712	O1c2ccccc2[C@H](C[C@H]1c1ccccc1)O	-8.5
44258621	O1c2c3c(=O)cc(oc3c(c(c2C=CC1(C)C)OC)OC)c1cc(c(c(c1)OC)OC)OC	-8.5
462700	O1c2ccccc2C(=O)C[C@H]1c1ccc(c(c1)O)O	-8.5
4872981	O1c2cc(cc(c2C(=O)C[C@H]1c1ccc(c(c1)O)O)O)OC	-8.5
491719	O1c2c(c(c(C)c(c2C(=O)C[C@H]1c1ccc(cc1)O)O)O)CC=C(C)C	-8.5
49788611	o1c(c(c(=O)c2c(cc(c(c12)OC)OC)O)O)c1ccc2c(OCO2)c1	-8.5
51459	o1c2ccccc2c(=O)c(C)c1c1ccccc1	-8.5
5271551	O1c2cc(cc(c2C(=O)C[C@H]1c1ccc(cc1)O)OC)OC	-8.5
5281666	o1c(c(c(=O)c2c(cc(cc12)O)O)O)c1ccc(cc1)OC	-8.5
5281797	o1cc(c(=O)c2c(c(c(cc12)O)CC=C(C)C)O)c1ccc(cc1O)O	-8.5

5317284	o1c(cc(=O)c2c(c(c(cc12)O)OC)O)c1ccc(c(c1)O)O	-8.5
5317747	O1c2cc(cc(c2C(=O)[C@H]([C@@H]1c1ccccc1)O)O)OC	-8.5
5321861	o1c(c(c(=O)c2c(cc(c(c12)O)O)O)OC)c1ccc(c(c1)O)OC	-8.5
5378202	c1(c(cc(c2c(=O)c(coc12)c1ccc(cc1)O)O)O)CCC(C)(C)O	-8.5
53947837	O1c2ccc(C)cc2CC[C@H]1c1ccc(cc1)OC	-8.5
5469524	o1c(cc(=O)c2c(c(c(cc12)O)OC)O)c1ccc(c(c1)O)OC	-8.5
57363368	o1c(c(c(=O)c2cc(cc(c12)O)O)O)c1ccc(cc1)OC	-8.5
619834	o1c2ccccc2c(=O)cc1c1cccc(c1)OC	-8.5
6253276	c1(c(c(cc(c1C(=O)/C=C/c1ccc(cc1)OC)O)OC)OC)OC	-8.5
66916672	o1c2ccccc2c(=O)c(c1C(=O)C)c1ccccc1	-8.5
67424417	c1(ccc(cc1)[C@@H]1CC(=O)c2ccccc2O1)S(=O)(=O)C(F)(F)F	-8.5
688671	o1c(cc(=O)c2ccc(cc12)OC)c1ccccc1OC	-8.5
688715	o1c2ccccc2c(=O)c(c1c1ccc(cc1)O)O	-8.5
688853	o1c(cc(=O)c2ccc(c(c12)O)O)c1ccc(cc1)O	-8.5
689014	o1c(cc(=O)c2ccc(c(c12)OC)OC)c1ccccc1	-8.5
69030155	c1(ccc2c(=O)cc(oc2c1)c1cc(cc(c1)OC)OC)OCCCBr	-8.5
71588337	O1c2cc(c3c(=O)cc(oc3c2[C@@H]([C@H](C1(C)C)O)OCC)c1ccc(cc1)O)O	-8.5
72304	o1cc(c(=O)c2ccccc12)c1ccccc1	-8.5
7330537	O1c2ccc3c(=O)c(coc3c2C=CC1(C)C)c1cc(c2c(OCO2)c1)OC	-8.5
73571	O1c2cc(cc(c2C(=O)C[C@H]1c1ccc(cc1)O)O)OC	-8.5
76852307	o1c(cc(=O)c2ccc(c(c12)C=O)O)c1ccccc1	-8.5
97214	o1c(cc(=O)c2c(c(c(cc12)OC)OC)O)c1ccc(c(c1)O)OC	-8.5
10018499	O1c2c(c(c(C)c(c2C(=O)C[C@]1(c1ccccc1)O)O)O)C=O	-8.4
10106405<br>5	c1(coc2ccccc2c1=O)c1ccc(cc1)C(F)(F)F	-8.4
10113705	c1(c(cc(c2c(=O)c(coc12)c1ccc(cc1O)O)O)O)CC(C)(C)O	-8.4

4		
101367767	O1c2c(c(cc(c2C(=O)C[C@H]1c1ccc(cc1)O)O)O)C/C=C(\C)/C=O	-8.4
10185	O1c2cc(cc(c2C(=O)[C@H]([C@H]1c1ccc(c(c1)O)O)O)O)O	-8.4
10832934	O1c2ccc(cc2[C@H]([C@@H](C1(C)C)O)O)c1coc2cc(cc(c2c1=O)O)O	-8.4
11033582	O1c2c(c(ccc2C[C@@H]([C@H]1c1ccc(c(c1)O)O)O)O)O	-8.4
11055	o1c(cc(=O)c2c(ccc(c12)O)O)c1ccccc1	-8.4
11810419	O1c2c(c(cc(c2C(=O)C[C@@H]1c1ccc(cc1O)O)O)O)CC=C(C)C	-8.4
12052874	O1c2cc(ccc2CC[C@H]1c1ccc2c(OCO2)c1)O	-8.4
12318031	O1c2ccccc2C[C@H]([C@H]1c1ccccc1)O	-8.4
12360936	O1c2cc(ccc2[C@@H](C[C@H]1c1ccccc1)O)OC	-8.4
125526	O1c2cc(cc(c2C(=O)[C@@H](C)[C@H]1c1ccc(cc1)O)O)O	-8.4
129684435	O1c2ccccc2C[C@H]([C@H]1c1ccccc1)O	-8.4
129819557	O1c2ccccc2C(=O)[C@H]([C@H]1c1ccccc1)O	-8.4
129847959	O1c2c(c(ccc2[C@H]([C@@H]([C@H]1c1ccc(cc1)O)O)O)O)O	-8.4
129858370	o1c2cc(ccc2c(=O)cc1c1cc(c(c(c1)OC)OC)OC)OCC(=O)O	-8.4
129881027	o1cc(c(=O)c2ccc(c(c12)CO)O)c1cc(c(c(c1)CO)O)CO	-8.4
14034216	o1c(c(c(=O)c2c(cc(c(c12)OC)OC)O)O)c1ccc(c(c1)OC)OC	-8.4
15301053	o1cc(c(=O)c2c(cc(cc12)O)O)c1ccc2c(OCO2)c1	-8.4
153441	o1c(cc(=O)c2c(c(c(c(c12)O)OC)OC)O)c1ccccc1	-8.4
15389726	c1(cc(=O)c2ccccc2o1)c1ccc(cc1)S(=O)(=O)Cl	-8.4
15389730	c1(cccc(c1)c1cc(=O)c2ccccc2o1)S(=O)(=O)N(CC)CC	-8.4

15458309	O1c2c(c(ccc2C(=O)C[C@H]1c1ccc(cc1)OC)OC)OC	-8.4
158311	o1c(cc(=O)c2c(c(c(cc12)OC)OC)O)c1cc(c(cc1O)O)OC	-8.4
161271	o1c(cc(=O)c2c(c(c(cc12)OC)OC)O)c1ccc(cc1)OC	-8.4
162464	o1c(cc(=O)c2c(c(c(cc12)OC)OC)O)c1ccc(c(c1)OC)O	-8.4
1666397	o1c2ccccc2c(=O)c(c1c1ccccc1)OC(=O)C	-8.4
1880	o1c(cc(=O)c2ccc(c(c12)O)O)c1ccccc1	-8.4
194690	o1c(cc(=O)c2cc(cc(c12)OC)OC)c1ccccc1	-8.4
20301086	O1c2ccccc2C(=O)C(=C)[C@@H]1c1cccc(c1)N(=O)=O	-8.4
20394006	o1c(C)c(c(=O)c2c(cc(cc12)OC(=O)C)OC(=O)C)c1ccccc1	-8.4
21270134	c1(coc2cc(c(cc2c1=O)O)O)c1cccc(Cl)c1	-8.4
21270152	o1cc(c(=O)c2ccc(c(C)c12)O)c1ccc(cc1)N(=O)=O	-8.4
213728	o1c(cc(=O)c2cccc(c12)OC)c1ccccc1	-8.4
226342	O1c2ccccc2C(=O)C[C@H]1c1ccc(c(c1)OC)O	-8.4
227443	o1c(c(c(=O)c2cc(C)ccc12)O)c1ccc(cc1)OC	-8.4
24205532	c1(cc(c2c(=O)c(c(oc2c1)c1ccc(cc1)O)CC=C(C)C)OC)O[C@H]1O[C@H]([C@@H]([C@H]([C@H]1O)O)O)C	-8.4
26034	o1c(c(c(=O)c2c(cc(cc12)OC)OC)O)c1ccc(c(c1)O)O	-8.4
3082330	O1c2cc(cc(c2C(=O)[C@@H]([C@H]1c1ccccc1O)O)O)O	-8.4
31161	o1c(cc(=O)c2c(c(c(cc12)OC)O)O)c1ccc(c(c1)O)O	-8.4
321347	O1c2cc(cc(c2C(=O)C[C@H]1c1ccc(c(c1)OC)O)O)OC	-8.4
3707243	O1c2ccccc2C[C@H]([C@H]1c1ccccc1)O	-8.4
373260	o1c(cc(=O)c2c(c(c(cc12)OC)O)O)c1ccc(c(c1)O)OC	-8.4
3826106	O1c2ccccc2[C@H](CC1)c1ccccc1	-8.4
42607939	O1C(C)(C)C=Cc2c(c3C(=O)C[C@H](Oc3c(c12)C[C@@H](C(=C)C)O)c1ccc(cc1)O)O	-8.4
439533	O1c2cc(cc(c2C(=O)[C@@H]([C@H]1c1ccc(c(c1)O)O)O)O)O	-8.4
44258614	O1c2c3c(=O)cc(oc3c(c(c2C=CC1(C)C)OC)OC)c1ccc(c(c1)OC)OC	-8.4

442621	o1c(c(c(=O)c2c(c(c(cc12)OC)O)O)OC)c1ccc(c(c1)O)OC	-8.4
443638	O1c2cc(cc(c2[C@H](C[C@H]1c1ccc(cc1)O)O)O)O	-8.4
466078	O1c2cc(ccc2CC[C@H]1c1ccc2c(OCO2)c1)O	-8.4
466293	Clc1ccc2c(c(=O)c(c(o2)c2ccc(cc2)OC)O)c1	-8.4
5106787	O1c2ccc(cc2C(=O)C[C@H]1c1ccccc1O)O	-8.4
513103	O1c2ccccc2C(=O)[C@H](C1)Cc1ccccc1	-8.4
5272799	O1c2ccccc2C(=O)C[C@H]1c1ccc(cc1)C1=NCCO1	-8.4
5273755	o1c(cc(=O)c2c(c(c(cc12)O)OC)O)c1ccc(c(c1)OC)OC	-8.4
5280417	o1c(c(c(=O)c2c(cc(cc12)OC)O)OC)c1ccc(c(c1)O)O	-8.4
5280445	o1c(cc(=O)c2c(cc(cc12)O)O)c1ccc(c(c1)O)O	-8.4
5281612	o1c2cc(cc(c2c(=O)cc1c1ccc(c(c1)O)OC)O)O	-8.4
52951513	O1c2cc3c(c(=O)c(co3)c3ccc(c(c3)OC)OC[C@H](C(C)(C)O)O)c(c2C=CC1(C)C)OC	-8.4
5317435	O1c2cc(ccc2C(=O)[C@@H]([C@H]1c1ccc(c(c1)O)O)O)O	-8.4
5318869	o1c(c(c(=O)c2c(cc(cc12)OC)O)OC)c1ccc(cc1)O	-8.4
5320181	o1c(cc(=O)c2c(c(c(cc12)O)O)O)c1ccc(c(c1)OC)O	-8.4
5320693	o1c2cc(ccc2c(=O)cc1c1ccc(cc1)OC)O	-8.4
53247999	c1(ccc(cc1)[C@H]1CC(=O)c2ccccc2O1)O[C@H]1O[C@@H]([C@H]([C@H]([C@H]1O)O)O)C	-8.4
53398699	c1(ccc2c(=O)c(coc2c1)c1ccc(cc1)O)O[C@@H]1O[C@H]([C@@H]([C@H]([C@H]1O)O)O)COC(=O)C	-8.4
53645	o1c(cc(=O)c2cccc(c12)O)c1ccccc1	-8.4
5377381	o1cc(c(=O)c2c(cc(cc12)O)O)c1ccccc1	-8.4
5378518	o1c2cc(ccc2c(=O)cc1c1ccc(c(c1)OC)OC)O	-8.4
5383438	o1c2cc(cc(c2c(=O)c(c1c1ccc(c(c1)OC)OC)OC)O)O	-8.4
5407860	o1cc(c(=O)c2ccc(c(c12)O)O)c1ccccc1	-8.4
54512651	O1c2ccccc2C(=O)/C(=C\N)/[C@H]1c1ccccc1	-8.4
55750	c1(cccc2c(=O)cc(oc12)c1ccccc1)CC(=O)OCCN(CC)CC	-8.4

5748553	o1c(c(c(=O)c2c(cc(c(c12)OC)O)O)OC)c1ccc(c(c1)O)O	-8.4
57679080	O1c2ccccc2CC[C@H]1c1ccc(c(c1)O)O	-8.4
586090	O1c2cccc(c2C(=O)C[C@H]1c1ccccc1)O	-8.4
59059924	o1cc(c(=O)c2ccc(cc12)OC(=O)C(=C)C)c1ccccc1	-8.4
631171	o1c2cc(ccc2c(=O)c(c1c1ccc(c(c1)OC)OC)OC)OC	-8.4
66968868	c1(ccc2c(c(=O)c(c(o2)c2ccc(cc2)N(CC)CC)O)c1)CBr	-8.4
67425485	c1(cc(c2c(=O)cc(oc2c1)c1ccc(c(c1)OC)OC)OC)OCP(=O)(C(C)C)C(C)C	-8.4
69030799	c1(ccc2c(=O)cc(oc2c1)c1cc(c(c(c1)OC)OC)OC)OCCCBr	-8.4
70018578	O1c2cc(ccc2C(=O)C[C@@H]1c1ccc(cc1)OC)O	-8.4
70253991	o1c(c(c(=O)c2c(c(c(c(c12)O)OC)OC)O)O)c1ccccc1	-8.4
73265447	c1(coc2c(C)c(ccc2c1=O)O)c1ccc(cc1)C(F)(F)F	-8.4
10108482	c1(ccc2c(=O)c(coc2c1)c1ccccc1)OC[C@H]1OC1	-8.3
101367766	O1c2c(c(cc(c2C(=O)C[C@H]1c1ccc(cc1)O)O)O)C/C=C(\C)/CO	-8.3
101591998	c1(cc(c2c(=O)cc(oc2c1OC)c1ccccc1)OC)O[C@@H]1O[C@@H]([C@H]([C@@H]([C@H]1O)O)O)CO	-8.3
101685249	c1(ccc(cc1)C(=O)NCCO)[C@H]1Oc2ccccc2C(=O)C1	-8.3
10422310	o1c(cc(=O)c2c(cccc12)N)c1ccc(cc1)N	-8.3
10904774	c1(c(cc(c2c(=O)c(coc12)c1ccc(cc1O)O)O)O)CCC(C)(C)O	-8.3
11111496	O1c2c(c(cc(c2C(=O)C[C@H]1c1ccc(c(c1)O)O)O)O)C/C=C(\C)/CO	-8.3
11244593	O1c2cc(c(cc2C=CC1(C)C)c1coc2cc(ccc2c1=O)O)O	-8.3
11349	o1c2ccccc2c(=O)c(c1c1ccccc1)O	-8.3
11507421	o1c2ccccc2c(=O)cc1c1ccc(cc1)OC(=O)C	-8.3
11723752	o1cc(c(=O)c2c(C)cc(cc12)OC(=O)OCC)c1ccccc1	-8.3
11739635	c1(cc(c(c(c1)CC=C(C)C)OC)O)c1coc2c(c1=O)ccc(c2)O	-8.3

117900	o1c(c(c(=O)c2c(cc(cc12)OC)OC)OC)c1ccccc1	-8.3
12444947	c1(cc2c(c(=O)c(co2)c2ccc(cc2)OC)cc1OC)O[C@@H]1O[C@H]([C@@H]([C@H]([C@H]1O)O)O)CO	-8.3
12578908	O1c2cc(cc(c2C(=O)[C@H](C1)c1ccccc1)O)O	-8.3
129711222	O1C2=C(CC(=O)C=C2)CC[C@@H]1c1ccccc1	-8.3
129847909	o1c(c(c(=O)c2ccc(cc12)OC(O)O)OCO)c1ccccc1	-8.3
129864294	O1c2c(cccc2C(=O)C[C@H]1c1ccc(cc1)O)OC	-8.3
129882404	o1cc(c(=O)c2ccccc12)c1ccc(cc1)CN	-8.3
13250353	O1c2cccc(c2CC[C@H]1c1ccccc1)O	-8.3
132536158	o1c(cc(=O)c2c(cc(cc12)OC(=O)C=C)OC(=O)C=C)c1ccc(cc1)OC(=O)C=C	-8.3
134822278	o1cc(c(=O)c2c(c3c(cc12)OCO3)O)C1=CCC(=CC1)OC	-8.3
13942548	o1c(c(c(=O)c2c(c3c(c(c12)OC)OCO3)OC)OC)c1ccc2c(OCO2)c1	-8.3
13964544	o1c(cc(=O)c2c(cc(cc12)O)OC)c1ccc(c(c1)OC)OC	-8.3
147157	o1c(cc(=O)c2cc(ccc12)OC)c1ccccc1	-8.3
15385487	O1c2cc(ccc2C(=O)C[C@H]1c1ccc(c(c1)OC)O)O	-8.3
154227	o1c2cc(ccc2c(=O)cc1c1ccc(c(c1)OC)OC)OC	-8.3
1546800	O1c2ccccc2C(=O)[C@@H]([C@@H]1c1ccccc1)O	-8.3
15896301	o1c(cc(=O)c2c(c(c(c(c12)OC)OC)OC)O)c1cccc(c1O)OC	-8.3
177000	o1c2cc(ccc2c(=O)c(C)c1c1cc(c(c(c1)OC)OC)OC)OC	-8.3
18702583	o1c(cc(=O)c2c(c(ccc12)OC)O)c1ccc(c(c1)O)O	-8.3
188323	o1c(cc(=O)c2c(c(c(cc12)OC)OC)O)c1ccc(cc1)O	-8.3
21315732	o1c2ccc(cc2c(=O)cc1c1cc(C)ccc1OC(C)C)C(=O)OCC	-8.3

21576250	o1cc(c(=O)c2c(cc(c(c12)C(C)(C)C=C)O)O)c1ccc(cc1O)O	-8.3
23644935	o1c2cc(ccc2c(=O)c(c1n1cncn1)c1ccccc1)OC	-8.3
242486	O1c2cc(c(c(c2C(=O)C[C@H]1c1ccc(cc1)OC)OC)OC)OC	-8.3
24866262	c1(ccc(cc1OC)c1cc(=O)c2c(cc(cc2o1)O)O)O[C@H]1O[C@@H]([C@H]([C@H]([C@H]1O)O)O)C	-8.3
24866263	c1(ccc(cc1)c1cc(=O)c2c(cc(cc2o1)O)O)O[C@H]1O[C@@H]([C@H]([C@H]([C@H]1O)O)O)C	-8.3
253959	O1c2ccccc2[C@H](C[C@@H]1c1ccccc1)O	-8.3
2734290	o1cc(c(=O)c2c(C)cc(cc12)OC)c1ccccc1	-8.3
27443	o1c(c(C)c(=O)c2cc(ccc12)CN(CC)CC)c1ccccc1	-8.3
3062797	o1cc(c(=O)c2ccc(cc12)O[C@@H](C)C(=O)O)c1ccccc1	-8.3
3593	O1c2cc(cc(c2C(=O)C[C@H]1c1ccc(c(c1)O)OC)O)O	-8.3
3890073	N([C@@H]1c2ccccc2O[C@@H](C1)c1ccccc1)NC(=S)N	-8.3
462695	O1c2cc(cc(c2C(=O)[C@H]([C@@H]1c1ccc(c(c1)OC)OC)O)OC)OC	-8.3
471	O1c2cc(cc(c2C(=O)[C@@H]([C@@H]1c1ccc(c(c1)O)O)O)O)O	-8.3
5049148	O1c2c(c(c3c(=O)c(coc3c2C=CC1(C)C)c1ccc2c(OCO2)c1)OC)OC	-8.3
5280343	o1c(c(c(=O)c2c(cc(cc12)O)O)O)c1ccc(c(c1)O)O	-8.3
5280666	o1c2cc(cc(c2c(=O)cc1c1ccc(c(c1)OC)O)O)O	-8.3
5281628	o1c(cc(=O)c2c(c(c(cc12)O)OC)O)c1ccc(cc1)O	-8.3
5281702	o1c2cc(cc(c2c(=O)cc1c1cc(c(c(c1)OC)O)OC)O)O	-8.3
5281805	c1(coc2cc(ccc2c1=O)O)c1ccc2OCOc2c1	-8.3
5316733	O1c2cc(c(c(c2C(=O)C[C@H]1c1ccccc1)O)OC)O	-8.3
5320351	o1c(c(c(=O)c2c(c(c(cc12)OC)OC)O)OC)c1ccc(c(c1)OC)OC	-8.3
5320438	o1c(cc(=O)c2c(c(c(cc12)O)OC)O)c1ccc(cc1)OC	-8.3
5321538	O1c2cc(c(cc2C(=O)C[C@@H]1c1ccc(c(c1)O)O)OC)O	-8.3
5321987	[C@@H]1(c2cc(ccc2OC[C@@H]1c1cc(cc(c1O)OC)/C=C/C(=O)O)OC)OS(=O)(=O)O	-8.3
53247946	c1(ccc2c(C(=O)C[C@H](O2)c2ccc(cc2)O)c1)O[C@@H]1O[C@@H]([C@H]([C@@H]([C@H]1O)O)OC)CO	-8.3

5352005	o1c2cc(cc(c2c(=O)c(c1c1ccc(c(c1)OC)OC)OC)O)OC	-8.3
5352032	o1c(c(c(=O)c2c(c(c(cc12)O)OC)O)OC)c1ccc(cc1)O	-8.3
5380976	o1c2cc(ccc2c(=O)c(c1C)c1ccccc1)O	-8.3
5381919	o1c(cc(=O)c2c(cc(C)cc12)O)c1cc(c(c(c1)OC)OC)OC	-8.3
5384810	o1c(c(c(=O)c2c(cc(c(c12)OC(=O)CCC)OC)O)O)c1ccc(cc1)OC	-8.3
5395695	o1c(C)c(c(=O)c2c(cc(cc12)O)O)c1ccccc1	-8.3
5459184	o1c(c(c(=O)c2c(cc(c(c12)OC)OC)O)OC)c1ccc(c(c1)OC)O	-8.3
54799	o1c(c(c(=O)c2c(c(c(c(c12)OC)OC)OC)O)OC)c1ccc(c(c1)O)O	-8.3
56648913	c1(cc2c(c(=O)cc(o2)c2ccccc2)c(c1OC(=O)C)OC(=O)C)OCc1ccc(F)cc1	-8.3
57253387	O1c2ccccc2[C@H](C[C@H]1c1ccccc1)NNC(=O)N	-8.3
624831	o1c(c(c(=O)c2c(cc(cc12)OC)OC)O)c1ccc(cc1)OC	-8.3
636642	Clc1c(cc2c(C[C@@H]([C@H](O2)c2ccc(c(c2)O)O)O)c1O)O	-8.3
6399103	O1c2ccccc2C(=O)[C@@H](NO)[C@H]1c1ccccc1	-8.3
67114276	o1c(c(c(=O)c2c(cccc12)C(=O)C)C(=O)C)c1ccccc1	-8.3
71762131	o1c(cc(=O)c2ccc(c(c12)OC)O)c1ccccc1	-8.3
72281	O1c2cc(cc(c2C(=O)C[C@H]1c1ccc(c(c1)O)OC)O)O	-8.3
77793	o1c2ccccc2c(=O)cc1c1ccc(cc1)OC	-8.3
86606987	c1(ccc2c(=O)cc(oc2c1)c1cc(c(c(c1)OC)OC)OC)OC[C@@H]1OC1	-8.3
86641845	o1c(cc(=O)c2c(cc(cc12)OCC#C)O)c1ccc(c(c1)OC)OC	-8.3
91724402	O1c2cc(cc(c2C(=O)C[C@H]1c1ccc(cc1)OC(=O)C)O)OC	-8.3
97142	o1c(c(c(=O)c2c(cc(cc12)OC)OC)O)c1ccc(c(c1)OC)OC	-8.3
9798295	O1c2cc(c(c(c2C(=O)C[C@H]1c1ccc(c(c1)O)O)OC)OC)OC	-8.3
101932173	c1(c(c(c2C(=O)C[C@H](Oc2c1)c1ccccc1)O)OC)O[C@@H]1O[C@@H]([C@H]([C@@H]([C@H]1O)O)O)CO	-8.2
102577926	o1c(cc(=O)c2c(cc(cc12)O)O)c1ccc(cc1)OCOC	-8.2

10613051	c1(cc(=O)c2ccccc2o1)c1ccc(cc1)[S@](=O)C	-8.2
11316719	o1c(cc(=O)c2c(c(c(cc12)OCc1ccccc1)OC(=O)C)OC(=O)C)c1ccccc1	-8.2
11609345	o1c(cc(=O)c2c(c(c(c(c12)OC)O)OC)O)c1ccccc1	-8.2
12000157	O1c2cc(cc(c2C(=O)C[C@@H]1c1ccc(cc1O)O)O)OC	-8.2
12043066	c1(cc(=O)c2c(cc(c(c2o1)[C@@H]1CCCC[C@@H]1O)O)O)c1c(Cl)cccc1	-8.2
12241083	o1cc(c(=O)c2c(cc(cc12)O)O)c1ccc(cc1)N(=O)=O	-8.2
12407866	o1c(cc(=O)c2cc(ccc12)C(=O)OC)c1ccccc1O	-8.2
1241435	o1c2ccccc2c(=O)cc1c1ccc(cc1)N(C)C	-8.2
129714302	O1c2ccccc2C(=O)[C@H]([C@@]1(c1ccccc1)Cc1ccccc1)O	-8.2
129732270	o1c2ccccc2c(=O)c(c1c1ccccc1)OO	-8.2
129818277	O1c2cc(ccc2[C@H](C(=O)[C@@H]1c1ccccc1)OC)O	-8.2
129844531	O1c2cc(cc(c2C(=O)[C@@](C)([C@H]1c1ccc(c(C)c1)O)O)O)O	-8.2
129858707	O1c2cc(cc(c2C(=O)[C@@]([C@@H]1c1ccc(c(c1)O)O)(OC)O)O)O	-8.2
129887801	o1c2ccc(c(c2c(=O)c(c1c1ccc(c(c1)OC)OC)OC)O)O	-8.2
134820726	o1c(cc(=O)c2cc(cc(c12)OC)OC)c1ccc(cc1)OC	-8.2
134821268	O1[C@@H]2Cc3ccccc3O[C@]12c1ccccc1	-8.2
13568446	o1c2ccccc2c(=O)c(c1c1ccccc1)C(=O)C	-8.2
14130926	o1c(c(c(=O)c2c(cc(cc12)OC(=O)C)OC(=O)C)OC(=O)C)c1ccc(cc1)OC(=O)C	-8.2
14606539	o1c(c(c(=O)c2c(c(c(cc12)OC)OC)OC)O)c1ccccc1	-8.2
18324985	Ic1c(=O)c2ccccc2oc1c1ccc(cc1)S(=O)(=O)C	-8.2

187093	O1c2cc(ccc2C(=O)C[C@H]1c1ccc(c(c1)O)OC)O	-8.2
19358638	o1c(cc(=O)c2c(cc(cc12)OCCO)OC)c1ccc(c(c1)OC)OC	-8.2
2017481	c1(ccc2c(=O)c(coc2c1)c1ccccc1)OS(C)(O)O	-8.2
20394007	c1(ccc2c(=O)c(coc2c1)c1ccccc1)O[C@H](C)CC	-8.2
2064449	O1c2cc(cc(c2C(=O)[C@@H]([C@H]1c1ccc(c(c1)OC)OC)O)OC)OC	-8.2
3070245	c1(c(C)c(=O)c2ccc(c(c2o1)CN(C)C)OC)c1ccc(Cl)cc1	-8.2
315709	o1c(cc(=O)c2cc(c(cc12)OC)OC)c1ccc(c(c1)OC)OC	-8.2
321346	O1c2cc(cc(c2C(=O)C[C@H]1c1ccc(cc1)OC)O)OC	-8.2
343081	o1cc(c(=O)c2ccc3c(OCO3)c12)c1ccc(c(c1)OC)OC	-8.2
369609	O1c2c(cc(cc2[C@H](C[C@H]1c1ccc(c(c1)OC)OC)O)CC=C)OC	-8.2
3825918	o1c2ccccc2c(=O)c(c1c1ccccc1)n1cnnn1	-8.2
42608036	O1[C@@H](CC(=O)c2c(cc(c(c12)C[C@H]([C@H](C)CO)CC=C(C)C)O)O)c1ccc(cc1OC)O	-8.2
44382543	c1(c(cc(c2c(=O)cc(oc12)c1ccccc1)OC)OC)C(F)(F)F	-8.2
471706	o1cc(c(=O)c2cc(c(cc12)OC(=O)C)OC(=O)C)c1ccc(cc1)OC(=O)C	-8.2
5272653	o1c(cc(=O)c2c(cc(cc12)OC)O)c1ccc(c(c1)OC)OC	-8.2
5280442	o1c(cc(=O)c2c(cc(cc12)O)O)c1ccc(cc1)OC	-8.2
5281601	o1c(cc(=O)c2c(cc(cc12)OC)O)c1ccc(cc1)OC	-8.2
5281779	o1cc(c(=O)c2c(c3c(cc12)OCO3)O)c1ccc(cc1)O	-8.2
5351234	o1c2cc(cc(c2c(=O)cc1c1ccc(c(c1)OC)OC)O)O	-8.2
5379262	o1c(c(c(=O)c2c(c(c(c12)OC)OC)OC)O)O)c1ccccc1	-8.2
5386961	o1c(c(c(=O)c2c(cc(c(c12)OC)O)O)OC)c1ccc(c(c1)OC)O	-8.2
5492944	o1cc(c(=O)c2c(cc(c(c12)O)O)O)c1ccc(cc1)O	-8.2
624789	o1cc(c(=O)c2cc(c(cc12)OC)OC)c1ccccc1	-8.2
632255	o1c(cc(=O)c2c(c(c(c12)OC)OC)O)O)c1ccc(c(c1)OC)OC	-8.2
69033169	c1(ccc2c(=O)cc(oc2c1)c1cc(c(c(c1)OC)OC)OC)OCCBr	-8.2

725376	o1c(cc(=O)c2cc(ccc12)OC(=O)C)c1ccc(cc1)OC	-8.2
746742	c1(coc2cc(ccc2c1=O)OC(C)C)c1ccc(F)cc1	-8.2
9562299	N([C@@H]1c2ccccc2O[C@@H](C1)c1ccccc1)NC(=S)N	-8.2
96118	o1c(cc(=O)c2c(c(c(cc12)OC)OC)OC)c1ccc(cc1)OC	-8.2
97332	o1c(c(c(=O)c2c(cc(cc12)OC)OC)OC)c1ccc(c(c1)OC)OC	-8.2
101621206	O1c2ccc(C)cc2C(=O)C[C@H]1c1ccc(cc1)N(C)C	-8.1
101763632	c1(c(cc2c(c(=O)c(co2)c2ccc(cc2)O)c1O)O)CC(C)(C)O	-8.1
102003045	O1c2cc(ccc2C=C(C1)c1cc2c(cc1OC)OCO2)O	-8.1
102320039	o1cc(c(=O)c2c(c(c(c(c12)O)O)OC)O)c1ccc(c(c1)OC)O	-8.1
10475482	o1c(c(c(=O)c2c(c(c(c(c12)OC)OC)OC)O)O)c1ccc(c(c1)OC)O	-8.1
10542374	o1cc(c(=O)c2c(cc(c(c12)O)O)O)c1ccc(c(c1)O)O	-8.1
10643413	Brc1c(=O)c2ccccc2oc1c1ccc(cc1)S(=O)(=O)C	-8.1
10665235	o1c(c(c(=O)c2c(cc(c(c12)OC)OC)O)O)c1ccc(c(c1)O)O	-8.1
10708856	c1(cc(=O)c2ccccc2o1)c1ccc(cc1)CN=C=S	-8.1
10741771	O1c2c(c(c(cc2C[C@@H](C1(C)C)O)[C@@H]1CC(=O)c2c(cc(cc2O1)O)O)CC=C(C)C)O	-8.1
10777811	o1c2ccccc2c(=O)cc1c1ccc(cc1)CC#N	-8.1
10937632	O1c2cc(c(c(c2C(=O)C[C@H]1c1ccc(c(c1)OC)OC)OC)OC)OC	-8.1
11494111	c1(coc2cc(cc(c2c1=O)O)O)c1ccc(c2OC(CCc12)(C)C)O	-8.1
11499970	O1c2ccccc2C(=O)C[C@H]1c1ccc(cc1)N(C)C	-8.1
11709692	o1c(c(c(=O)c2c(cc(cc12)O)OC)O)c1ccc(cc1OC)O	-8.1
11726019	o1c(cc(=O)c2c(c(c(c(c12)O)OC)OC)O)c1ccc(c(c1)OC)O	-8.1
11777425	o1c(cc(=O)c2cc(ccc12)CC#N)c1ccccc1	-8.1

12318033	O1c2ccccc2CC(=O)[C@H]1c1ccccc1	-8.1
124211	o1c(cc(=O)c2c(c(c(c(c12)OC)OC)OC)O)c1c(cccc1OC)O	-8.1
125540	Clc1c(cc(c2C(=O)[C@@H](C)[C@H](Oc12)c1ccc(cc1)O)O)O	-8.1
129735869	O1c2c(cccc2C(=O)C[C@H]1c1ccccc1)/C=C/C(=C)C	-8.1
129781288	O1CC(=C(c2ccccc12)NO)c1ccccc1	-8.1
129829598	o1c(c(c(=O)c2c(c(c(c(c12)O)O)O)OC)O)c1ccccc1	-8.1
129887481	o1c(c(c(=O)c2c(c(ccc12)O)OC)c1ccccc1)OC	-8.1
129887676	O1c2cc(c(cc2C(=O)[C@@H]([C@@H]1c1ccccc1)O)OC)O	-8.1
138402973	o1cc(c(=O)c2cc(c(cc12)CCCO)O)c1ccc(cc1)O	-8.1
13886894	O1c2cc(ccc2[C@H]([C@H]([C@H]1c1ccc(cc1)O)O)O)O	-8.1
14055876	o1c(cc(=O)c2c(c(ccc12)OC)OC)c1ccc(c(c1)OC)OC	-8.1
14440496	o1c2cc(ccc2c(=O)c(c1c1ccccc1OC)O)OC	-8.1
14504257	c1(c(c(cc(c1c1coc2cc(ccc2c1=O)O)O)O)OC)CCC(C)(C)O	-8.1
145659	o1c(cc(=O)c2c(c(c(cc12)OC)OC)OC)c1ccc(c(c1)OC)OC	-8.1
14756305	O1c2cc(cc(c2C(=O)[C@H](C)[C@@H]1c1ccc(cc1)O)O)O	-8.1
1560918	c1(ccc2c(=O)c(coc2c1)c1ccccc1OC)OS(C)(O)O	-8.1
15761521	o1c2ccccc2c(=O)c(c1c1ccccc1)[C@@H](C)C#N	-8.1
160921	o1c(cc(=O)c2c(c(c(c(c12)OC)O)OC)O)c1ccc(cc1)OC	-8.1
185670	o1c(cc(=O)c2c(c(c(cc12)OC)OC)OC)c1cc(c(c(c1)OC)OC)OC	-8.1
18721	o1c(cc(=O)c2c(cc(cc12)OC(=O)C)OC(=O)C)c1ccc(cc1)OC(=O)C	-8.1
20452436	O1c2ccccc2CC[C@H]1c1ccc(cc1)O	-8.1

21270160	o1cc(c(=O)c2ccc(c(C)c12)O)c1ccc(C)cc1	-8.1
21291576	o1cc(c(=O)c2c(cc(C)cc12)O)c1ccc(cc1)O	-8.1
23644623	o1c2cc(ccc2c(=O)c(c1n1cncc1)c1ccccc1)OC	-8.1
23786424	O1c2c(c(cc(c2C(=O)C[C@@H]1c1ccccc1)OC)O)OC	-8.1
245874	O1c2cc(c(c(c2C(=O)C[C@H]1c1ccc(cc1)OC)OC)O)OC	-8.1
25058047	o1c(c(c(=O)c2c(cc(c(c12)CC=C(C)C)OCCO)O)OCCO)c1ccc(cc1)OC	-8.1
25201019	O1c2cc(cc(O)c2C(=O)C[C@H]1c1ccc(cc1)OC)O	-8.1
3083783	o1c(cc(=O)c2c(c(c(c(c12)O)OC)OC)O)c1ccc(cc1)OC	-8.1
4183640	c1(ccc2c(=O)c(coc2c1)c1ccc(cc1)O)O[C@H]1O[C@@H]([C@H]([C@H]([C@@H]1O)O)O)CO	-8.1
44260080	o1c(c(c(=O)c2c(c3c(c(c12)OC)OCO3)OC)OC)c1cc(c2c(OCO2)c1)OC	-8.1
44566483	O1c2cc(c(c(c2C(=O)C[C@H]1c1ccc(cc1)OC)OC)O)OC	-8.1
5281949	o1c(c(c(=O)c2c(cc(c(c12)C(C)(C)C=C)O)O)O)c1ccc(cc1)OC	-8.1
5310669	c1(coc2cc(ccc2c1=O)O)c1ccc(F)cc1	-8.1
5317652	O1c2ccc(c(c2C=CC1(C)C)O)c1coc2cc(ccc2c1=O)O	-8.1
5321859	o1c(cc(=O)c2c(c(c(c(c12)OC)O)OC)O)c1ccc(c(c1)O)O	-8.1
5379265	o1c2cc(cc(c2c(=O)cc1c1cc(c(c(c1)OC)OC)OC)O)O	-8.1
5386963	o1c(c(c(=O)c2c(c(c(c(c12)OC)O)OC)O)OC)c1cc(c(c(c1)OC)OC)OC	-8.1
54140747	c1(c(=O)c2ccccc2oc1c1ccccc1)OS(=O)(=O)O	-8.1
54450648	O1c2ccccc2C(=O)/C(=C\Nc2ccccc2O)/[C@H]1c1ccccc1	-8.1
5488781	o1cc(c(=O)c2c(c(c(cc12)O)OC)O)c1ccc(c(c1)O)OC	-8.1
5490001	o1c2cc(ccc2c(=O)c(c1c1cc(c(c(c1)OC)OC)OC)OC)O	-8.1
5491643	o1c(cc(=O)c2c(c(c(cc12)O)OC)OC)c1ccc(c(c1)OC)OC	-8.1
59331779	o1c(cc(=O)c2c(cc(cc12)OCC#N)OC)c1ccc(c(c1)OC)OC	-8.1
631170	o1c(cc(=O)c2c(cc(cc12)OC)OC)c1ccc(c(c1)OC)OC	-8.1
688827	o1c2ccccc2c(=O)c(c1c1cc(c(c(c1)OC)OC)OC)O	-8.1

736738	O1c2ccccc2C(=O)[C@@H]([C@H]1c1ccccc1)O	-8.1
79730	o1c(cc(=O)c2c(cc(cc12)OC)OC)c1ccc(cc1)OC	-8.1
847733	O1c2ccccc2C[C@@H]([C@H]1c1ccccc1)O	-8.1
9064	O1c2cc(cc(c2C[C@@H]([C@H]1c1ccc(c(c1)O)O)O)O)O	-8.1
97860	O1c2ccc(cc2C(=O)C[C@H]1c1ccccc1)OC	-8.1
10050660	O1c2c(c(cc(c2[C@@H]2C(=O)c3ccc(cc3O2)OC)OC)OC)C(=O)C[C@H]1c1ccc(cc1)OC	-8.0
100943379	c1(ccc(cc1)c1cc(=O)c2ccccc2o1)S(=O)(=O)N(CC)CC	-8.0
101028405	o1c(cc(=O)c2c(cc(c(c12)NC(C)C)OC)OC)c1ccccc1	-8.0
101591281	c1(cc(c2c(=O)c(c(oc2c1OC)c1ccc(c(c1)OC)O)OC)OC)OCCCCC	-8.0
102320040	o1cc(c(=O)c2c(c(c(c(c12)O)O)OC)O)c1ccc(cc1)O	-8.0
102330217	O1c2cc(ccc2C(=O)[C@@H]([C@@H]1c1ccc(cc1)OC)O)OC	-8.0
10742453	O1[C@@H](CC(=O)c2c(cc(c(c12)C[C@@H](C(=C)C)CCC(C)(C)O)O)O)c1ccc(cc1OC)O	-8.0
10755200	o1c2ccc(cc2c(=O)cc1c1ccc(cc1)N(C)C)N	-8.0
12093281	O1c2ccccc2C(=O)/C(=C\c2ccc(cc2)N(=O)=O)/[C@H]1c1ccccc1	-8.0
12132870	o1cc(c(=O)c2cc(c(cc12)OC)O)c1ccc(cc1)OC	-8.0
12405140	O1c2cc(c(c(c2C(=O)C[C@@H]1c1ccc(c(c1)OC)OC)OC)OC)OC	-8.0
129661517	O1c2cc(cc(c2[C@H](C(=O)[C@@H]1c1ccccc1)O)O)O	-8.0
129661527	O1c2cc(cc(c2[C@H](C(=O)[C@@]1(c1ccccc1)O)O)O)O	-8.0
129680256	O1c2ccccc2CC([C@H]1c1ccccc1)(O)O	-8.0
12971122	O1C2=C(C=CCC2=O)CC[C@@H]1c1ccccc1	-8.0

0		
129733622	O1c2ccc(c(c2C(=O)C([C@@]1(c1ccccc1)O)(O)O)O)OC	-8.0
129852160	c1(cccc2oc(c(c(=O)c12)O)c1ccccc1)[C@@H]1O[C@@H]([C@@H]([C@@H]([C@H]1O)O)O)CO	-8.0
129856151	O(c1c(=O)c2ccccc2oc1c1ccccc1)C(=O)[C@H]1O[C@@H]([C@@H]([C@@H]([C@@H]1O)O)O)O	-8.0
14057036	O1c2cc(c3c(=O)c(coc3c2C=CC1(C)C)c1cc(c(cc1OC)OC)OC)O	-8.0
147806	O1c2ccccc2C(=O)[C@H]([C@@H]1c1ccccc1)O	-8.0
167616	O1c2cc(cc(c2C(=O)C[C@H]1c1ccc(c(c1)OC)OC)OC)OC	-8.0
21270125	c1(coc2cc(c(cc2c1=O)O)O)c1ccc(Cl)cc1	-8.0
21626042	o1cc(c(=O)c2c(c3c(cc12)OCO3)OC)c1cc2c(cc1OC)OCO2	-8.0
23724666	c1(coc2cc(ccc2c1=O)O)c1c(cc2OCOc2c1)O	-8.0
25068463	O1c2cc(cc(c2CC(=O)[C@H]1c1ccc(c(c1)OC)OC)OC)OC	-8.0
261859	o1c(cc(=O)c2c(c(c(c(c12)OC)OC)OC)O)c1cc(c(c(c1)OC)OC)OC	-8.0
3070246	c1(c(C)c(=O)c2ccc(c(c2o1)CN(CC)CC)OC)c1ccc(Cl)cc1	-8.0
3245800	c1(cc(=O)c2ccccc2o1)c1ccc(cc1)SC	-8.0
35028119	o1c(cc(=O)c2c(c(c(c(c12)OC)OC)OC)OC)c1cccc(c1)OC	-8.0
3747	c1(ccc2c(=O)c(coc2c1)c1ccccc1)OC(C)C	-8.0
44257042	O1[C@H](C=C[C@H]2C(=C([C@H](C=C12)O)O)OC)c1ccc(c(c1)O)O	-8.0
44258304	o1c(cc(=O)c2c(cc(cc12)OC)OC)c1cc(c(cc1O)O)O	-8.0
442583	o1c(cc(=O)c2c(c(c(cc12)OC)OC)OC)c1ccccc1	-8.0
44259864	o1c(c(c(=O)c2c(c(c3c(cco3)c12)OC)OC)OC)c1ccc2c(OCO2)c1	-8.0
5003667	O1c2cc(cc(c2C(=O)C[C@H]1c1ccc(cc1)OC)OC)OC	-8.0
5353911	o1cc(c(=O)c2c(cc(c(c12)OC)O)O)c1ccc(cc1)O	-8.0
5372237	o1c2cccc(c2c(=O)c(c1c1ccc(cc1)OC)C(=O)c1ccc(cc1)OC)O	-8.0

5380905	o1c2cc(cc(c2c(=O)c(c1c1ccc(c(c1)O)OC)OC)O)O	-8.0
5381920	o1c2cc(ccc2c(=O)cc1c1cc(c(c(c1)OC)OC)OC)O	-8.0
54490901	O1c2ccccc2C(=O)/C(=C\c2cccc(c2)NC(=O)C)/[C@H]1c1ccccc1	-8.0
5491929	o1cc(c(=O)c2c(c3c(cc12)OCO3)O)c1ccccc1OC	-8.0
586387	O1c2cc(cc(c2C(=O)[C@@H]([C@@H]1c1ccc(cc1)OC)O)O)O	-8.0
629965	o1c(cc(=O)c2c(c(ccc12)OC)OC)c1c(cccc1OC)OC	-8.0
631095	o1c(c(c(=O)c2c(cc(cc12)OC)OC)OC)c1ccc(cc1)OC	-8.0
633124	o1c(cc(=O)c2c(c(c(c(c12)OC)O)OC)O)c1cc(c(c(c1)OC)OC)O	-8.0
6710704	o1cc(c(=O)c2c(cc(cc12)OC)OC)c1ccccc1	-8.0
67743168	O1c2cc(cc(c2[C@H]([C@@H]([C@@H]1c1cccc(c1)O)O)O)O)O	-8.0
740761	c1(coc2cc(ccc2c1=O)OC(C)C)c1c(F)cccc1	-8.0
86641846	o1c(cc(=O)c2c(cc(cc12)OCC#C)OC)c1ccc(c(c1)OC)OC	-8.0
9813808	O1c2ccccc2C[C@H]([C@H]1c1ccc(c(c1)O)O)O	-8.0
10121375<br>9	o1cc(c(=O)c2c(cc(cc12)O)O)c1c(cc(c(c1)CC=C(C)C)OC)O	-7.9
10121493<br>7	o1c(c(c(=O)c2cc(ccc12)CN(CC)CC)O)c1ccc(cc1)N(CC)CC	-7.9
10139688<br>7	O1c2c3c(=O)c(coc3c(c(c2C[C@@H]1C(C)(C)O)O)CC=C(C)C)c1ccc(cc1)O	-7.9
10159128<br>2	c1(cc(c2c(=O)c(c(oc2c1OC)c1ccc(c(c1)OC)OC)OC)OC)OCCCCC	-7.9
10188771<br>8	c1(c(cc(c2c(=O)cc(oc12)c1ccc(cc1)O)O)O)[C@@H]1O[C@@H](C)[C@@H]([C@@H](C1)O)O	-7.9
10386481	o1c(cc(=O)c2c(c(c(c(c12)OC)OC)OC)O)c1cc(c(c(c1)OC)O)OC	-7.9
10468234	O1c2ccccc2C(=O)[C@@](C)([C@H]1c1ccc(C)cc1)O	-7.9
10594862	Brc1c(=O)c2ccccc2oc1c1ccc(cc1)[S@@](=O)C	-7.9
10759582	O1c2cc(c(c(c2C(=O)C[C@H]1c1ccc(cc1)O)O)C(C)(C)C=C)O	-7.9

12043067	c1(cc(=O)c2c(cc(c(c2o1)[C@@H]1C(=O)CN(C)CC1)O)O)c1c(Cl)cccc1	-7.9
124037363	O1c2ccccc2[C@@H]([C@@H]([C@H]1c1ccccc1)O)O	-7.9
129675099	o1c2ccccc2c(=O)c(c1c1ccccc1)OC(=O)c1cc(c(c(c1)O)O)O	-7.9
13916267	o1c(c(c(=O)c2c(cc(c(c12)OC)O)OC)OC)c1ccc(c(c1)OC)OC	-7.9
14372618	o1c(cc(=O)c2c(c(c(cc12)OC)O)OC)c1ccccc1	-7.9
14630497	o1c(c(c(=O)c2c(cc3c(C=CC(C)(C)O3)c12)O)CCC(C)(C)O)c1cc(c(cc1O)O)O	-7.9
15126657	o1cc(c(=O)c2ccc(c(C)c12)O)c1ccc(cc1)O	-7.9
15625549	O1c2c(c(c(c(c2C(=O)C[C@H]1c1ccc(c(c1)OC)OC)O)OC)OC)OC	-7.9
181092	o1c(cc(=O)c2c(c(c(c(c12)OC)OC)OC)O)c1ccc(c(c1)OC)O	-7.9
18324915	c1(c(c(=O)c2ccccc2o1)c1cccc(F)c1)c1ccc(cc1)S(=O)(=O)C	-7.9
25022469	o1c(cc(=O)c2c(c(cc(c12)OC)OC)OC)c1cc(ccc1OC)OC	-7.9
25585	o1c(C)c(c(=O)c2cccc(c12)OCCN(C)C)c1ccc(cc1)OC	-7.9
282014	O1c2cc(cc(c2C[C@H]([C@H]1c1ccc(cc1)O)O)O)O	-7.9
3080750	o1c(cc(=O)c2c(c(c(c(c12)OC)OC)OC)O)c1ccc(c(c1)O)OC	-7.9
3083840	o1cc(c(=O)c2cc(c(cc12)OC(=O)C)OC)c1ccc(cc1)OC	-7.9
3084507	o1c(cc(=O)c2c(c(c(c(c12)OC)OC)OC)O)c1cc(c(c(c1)OC)OC)O	-7.9
3084508	o1c(cc(=O)c2c(c(c(c(c12)OC)OC)OC)O)c1cc(c(c(c1)O)OC)O	-7.9
331149	o1c(cc(=O)c2c(c(c(c(c12)OC)OC)OC)OC)c1cc(c2c(OCO2)c1)OC	-7.9
369612	O1c2c(cc(cc2[C@H](C[C@H]1c1ccc(c(c1)OC)OC)OC)CC=C)OC	-7.9
462696	O1c2cc(cc(c2C(=O)C[C@H]1c1cc(c(c(c1)OC)OC)OC)O)O	-7.9
493376	o1c2cc(cc(c2c(=O)cc1c1cc(c(c(c1)OC)OC)OC)OC)OC	-7.9
49788614	o1c2cc(cc(c2c(=O)c(c1c1ccc2c(OCO2)c1)OCC=C(C)C)O)OC	-7.9
5280373	o1cc(c(=O)c2c(cc(cc12)O)O)c1ccc(cc1)OC	-7.9
5281695	o1c(c(c(=O)c2c(c(c(cc12)O)OC)O)OC)c1ccc(cc1)OC	-7.9

5281804	o1cc(c(=O)c2c(cc(cc12)OC)O)c1ccc(cc1)O	-7.9
5281811	o1cc(c(=O)c2c(c(c(cc12)O)OC)O)c1ccc(cc1)O	-7.9
5284649	o1cc(c(=O)c2cc(c(cc12)O)O)c1ccc(cc1)O	-7.9
53233892	c1(ccc2c(c(=O)cc(o2)c2ccccc2)c1)OP(=O)(OCC)OCC	-7.9
5385091	c1(coc2cc(ccc2c1=O)O)c1c(cc2OCOc2c1)OC	-7.9
54070148	c1(ccc(cc1)OCCO)[C@H]1Oc2ccccc2CC1	-7.9
5481647	o1c(cc(=O)c2c(c(c(cc12)O)OC)OC)c1ccccc1	-7.9
57409964	c1(cc(c(c(c1)C[C@@H](C(=C)C)O)O)C/C=C(/C)\CO)c1oc2cc(c(c(c2c(=O)c1OC)O)OC)O	-7.9
5748605	o1cc(c(=O)c2ccc(cc12)O)c1ccc(c(c1)OC)OC	-7.9
614295	[C@H]1(C(=O)c2ccccc2O[C@@H]1c1ccccc1)SCCC	-7.9
68077	o1c(cc(=O)c2c(c(c(c(c12)OC)OC)OC)OC)c1ccc(cc1)OC	-7.9
69047096	c1(ccc(cc1OCOC)OCOC)[C@@H]1Oc2ccccc2C(=O)C1	-7.9
72344	o1c(cc(=O)c2c(c(c(c(c12)OC)OC)OC)OC)c1ccc(c(c1)OC)OC	-7.9
7309334	O1c2ccccc2C[C@H]([C@@H]1c1ccccc1)O	-7.9
7454026	o1cc(c(=O)c2ccc(cc12)O)c1ccc(cc1)N(=O)=O	-7.9
91584129	o1c2cc(c(c(c2c(=O)cc1c1ccc(c(c1OC)OC)O)O)OC)O	-7.9
101028409	o1c(cc(=O)c2c(cc(c(c12)NCC=C)OC)OC)c1ccccc1	-7.8
101561898	o1c2ccccc2c(=O)c(c1c1ccccc1)OCC#C	-7.8
10814211	o1c(c(c(=O)c2c(c(c(c(c12)OC)OCC=C(C)C)OC)OC)OC)c1ccc2c(OCO2)c1	-7.8
11048450	O1C(C)(C)C=Cc2c(c3C(=O)C[C@H](Oc3c(c12)C[C@@H](C(C)(C)O)O)c1ccc(cc1)O)O	-7.8
12407921	o1c2ccc(cc2c(=O)cc1c1ccc(cc1)OCCO)C(=O)O	-7.8
129674781	O1c2ccccc2C[C@@H]([C@@H]1c1ccccc1)OC	-7.8
12983450	O1c2cc(cc(c2C(=O)C[C@H]1c1ccc(cc1)OC(=O)C)OC)OC(=O)C	-7.8

7		
129844543	o1cc(c(=O)c2c(c(c(c(c12)O)O)OC)O)c1cc(c(c(c1)OC)OC)O	-7.8
13873818	o1c(cc(=O)c2c(c(c(cc12)OCc1ccccc1)OC)OC)c1ccc(c(c1)OC)OC	-7.8
14304977	o1c2ccccc2c(=O)c(c1c1cc(c(c(c1)OC)OC)OC)C(=O)O	-7.8
182659	O1c2cc(cc(c2C[C@@H]([C@H]1c1ccc(c(c1)OC)OC)O)OC)OC	-7.8
18324902	c1(c(c(=O)c2ccccc2o1)c1ccc(cc1)F)c1ccc(cc1)S(=O)(=O)C	-7.8
358832	o1c(cc(=O)c2c(c(c(c(c12)OC)OC)OC)O)c1ccc(c(c1)OC)OC	-7.8
369615	O1c2c(cccc2C(=C)C[C@@H]1c1ccc(c(c1)OC)O)OC	-7.8
42607933	O1c2c(c(cc(c2C[C@@H](C(=C)C)O)O)O)C(=O)C[C@H]1c1ccc(cc1)O	-7.8
42607957	O1c2c(c(cc(c2C(=O)C[C@H]1c1ccc(cc1)OC)O)O)CC(=C(C)C)O	-7.8
42608126	O1c2c(c(c(c(c2C(=O)C[C@H]1c1cc(c2c(OCO2)c1)OC)OC)OC)OC)OC	-7.8
44257043	O1[C@H](C=C[C@H]2C(=C([C@H](C=C12)O)O)OC)c1ccc(c(c1)O)OC	-7.8
44260053	o1c(c(c(=O)c2c(c(c(c(c12)OC(=O)CC(C)C)O)OC)O)OC)c1ccc(cc1)OC	-7.8
5280961	o1cc(c(=O)c2c(cc(cc12)O)O)c1ccc(cc1)O	-7.8
5281676	o1c2cc(cc(c2c(=O)c(c1c1cc(c(cc1O)OC)O)OC)O)OC	-7.8
5281781	o1cc(c(=O)c2c(c(c(cc12)O)OC)O)c1ccc(cc1)OC	-7.8
5281801	o1cc(c(=O)c2c(cc(cc12)O)O)c1ccc(c(c1)O)O	-7.8
5284648	o1cc(c(=O)c2ccc(cc12)O)c1ccc(c(c1)O)O	-7.8
5376891	o1cc(c(=O)c2ccc(cc12)O)c1ccccc1	-7.8
5378391	o1c(C)c(c(=O)c2ccc(c(c12)CC=C)O)c1ccccc1	-7.8
5385026	o1c(c(c(=O)c2c(c(c(cc12)O)OC)OC)O)c1ccccc1	-7.8
5417907	o1c2cc(ccc2c(=O)c(c1C)c1ccc(cc1)N(=O)=O)O	-7.8
5487785	o1cc(c(=O)c2c(c(c(cc12)OC)OC)O)c1ccc(cc1)O	-7.8
5491412	o1c2cc(cc(c2c(=O)c(c1c1cc(c(c(c1)O)OC)O)OC)O)OC	-7.8
628412	o1c(c(c(=O)c2c(cc(cc12)OC)OC)OC)c1ccccc1OC	-7.8

628555	o1c2cc(ccc2c(=O)c(c1c1ccc(cc1)OC)OC)OC	-7.8
638006	o1cc(c(=O)c2ccc(cc12)OC)c1ccccc1	-7.8
96539	o1c(cc(=O)c2c(c(c(c(c12)OC)OC)OC)O)c1ccc(cc1)OC	-7.8
101028406	o1c(cc(=O)c2c(cc(c(c12)NCCC)OC)OC)c1ccccc1	-7.7
101028407	o1c(cc(=O)c2c(cc(c(c12)NCC(C)C)OC)OC)c1ccccc1	-7.7
101179165	o1c(c(c(=O)c2c(cc(cc12)OC)OCCCO)OC)c1ccc(c(c1)OC)OC	-7.7
101650843	O1c2ccccc2C(=O)/C(=C\c2cccc(c2)N(=O)=O)/[C@H]1c1ccccc1	-7.7
10315196	O1c2cc(c(c(c2C(=O)C[C@@H]1c1ccc(cc1)O)O)C(C)(C)C=C)O	-7.7
10665307	Brc1c(=O)c2ccccc2oc1c1ccc(cc1)SC	-7.7
129733750	Cl[C@H]1c2ccccc2O[C@@H]([C@@H]1O)c1ccccc1	-7.7
129882752	o1cc(c(=O)c2c(cc(c(c12)OC)OC)OC)c1ccc(cc1)O	-7.7
13045742	O1c2cc(cc(c2CC[C@H]1c1ccc(c(c1)OC)OC)OC)OC	-7.7
13045744	O1c2cc(cc(c2CC[C@H]1c1ccc(c(c1)OC)OC)OC)OC	-7.7
132507960	o1cc(c(=O)c2cc(c(cc12)CO)OC)c1ccc(cc1)O	-7.7
14732294	O1c2cc(cc(c2C[C@@H]([C@@H]1c1ccc(c(c1)O)O)O)OC)OC	-7.7
17585058	c1(ccc2c(=O)c(coc2c1)c1ccccc1)OCCBr	-7.7
18324974	c1(c(=O)c2ccccc2oc1c1ccc(cc1)S(=O)(=O)C)c1ccc(Cl)cc1	-7.7
185027	O1c2c(c(ccc2C(=O)C[C@@H]1c1ccccc1)OC)/C=C/C(=C)C	-7.7
189551	O1C(=C(C(=O)[C@]2(CC=CC(=C12)OC)O)OC)c1ccccc1	-7.7
20452422	O1c2ccccc2CC[C@H]1c1ccc(cc1)N(C)C	-7.7

21573556	O1c2cc(cc(c2C(=O)C[C@H]1c1ccc(c(c1OC)OC)OC)OC)OC	-7.7
25232840	O1c2cc(cc(c2CC[C@H]1c1ccc(cc1)OC)O)O	-7.7
343083	c1(coc2cc(ccc2c1=O)OC)c1c(cc2OCOc2c1)OC	-7.7
44133602	o1cc(c(=O)c2c(c(c(cc12)O)OC)O)c1cc(c(c(c1)OC)O)OC	-7.7
462698	O1c2c(c(c(c(c2C(=O)C[C@@H]1c1ccc(cc1)OC)OC)OC)OC)OC	-7.7
5280448	o1cc(c(=O)c2ccc(cc12)O)c1ccc(c(c1)O)OC	-7.7
5318041	o1c(cc(=O)c2c(c(c(c(c12)OC)OC)OC)OC)c1ccc(c(c1)O)O	-7.7
5318979	O1c2ccccc2[C@@H]([C@@H]([C@@H]1c1ccccc1)O)O	-7.7
5319422	o1cc(c(=O)c2ccc(cc12)O)c1ccc(c(c1)OC)O	-7.7
5481229	O1c2c(c(cc(c2C=CC1(C)C)c1coc2cc(cc(c2c1=O)O)O)OC)O	-7.7
56648912	o1c(cc(=O)c2c(c(c(cc12)OCC=C)OC(=O)C)OC(=O)C)c1ccccc1	-7.7
71346541	o1c2ccccc2c(=O)c(c1c1ccc(cc1)N(C)C)OC	-7.7
71428798	o1cc(c(=O)c2ccc(c(c12)OC)O)c1ccc(cc1)O	-7.7
74977227	O1c2cc(cc(c2CC[C@H]1c1ccc(c(c1)OC)OC)O)O	-7.7
911486	o1c2cc(ccc2c(=O)c(c1C)c1ccc(cc1)N(=O)=O)OC	-7.7
10069029	c1(c(c(=O)c2ccc(cc2o1)O)c1ccc(cc1)OC)SCc1ccncc1	-7.6
10317448	c1(c(c(=O)c2ccc(cc2o1)O)c1ccc(cc1)O)SCc1ccncc1	-7.6
10526049	o1c(c(c(=O)c2c(c(c(c(c12)OC)OC)OCOC)OC)O)c1ccc(cc1)OC	-7.6
11417114	c1(c(c(=O)c2ccc(cc2o1)O)c1ccccc1)SCc1cnccc1	-7.6
11704980	o1c2ccccc2c(=O)c(c1c1cc(c(c(c1)OC)OC)OC)C(=O)c1ccc(cc1)OC	-7.6
129035976	O1c2ccccc2C[C@@H]([C@@H]1c1ccccc1)O	-7.6
129662374	o1c2ccccc2c(=O)c(c1C)c1ccc(cc1)N(=O)=O	-7.6
129674619	[C@H]1(c2ccccc2O[C@H](C1)c1ccccc1)S	-7.6

129734164	o1c(c(c(=O)c2c(c(c(c(c12)O)OC)OC)OC)O)c1ccccc1O	-7.6
129864121	o1c2cc(cc(c2c(=O)c(c1c1ccc(cc1)O[C@H](C(=C)C)CO)OC)O)OC	-7.6
132517368	o1c(cc(=O)c2c(c(C)c(c(c12)C(C)(C)O)OC)O)c1ccc(cc1)OC	-7.6
136418	o1c(cc(=O)c2c(c(c(cc12)OC(=O)C)OC(=O)C)OC(=O)C)c1ccc(cc1)OC(=O)C	-7.6
139030757	o1cc(c(=O)c2ccc(c(c12)OC)CCCO)c1ccc(cc1)OC	-7.6
14304994	c1(c(=O)c2ccccc2oc1c1ccccc1)C(=O)N1CCN(CC1)Cc1ccccc1	-7.6
14630603	o1cc(c(=O)c2c(c3c(cc12)OCO3)OC)c1cccc(c1OC)OC	-7.6
14804713	o1c(c(c(=O)c2c(C)cc(cc12)OC)OC)c1ccc(cc1)O	-7.6
14886014	o1c2c(c(cc(c2c(=O)c(c1c1ccc2c(OCO2)c1)OC)OC)O)OC	-7.6
18325002	c1(c(c(=O)c2ccccc2o1)c1ccccc1F)c1ccc(cc1)S(=O)(=O)C	-7.6
22134047	o1c(c(c(=O)c2c(c(ccc12)OC)OC)O)c1ccccc1	-7.6
44257187	O1c2c(c(cc(c2CC[C@H]1c1ccccc1)OC)OC)CCC(C)(C)O	-7.6
44412009	O1c2c(C)c(cc(c2C(=O)C[C@H]1c1ccccc1)OC)OC	-7.6
5281704	o1cc(c(=O)c2cc(c(cc12)O)OC)c1ccc(cc1)OC	-7.6
631791	o1c(c(c(=O)c2c(c(c3c(cco3)c12)OC)OC)OC)c1ccccc1	-7.6
632135	o1c(cc(=O)c2c(cc(c(c12)OC)OC)OC)c1ccc(c(c1)OC)OC	-7.6
66692593	O1c2ccccc2C[C@@H]([C@@H]1c1ccccc1)O	-7.6
9821032	c1(c(c(=O)c2ccccc2o1)c1ccccc1)c1ccc(cc1)S(=O)(=O)C	-7.6
9928523	O1c2c(c(cc(c2C(=O)C[C@H]1c1ccc(cc1)O)OC)O)CC=C(C)C	-7.6
101028408	o1c(cc(=O)c2c(cc(c(c12)NCCC(C)C)OC)OC)c1ccccc1	-7.5
11244229	c1(c(c(=O)c2ccc(cc2o1)OC)c1ccccc1)SCC=C	-7.5
11795367	o1c(c(c(=O)c2c(c(c(c(c12)OC)O)OC)OC)OC)c1ccc2c(OCO2)c1	-7.5

12428921	O1c2ccccc2C(=O)/C(=C\c2cccc(c2)O)/[C@H]1c1ccccc1	-7.5
13916278	o1c(c(c(=O)c2c(cc(c(c12)OC)O)O)OC)c1ccc(cc1)OC	-7.5
15761525	O1c2ccccc2C(=O)C([C@H]1c1ccccc1)(CO)CO	-7.5
16215025	c1(cc(c(c2O[C@H](CC(=O)c12)c1ccccc1)OC)OC)O[C@@H]1O[C@@H]([C@H]([C@@H]([C@H]1O)O)O)CO	-7.5
18324995	c1(c(c(=O)c2ccccc2o1)c1ccccc1F)c1ccc(cc1)S(=O)(=O)N	-7.5
369616	O1c2c(cc(cc2[C@H](C[C@H]1c1ccc(c(c1)OC)OC)OC(=O)C)CC=C)OC	-7.5
386331	o1c(c(c(=O)c2c(c(c(cc12)OC)OC)OC)OC)c1ccc(c(c1)OC)OC	-7.5
44257206	o1c(C)c(c(=O)c2ccc(c(c12)C(=O)C)O)c1ccccc1	-7.5
5282074	o1cc(c(=O)c2c(cc(cc12)O)O)c1ccc(cc1O)O	-7.5
5322009	o1c(cc(=O)c2c(c(c(cc12)OC(=O)C)OC)OC(=O)C)c1ccc(cc1)OC(=O)C	-7.5
54423602	c1(c(c(=O)c2ccccc2o1)c1ccccc1)OC(C)C	-7.5
626146	o1c(C)c(c(=O)c2ccc(cc12)OCC=C)c1ccccc1	-7.5
631176	o1cc(c(=O)c2cc(c(cc12)OC)OC)c1ccc(c(c1)OC)OC	-7.5
634440	o1c(c(c(=O)c2c(c(c(cc12)OC)OCC)OCC)OC)c1ccc(c(c1)OCC)OC	-7.5
71586772	o1c(cc(=O)c2c(c(c(cc12)OC(=O)C)OC)OC(=O)C)c1ccccc1	-7.5
97151	o1c(cc(=O)c2c(c(c(c(c12)OC)OC)OC)OC)c1ccc2c(OCO2)c1	-7.5
101665962	o1c2cc(ccc2c(=O)c(c1c1ccc(c(c1)OCCO)OCCO)OCCO)OCCO	-7.4
102194909	o1c(c(c(=O)c2c(c(c(c(c12)OC)OCC=C(C)C)OC)OC)OC)c1ccc(c(c1)OC)OC	-7.4
11610052	o1cc(c(=O)c2c(cc(cc12)O)O)c1ccc(c(c1CC=C(C)C)O)O	-7.4
12043064	c1(cc(=O)c2c(cc(c(c2o1)[C@@H]1CN(C)CC[C@H]1O)O)O)c1c(Cl)cccc1	-7.4
129820554	o1c(c(c(=O)c2c(cc(c(c12)OC)OC)OC)O)c1ccc(c(c1)OC)OC	-7.4
129837195	o1c(c(c(=O)c2c(c(c(c(c12)O)O)O)OC)OC)c1ccccc1O	-7.4

129881940	c1(c(=O)c2ccccc2oc1c1ccccc1)[C@@H]1O[C@H](C)[C@@H]([C@H](C1)O)O	-7.4
13045799	O1c2ccccc2C[C@@H]([C@@H]1c1ccccc1)OC(=O)C	-7.4
132517366	o1c(cc(=O)c2cc(c(c(c12)/C=C(\C)/CO)OC)O)c1ccc(cc1)OC	-7.4
136420	o1cc(c(=O)c2c(cc(cc12)OC)OC)c1ccc(cc1)OC	-7.4
139030767	o1cc(c(=O)c2ccc(c(c12)OC)C(=O)CCO)c1ccc(cc1)OC	-7.4
18324937	c1(c(c(=O)c2ccccc2o1)c1ccc(cc1)F)c1ccc(cc1)S(=O)(=O)N	-7.4
44257040	O1[C@H](C=C[C@H]2C(=C([C@H](C=C12)O)O)OC)c1ccc(cc1)OC	-7.4
5281706	o1cc(c(=O)c2c(cc(cc12)OC)O)c1ccc(cc1O)O	-7.4
53796934	o1cc(c(=O)c2c(cc(cc12)OC)O)c1ccc(cc1O)OC	-7.4
5466794	c1(cc(=O)c2c(cc(c(c2o1)[C@@H]1CCN(C)C[C@H]1O)O)O)c1c(Cl)cccc1	-7.4
6116651	O1c2ccccc2C(=O)/C(=C\c2ccncc2)/[C@H]1c1ccccc1	-7.4
633456	o1c2cc(ccc2c(=O)c(c1C)c1cc(c(cc1OC)OC)OC)OC(=O)C	-7.4
9864504	c1(c(c(=O)c2ccccc2o1)c1ccccc1)c1ccc(cc1)S(=O)(=O)N	-7.4
102321610	c1(coc2cc(cc(c2c1=O)O)O)c1ccc2OCOc2c1OC	-7.3
10957930	O1c2ccccc2C(=O)/C(=C/c2ccccc2)/[C@@H]1c1ccccc1	-7.3
11024735	O1c2ccccc2C(=O)/C(=C/c2ccc(cc2)N(=O)=O)/[C@@H]1c1ccccc1	-7.3
11176755	c1(c(c(=O)c2ccc(cc2o1)OC)c1ccc(C)cc1)SCc1ccncc1	-7.3
11500888	O1c2cc(cc(c2C(=O)[C@@H]([C@H]1c1ccc(cc1OC)O)O)OC)O	-7.3
12043063	c1(cc(=O)c2c(cc(c(c2o1)[C@@H]1CN(C)CC[C@H]1O)OC)OC)c1c(Cl)cccc1	-7.3
12310757	c1(c(cc(c2c(=O)c(c(oc12)c1ccc(cc1)OC)O)O)O)CCC(C)(C)O	-7.3
129762930	O1c2cc(cc(c2C(=O)C[C@]1(c1ccc(cc1)O)OC)O)OC	-7.3
12983478	o1c(c(c(=O)c2c(c(c(cc12)OC)OC)OC)OCC)c1ccc(c(c1)OCC)OC	-7.3

8		
129866052	o1c2ccccc2c(=O)c(c1c1ccccc1)[C@H]1CNCCN1Cc1ccccc1	-7.3
15761527	C1(C(=O)c2ccccc2O[C@H]1c1ccccc1)(CCl)CCl	-7.3
23644938	c1(c(c(=O)c2ccc(cc2o1)OC)c1ccccc1)Sc1ncn[nH]1	-7.3
44257041	O1[C@H](C=C[C@H]2C(=C([C@H](C=C12)O)O)OC)C1=CCC(=CC1)O	-7.3
51041990	o1c2cc(cc(c2c(=O)c(c1c1cc(cc(c1CC=C(C)C)O)OC)OC)O)O	-7.3
54140898	o1c(c(c(=O)c2c(c(ccc12)OCC)OCC)OCC)c1ccccc1	-7.3
54728923	o1c(=O)c(c(c2c(cc(cc12)O)O)O)c1ccc(cc1)O	-7.3
631782	o1c2cc(ccc2c(=O)c(c1C)c1ccc(cc1OC)OC)OCC=C	-7.3
634724	o1c(c(c(=O)c2c(c(c(cc12)OCC)OC)OCC)OC)c1ccc(c(c1)OC)OCC	-7.3
91727421	O1c2cc(cc(c2C(=O)[C@H]([C@@H]1c1ccc(c(c1)OC)OC)OC(=O)C)OC)OC	-7.3
101887721	c1(c(cc(c2c(=O)cc(oc12)c1ccc(cc1)O)O)OC)[C@@H]1O[C@@H](C)[C@@H](C(=O)C1)O	-7.2
10551087	O1c2c3c(cc(c2C[C@H](C1(C)C)O)[C@@H]1CC(=O)c2c(cc(cc2O1)O)O)CCC(C)(C)O3	-7.2
11471620	o1cc(c(=O)c2ccc(cc12)OC)c1ccc(cc1O)O	-7.2
11485667	c1(c(c(=O)c2ccc(cc2o1)OC)c1ccccc1)SCc1ccccn1	-7.2
12428924	O1c2ccccc2C(=O)/C(=C\c2ccc(cc2)O)/[C@H]1c1ccccc1	-7.2
129716748	[P@]12(=O)O[C@@H]3[C@@H]4c5ccccc5O[P@]5(=O)O[C@H]6[C@H]([C@H]([C@H]([C@H]([C@H]6O1)O2)C3=O)O4)O5	-7.2
129818088	Br[C@]1(C(=O)c2ccccc2O[C@H]1c1ccccc1)N(=O)=O	-7.2
129848680	o1c(c(c(=O)c2c(c(cc(c12)OC)OC)OC)OC)c1ccc(c(c1)OC)OC	-7.2
150893	o1c(c(c(=O)c2c(c(c(c(c12)OC)OC)OC)OC)OC)c1ccc(c(c1)OC)OC	-7.2
16213663	O1/C(=C/2\C=CC(=CC2)N(C)C)/C=C(c2ccc(cc12)N(CC)CC)c1ccccc1C(=O)O	-7.2
23644937	c1(c(c(=O)c2ccc(cc2o1)OC)c1ccccc1)Sc1nccn1C	-7.2

42608003	O1c2c(c3c(C(=O)C[C@H](O3)c3ccc(c(c3)O)O)c3OC(C)(C)CCc23)CCC1(C)C	-7.2
462694	O1c2cc(ccc2C(=O)[C@H]([C@H]1c1ccc(cc1)OC)OC)O	-7.2
5378260	o1cc(c(=O)c2ccc(c(c12)O)O)c1ccc(cc1O)O	-7.2
90000191	o1c(c(c(=O)c2c(c(c(c(c12)OC)OC)OC)O)O)c1cccc(c1OC)OC	-7.2
101396888	O1c2c3c(=O)c(coc3c(c(c2C=CC1(C)C)O)C[C@H](C(C)(C)O)O)c1ccc(cc1)O	-7.1
102005373	c1(cc(=O)c2c(cc(c(c2o1)[C@@H]1CN(C)CC[C@@H]1O)OC)OC)c1c(Cl)cccc1	-7.1
11246736	c1(c(c(=O)c2ccc(cc2o1)OC)c1ccc(cc1)OC)SCc1ccncc1	-7.1
129728743	c1(c(c(=O)c2ccccc2o1)c1ccccc1)[C@@H]1O[C@@H]([C@H]([C@@H]([C@H]1O)O)O)CO	-7.1
129835695	O1c2ccccc2C(=O)[C@@]([C@]1(c1ccccc1)O)(c1c(=O)c2ccccc2oc1c1ccccc1)O	-7.1
129891972	o1cc(c(=O)c2ccc(c(c12)OC)O)c1ccc(cc1O)OC	-7.1
145945693	O1[C@H]([C@@H]([C@H]([C@H]([C@]1(c1c(=O)c2ccccc2oc1c1ccccc1)O)O)O)O)CO	-7.1
16072086	c1(cc(=O)c2c(cc(c(c2o1)C1=CCN(C)CC1)O)O)c1cccc(Cl)c1	-7.1
42607799	O1c2c(c(ccc2C(=O)C[C@H]1c1ccccc1)OCC=C(C)C)/C=C/C(C)(C)O	-7.1
44265521	c1(cc(=O)c2c(cc(c(c2o1)C1=CCN(C)CC1)O)O)c1c(Cl)cc(cc1)Cl	-7.1
5280378	o1cc(c(=O)c2ccc(cc12)O)c1ccc(cc1)OC	-7.1
5281708	o1cc(c(=O)c2ccc(cc12)O)c1ccc(cc1)O	-7.1
5380187	o1c(c(c(=O)c2c(cc(cc12)O)O)c1ccc(cc1)OC)C(=O)OCC	-7.1
631156	o1cc(c(=O)c2ccc(c(c12)OC)OC)c1ccc(cc1OC)OC	-7.1
634113	o1c2cc(cc(c2c(=O)c(c1c1cc(c(c(c1)OC)OC)OC)OC)OC)OC	-7.1
101584236	O1c2c(c(cc(c2C(=O)C[C@H]1c1ccccc1)OC)OC)/C=C\C(C)(C)O	-7.0

101887720	c1(c(cc(c2c(=O)cc(oc12)c1ccc(cc1)O)O)O)[C@@H]1O[C@@H](C)[C@H]([C@@H](C1)O)O	-7.0
11164739	c1(c(c(=O)c2ccc(cc2o1)OC)c1ccccc1)SCc1cnccc1	-7.0
11797078	o1c(c(c(=O)c2c(c(c(c(c12)OC)OC)OCOC)OC)O)c1ccc(c(c1)OC)O	-7.0
129822395	Clc1c(c(c(c2c(=O)cc(oc12)c1ccc(cc1)OC(=O)C)OC(=O)C)OC(=O)C)OC(=O)C	-7.0
129852882	[S]([C@H]1C(=O)c2c(c(c(c(c2O[C@H]1c1ccc(c(c1)OC)OC)OC)OC)OC)OC)(O)O	-7.0
23270524	O1c2cc(cc(c2C(=O)[C@H]([C@@H]1c1ccc(cc1OC)OC)OC(=O)C)OC)OC	-7.0
23644932	o1c2cc(ccc2c(=O)c(c1n1cncc1)c1ccc(cc1)OC)OC	-7.0
23644936	c1(c(c(=O)c2ccc(cc2o1)OC)c1ccccc1)Sc1ncc[nH]1	-7.0
44265534	c1(cc(=O)c2c(cc(c(c2o1)C1=CCN(C)CC1)O)O)c1c(F)cccc1	-7.0
44265561	c1(cc(=O)c2c(cc(c(c2o1)C1=CCN(C)CC1)O)O)c1ccc(Cl)cc1	-7.0
5386297	o1c(c(c(=O)c2c(c(c(cc12)O)OC)O)c1cc(c(cc1OC)OC)OC)C(=O)O	-7.0
54368735	c1(c(=O)c2ccccc2oc1c1cc(c(c(c1)OC)OC)OC)C(=O)NCCBr	-7.0
632958	o1cc(c(=O)c2cc(c(cc12)OC)OC)c1cc(c(cc1OC)OC)OC	-7.0
11153057	c1(c(c(=O)c2ccc(cc2o1)OC)c1ccccc1)SCc1ccncc1	-6.9
11164320	c1(c(c(=O)c2ccc(cc2o1)O)c1ccccc1)SCc1ccncc1	-6.9
11711336	c1(c(=O)c2ccccc2oc1c1ccccc1)O[C@@H]1O[C@@H]([C@H]([C@@H]([C@H]1O)O)OC)CO	-6.9
129674706	O1c2ccccc2C[C@@H]([C@]1(c1ccccc1)C=O)O	-6.9
129678968	[C@H]1(c2ccccc2O[C@](C1)(c1ccccc1)OC)SOC(=O)C	-6.9
129716120	c1(c(=O)c2ccccc2oc1c1ccccc1)C(=O)[C@@H]1O[C@H]([C@@H]([C@H]([C@H]1O)O)O)O	-6.9
129824418	O1c2ccccc2C[C@H]([C@]1(c1ccccc1)CC(=O)O)O	-6.9

129824498	o1c(cc(=O)c2c(cc(c(c12)c1ccc(c(c1)OC)OC)OC)OC)c1ccccc1	-6.9
129824510	o1c(cc(=O)c2c(cc(c(c12)c1cc(c(c(c1)OC)OC)OC)OC)OC)c1ccccc1	-6.9
129867277	O1C(=CC(=C2CC=CC=C12)NNC(=O)c1ccccc1)c1ccccc1	-6.9
134159420	o1c2ccccc2c(=O)c(c1c1cc(c(cc1C(=O)O)O)O)c1ccccc1	-6.9
139592630	O1c2cc(ccc2[C@@H]2C[C@H]1[C@@H]1CC=C[C@H](OCO2)C1)O	-6.9
389000	o1c(c(c(=O)c2c(c(c(c(c12)OC)OC)OC)OC)OC)c1cc(c(c(c1)OC)OC)OC	-6.9
44265522	o1c(cc(=O)c2c(cc(c(c12)C1=CCN(C)CC1)O)O)c1ccccc1	-6.9
44265523	c1(cc(=O)c2c(cc(c(c2o1)C1=CCN(C)CC1)O)O)c1c(I)cccc1	-6.9
44265528	c1(cc(=O)c2c(cc(c(c2o1)C1=CCN(C)CC1)O)O)c1c(Br)cccc1	-6.9
46173806	c1(c(=O)c2ccccc2oc1c1ccccc1)O[C@@H]1O[C@@H]([C@H]([C@@H]([C@H]1O)O)O)CO	-6.9
630804	o1cc(c(=O)c2c(cc(c(c12)CC=C(C)C)OC(=O)C)OC(=O)C)c1ccc(cc1)OC(=O)C	-6.9
9951997	c1(cc(=O)c2c(cc(c(c2o1)C1=CCN(C)CC1)O)O)c1c(Cl)cccc1	-6.9
101887719	c1(c(cc(c2c(=O)cc(oc12)c1ccc(cc1)O)O)OC)[C@@H]1O[C@@H](C)[C@@H]([C@@H](C1)O)O	-6.8
102005374	c1(cc(=O)c2c(cc(c(c2o1)[C@@H]1CCCC[C@@H]1O)OC)OC)c1c(Cl)cccc1	-6.8
102360079	O1c2cc(c3C(=O)C[C@H](Oc3c2[C@H]([C@H](C1(C)C)OC(=O)C)OC(=O)C)c1ccccc1)OC	-6.8
10980660	O1c2c(c(cc(c2C(=O)C[C@H]1c1ccccc1)O)OC)/C=C/C(=O)C	-6.8
11741814	o1c(c(c(=O)c2c(c(c(c(c12)OC)OC)OC)OC)OC)c1ccc(cc1)OC	-6.8
11847318	c1(c(c(=O)c2ccccc2o1)Cn1cncc1)c1ccc(Br)cc1	-6.8
11847319	o1c2ccccc2c(=O)c(c1c1ccc(cc1)C#N)Cn1cncc1	-6.8
11953828	c1(c(=O)c2ccccc2oc1c1ccccc1)O[C@@H]1O[C@@H]([C@H]([C@@H]([C@H]1O)O)O)CO	-6.8

129776122	O1c2cc(c(c(c2C(=O)C([C@@H]1c1ccccc1)(OC)OC)O)O)OC	-6.8
165203	o1cc(c(=O)c2ccc(cc12)OC)c1cc(c(cc1OC)OC)OC	-6.8
24850296	O1c2cc(cc(c2C(=O)[C@@]([C@]1(c1ccc(c(c1)O)O)OC)(O)CC(=O)OC)O)O	-6.8
42607940	c1(c2c(C=CC(C)(C)O2)c(c2C(=O)C[C@H](Oc12)c1ccc(cc1)O)O)[C@H]([C@H]1OC1(C)C)O	-6.8
44266353	O1c2ccccc2C(=O)/C(=C/c2ccncc2)/[C@H]1c1ccccc1	-6.8
54488609	c1(c(=O)c2ccccc2oc1c1ccccc1)O[C@@H]1O[C@@H]([C@H]([C@@H]([C@H]1O)O)O)CO	-6.8
5465888	O1c2ccccc2/C(=N/N(C(=O)C)C(=O)C)/C[C@H]1c1ccccc1	-6.8
631105	o1c2cc(cc(c2c(=O)c(c1c1ccc(cc1OC)OC)OC)OC)OC	-6.8
101584232	O1c2c(c(cc(c2C(=O)C[C@H]1c1ccccc1)OC)OC)/C=C/C(=C)C	-6.7
16126803	c1(c(=O)c2ccccc2oc1c1ccccc1)O[C@@H]1O[C@@H]([C@@H]([C@@H]([C@H]1O)O)O)CO	-6.7
22737159	O1c2ccccc2C[C@@H]([C@@H]1c1ccccc1)OC(=O)c1cc(c(c(c1)O)O)O	-6.7
23724473	c1(c(=O)c2ccccc2oc1c1ccccc1)O[C@@H]1O[C@@H]([C@@H]([C@@H]([C@H]1O)O)O)CO	-6.7
44257169	O1c2c3ccoc3c(c(c2[C@H]([C@@H]([C@@H]1c1ccccc1)OC)OC)OC)OC	-6.7
102005380	c1(cc(=O)c2c(cc(c(c2o1)C1=CCN(C)CC1)OC)OC)c1c(Br)cccc1	-6.6
102180739	o1c2ccccc2c(=O)c(c1c1ccc(c(c1OC)OC)OC)c1ccc(cc1)OC	-6.6
54505099	O1c2ccccc2C(=O)/C(=C\n2cncc2)/[C@@H]1c1ccccc1	-6.6
635396	c1(c(cc(c2c(=O)cc(oc12)c1ccc(cc1)OC)OC)OC)[C@H]1O[C@@H]([C@H]([C@H]([C@@H]1O)O)O)CO	-6.6
67200543	O1c2ccccc2C(=O)[C@@H]([C@@H]1c1ccccc1)C(=O)OC(C)(C)C	-6.6
101958862	O1c2ccccc2C(=O)C[C@]1(c1ccccc1)C(=O)O	-6.5
102005376	c1(cc(=O)c2c(cc(c(c2o1)C1=CCN(C)CC1)OC)OC)c1c(Cl)cccc1	-6.5
10200537	c1(cc(=O)c2c(cc(c(c2o1)C1=CCN(C)CC1)OC)OC)c1cccc(Cl)c1	-6.5

7		
102005378	c1(cc(=O)c2c(cc(c(c2o1)C1=CCN(C)CC1)OC)OC)c1ccc(Cl)cc1	-6.5
102005379	c1(cc(=O)c2c(cc(c(c2o1)C1=CCN(C)CC1)OC)OC)c1c(F)cccc1	-6.5
129876105	O1c2ccccc2C(=O)C[C@]1(c1cn[nH]c1)c1ccccc1	-6.5
129882615	O1[C@H]([C@H]2C(=O)[C@H]3C=CC[C@@H]1[C@H]3C2=O)c1ccccc1	-6.5
238791	c1(c(cc(cc1c1cc(cc(c1OC)OC)C(=O)C)C(=O)C)OC)OC	-6.5
66780856	O1c2cccc(c2C(=O)C([C@]1(c1ccccc1)OC)(O)O)O	-6.5
102005382	c1(cc(=O)c2c(cc(c(c2o1)C1=CCN(C)CC1)OC)OC)c1c(Cl)cc(cc1)Cl	-6.4
129710808	O1c2ccccc2C(=O)C[C@@]1(c1ccccc1)/C=C/C(=C)C	-6.4
129822386	Brc1c(c(c(c2c(=O)cc(oc12)c1ccccc1)OC(=O)C)OC(=O)C)OC(=O)C	-6.4
132281498	O1c2cc(c(c(c2C(=O)C([C@]1(c1ccccc1)OC)(OC)OC)OC)OC)OC	-6.4
25067674	c1(cc(=O)c2c(cc(c(c2o1)[C@@H]1CCN(C)C[C@@H]1O)OC)OC)c1c(Cl)cccc1	-6.4
49800274	o1c(cc(=O)c2c(cc(c(c12)C1=CCN(C)CC1)OC)OC)c1ccccc1	-6.4
90690372	O1c2ccccc2C(=O)C([C@@]1(c1ccccc1)OC)(OC)OC	-6.4
138454529	O1c2ccccc2C(=O)[C@H]([C@]1(c1ccccc1)OC(O)(O)O)O	-6.3
155365	O1c2cc(c3c(=O)cc(oc3c2[C@@H](C1)[C@H](C(C)(C)OC(=O)C)OC(=O)C)c1ccccc1)OC	-6.3
44257645	O1c2cc(c3c(=O)cc(oc3c2[C@@H](C1)[C@@H](C(C)(C)OC(=O)C)OC(=O)C)c1ccccc1)OC	-6.3
87771765	[C@H]1(C(=O)c2ccccc2O[C@]1(c1ccccc1)C(=O)c1cc(c(c(c1)O)O)O)OS(=O)(=O)O	-6.3
12971327	Cl[C@@]1(CC(=S)c2ccccc2O1)c1ccccc1	-6.2

4		
129889097	O1c2cccc(C)c2[C@](C)([C@@](C)([C@]1(C)c1ccccc1)O)O	-6.2
129822390	o1c(cc(=O)c2c(c(c(c(c12)N(=O)=O)OC(=O)C)OC(=O)C)OC(=O)C)c1ccccc1	-6.1
16092266	Clc1cc(ncn1)N1CCNCC1	-5.9
9262	n1cncnc1	-3.3
